# Supplementary material for: Testing and controlling for horizontal pleiotropy with probabilistic Mendelian randomization in transcriptome-wide association studies
Source: Nat Commun. 2020 Jul 31;11:3861. doi: 10.1038/s41467-020-17668-6 (PMC7395774; doi:10.1038/s41467-020-17668-6)
Supplement: Supplementary file 1 — Supplementary Information [file 41467_2020_17668_MOESM1_ESM.pdf]

Supplementary Information

Testing and controlling for horizontal pleiotropy with probabilistic Mendelian randomization in transcriptome-wide association studies

Yuan et al.

Supplementary Notes

1. Model and likelihood

The proposed model is

$$\mathbf{x} = \boldsymbol{\mu}_x + Z_x\boldsymbol{\beta} + \boldsymbol{\varepsilon}_x \qquad (1)$$

$$\mathbf{y} = \boldsymbol{\mu}_y + Z_y\boldsymbol{\beta}\alpha + Z_y\boldsymbol{\gamma} + \boldsymbol{\varepsilon}_y \quad (2)$$

Where the Equation (1) is for the gene expression data and the Equation (2) is for the GWAS data. Here, $\boldsymbol{\mu}_x$ and $\boldsymbol{\mu}_y$ are the intercepts for the two models, respectively; $\boldsymbol{\beta}$ is a *p*-vector of instrumental effect sizes on the explanatory variable; α is a scalar that represents the causal effect of the explanatory variable on the outcome variable; $\boldsymbol{\gamma}$ is a *p*-vector of horizontal pleiotropic effect sizes of *p* instruments on the outcome variable; $\boldsymbol{\varepsilon}_x$ is an n_1-vector of residual error with each element independently and identically distributed from a normal distribution $\mathrm{N}(\mathbf{0}, \sigma_x^2)$; and $\boldsymbol{\varepsilon}_y$ is an n_2-vector of residual error with each element independently and identically distributed from a normal distribution $\mathrm{N}\left(\mathbf{0}, \sigma_y^2\right)$. We note that while the above two equations are specified based on two separate studies, they are joined together with the common parameter $\boldsymbol{\beta}$. We assume $\boldsymbol{\beta} \sim N\left(\mathbf{0}, \sigma_z^2 I_p\right)$.

Given $\boldsymbol{\beta}$, we have $\mathbf{x} \sim N\left(\boldsymbol{\mu}_x + Z_x\boldsymbol{\beta}, \sigma_x^2 I_{n_1}\right), \mathbf{y} \sim N\left(\boldsymbol{\mu}_y + Z_y\boldsymbol{\beta}\alpha + Z_y\boldsymbol{\gamma}, \sigma_y^2 I_{n_2}\right)$

Given Z_x, Z_y, the observed data are $(\mathbf{x}, \mathbf{y})$ and the observed likelihood function is

$$f(\mathbf{x}, \mathbf{y}) = \int f(\mathbf{x}, \mathbf{y}, \boldsymbol{\beta})d\boldsymbol{\beta} = \int f(\mathbf{x}, \mathbf{y}|\boldsymbol{\beta})f(\boldsymbol{\beta})d\boldsymbol{\beta} = \int f(\mathbf{y}|\boldsymbol{\beta})f(\mathbf{x}|\boldsymbol{\beta})f(\boldsymbol{\beta})d\boldsymbol{\beta}$$

the last equality is due to that given $\boldsymbol{\beta}$, $\mathbf{y}$ and $\mathbf{x}$ are independent.

While $f(\mathbf{y}|\boldsymbol{\beta})f(\mathbf{x}|\boldsymbol{\beta})f(\boldsymbol{\beta}) = (2\pi)^{-\frac{n_2}{2}}(\sigma_y^2)^{-\frac{n_2}{2}}\exp\left(-\frac{(\mathbf{y}-\boldsymbol{\mu}_y-\alpha Z_y\boldsymbol{\beta}-Z_y\boldsymbol{\gamma})^T(\mathbf{y}-\boldsymbol{\mu}_y-\alpha Z_y\boldsymbol{\beta}-Z_y\boldsymbol{\gamma})}{2\sigma_y^2}\right)\cdot$

$$(2\pi)^{-\frac{n_1}{2}}(\sigma_x^2)^{-\frac{n_1}{2}}\exp\left(-\frac{(\mathbf{x}-\boldsymbol{\mu}_x-Z_x\boldsymbol{\beta})^T(\mathbf{x}-\boldsymbol{\mu}_x-Z_x\boldsymbol{\beta})}{2\sigma_x^2}\right)(2\pi)^{-\frac{p}{2}}(\sigma_z^2)^{-\frac{p}{2}}\exp\left(\frac{\boldsymbol{\beta}^T\boldsymbol{\beta}}{2\sigma_z^2}\right)$$

$$= C\cdot\exp\left(-\frac{(\mathbf{y}-\boldsymbol{\mu}_y-Z_y\boldsymbol{\gamma})^T(\mathbf{y}-\boldsymbol{\mu}_y-Z_y\boldsymbol{\gamma})}{2\sigma_y^2}\right)\exp\left(-\frac{(\mathbf{x}-\boldsymbol{\mu}_x)^T(\mathbf{x}-\boldsymbol{\mu}_x)}{2\sigma_x^2}\right)$$

$$\cdot\exp\left(-\frac{\boldsymbol{\beta}^TK\boldsymbol{\beta} - 2\left(\frac{(\mathbf{y}-\boldsymbol{\mu}_y-Z_y\boldsymbol{\gamma})^T\alpha Z_y}{\sigma_y^2} + \frac{(\mathbf{x}-\boldsymbol{\mu}_x)^T Z_x}{\sigma_x^2}\right)\boldsymbol{\beta}}{2}\right)$$

Where $C = (2\pi)^{-\frac{n_2}{2}}(\sigma_y^2)^{-\frac{n_2}{2}}(2\pi)^{-\frac{n_1}{2}}(\sigma_x^2)^{-\frac{n_1}{2}}(2\pi)^{-\frac{p}{2}}(\sigma_z^2)^{-\frac{p}{2}}$,

$$K_{p\times p} = \frac{\alpha^2 Z_y^T Z_y}{\sigma_y^2} + \frac{Z_x^T Z_x}{\sigma_x^2} + \frac{1}{\sigma_z^2}I_p$$

Let $S = K^{-1}$

The above formula is equal to

$$C\cdot\exp\left(-\frac{(\mathbf{y}-\boldsymbol{\mu}_y-Z_y\boldsymbol{\gamma})^T(\mathbf{y}-\boldsymbol{\mu}_y-Z_y\boldsymbol{\gamma})}{2\sigma_y^2}\right).\exp\left(-\frac{(\mathbf{x}-\boldsymbol{\mu}_x)^T(\mathbf{x}-\boldsymbol{\mu}_x)}{2\sigma_x^2}\right)$$

$$\cdot\exp\left(-\frac{\boldsymbol{\beta}S^{-1}\boldsymbol{\beta} - 2Q^TS^{-1}\boldsymbol{\beta}}{2}\right)$$

where

$$Q_{1\times p}^T = \left(\frac{(\mathbf{y}-\boldsymbol{\mu}_y - Z_y\boldsymbol{\gamma})^T\alpha Z_y}{\sigma_y^2} + \frac{(\mathbf{x}-\boldsymbol{\mu}_x)^T Z_x}{\sigma_x^2}\right)\cdot S$$

Note that the last term is a Gaussian kernel

Thus

$$f(\mathbf{x},\mathbf{y}) = \int f(\mathbf{y}|\boldsymbol{\beta})f(\mathbf{x}|\boldsymbol{\beta})f(\boldsymbol{\beta})d\boldsymbol{\beta}$$

$$= C\cdot\exp\left(-\frac{(\mathbf{y}-\boldsymbol{\mu}_y - Z_y\boldsymbol{\gamma})^T(\mathbf{y}-\boldsymbol{\mu}_y - Z_y\boldsymbol{\gamma})}{2\sigma_y^2}\right).\exp\left(-\frac{(\mathbf{x}-\boldsymbol{\mu}_x)^T(\mathbf{x}-\boldsymbol{\mu}_x)}{2\sigma_x^2}\right)\cdot\exp\left(\frac{Q^TKQ}{2}\right)$$

$$\cdot(2\pi)^{\frac{p}{2}}\cdot|S|^{\frac{1}{2}}$$

$$= (2\pi)^{-\frac{n_2}{2}}(\sigma_y^2)^{-\frac{n_2}{2}}(2\pi)^{-\frac{n_1}{2}}(\sigma_x^2)^{-\frac{n_1}{2}}(\sigma_z^2)^{-\frac{p}{2}}|K|^{-\frac{1}{2}}\exp\left(-\frac{(\mathbf{y}-\boldsymbol{\mu}_y - Z_y\boldsymbol{\gamma})^T(\mathbf{y}-\boldsymbol{\mu}_y - Z_y\boldsymbol{\gamma})}{2\sigma_y^2}\right)$$

$$.\exp\left(-\frac{(\mathbf{x}-\boldsymbol{\mu}_x)^T(\mathbf{x}-\boldsymbol{\mu}_x)}{2\sigma_x^2}\right)$$

$$.\exp\left(\frac{\left(\frac{(\mathbf{y}-\boldsymbol{\mu}_y - Z_y\boldsymbol{\gamma})^T\alpha Z_y}{\sigma_y^2} + \frac{(\mathbf{x}-\boldsymbol{\mu}_x)^T Z_x}{\sigma_x^2}\right)K^{-1}\left(\frac{(\mathbf{y}-\boldsymbol{\mu}_y - Z_y\boldsymbol{\gamma})^T\alpha Z_y}{\sigma_y^2} + \frac{(\mathbf{x}-\boldsymbol{\mu}_x)^T Z_x}{\sigma_x^2}\right)^T}{2}\right)$$

Let $\boldsymbol{\theta} = (\alpha, \gamma, \sigma_y^2, \sigma_x^2, \sigma_z^2, \boldsymbol{\mu}_x, \boldsymbol{\mu}_y)$ indicate all model parameters.

The hypothesis test for α is $H_0: \alpha = 0\ v.s. H_1: \alpha \neq 0$

The likelihood ratio test (LRT) is given by

$$\Lambda_\alpha = 2\left\{logf(\mathbf{x},\mathbf{y}|Z_x, Z_y, \widehat{\boldsymbol{\theta}}) - logf(\mathbf{x},\mathbf{y}|Z_x, Z_y, \widehat{\boldsymbol{\theta}}_{\alpha=0})\right\}$$

Where $\widehat{\boldsymbol{\theta}}$ is the parameter estimator, and $\widehat{\boldsymbol{\theta}}_{\alpha=0}$ is the estimator under $\alpha = 0$. Similarly, the hypothesis test for γ is $H_0: \gamma = 0\ v.s.\ H_1: \gamma \neq 0$

The LRT is given by

$$\Lambda_\gamma = 2\left\{logf(\mathbf{x},\mathbf{y}|Z_x, Z_y, \widehat{\boldsymbol{\theta}}) - logf(\mathbf{x},\mathbf{y}|Z_x, Z_y, \widehat{\boldsymbol{\theta}}_{\gamma=0})\right\}$$

Where $\widehat{\boldsymbol{\theta}}_{\gamma=0}$ is the parameter estimator under $\gamma = 0$.

2. Estimation procedure

We develop an expectation-maximization (EM) algorithm for inference, where we treat the SNP effect sizes $\boldsymbol{\beta}$ as missing data. Traditional EM algorithm converges very slowly while Newton's method may be unstable and sensitive to initial values. Therefore, we use a parameter-expanded version of EM, i.e. PX-EM[1], for estimation. PX-EM improves the convergence rate of traditional EM algorithm while is simple to implement and enjoys the stability of traditional EM. To do so, we consider the parameter expanded version of our model as follows

$$\mathbf{x} = \boldsymbol{\mu}_x + \lambda Z_x\boldsymbol{\beta} + \boldsymbol{\varepsilon}_x \quad (3)$$

$$\mathbf{y} = \boldsymbol{\mu}_y + Z_y\boldsymbol{\beta}\alpha + Z_y\boldsymbol{\gamma} + \boldsymbol{\varepsilon}_y \quad (4)$$

Where λ is the expanded parameter. Let $\boldsymbol{\theta} = (\lambda, \alpha, \gamma, \sigma_y^2, \sigma_x^2, \sigma_z^2, \boldsymbol{\mu}_x, \boldsymbol{\mu}_y)$ denote all parameters

for parameter expanded model. When $\lambda = 1$, the expanded model is equal to the model for the observated data. The reduction function can be defined as $R(\lambda, \alpha, \gamma, \sigma_y^2, \sigma_x^2, \sigma_z^2, \boldsymbol{\mu}_x, \boldsymbol{\mu}_y) = \left({}^{\alpha}/_{\lambda}, \gamma, \sigma_y^2, \sigma_x^2, \lambda^2\sigma_z^2, \boldsymbol{\mu}_x, \boldsymbol{\mu}_y\right)$.

From the derivation similar as above, it is easy to obtain that, given $\mathbf{x}$, $\mathbf{y}$, Z_x, Z_y and $\boldsymbol{\theta}$, the distribution of the latent variable $\boldsymbol{\beta}$ is a normal distribution $\mathrm{N}(\boldsymbol{\beta}|\boldsymbol{\mu}_\beta, \Sigma_\beta)$, where

$$\Sigma_\beta^{-1} = \left(\frac{\alpha^2 Z_y^T Z_y}{\sigma_y^2} + \frac{\lambda^2 Z_x^T Z_x}{\sigma_x^2} + \frac{1}{\sigma_z^2} I_p\right)$$

$$\boldsymbol{\mu}_\beta = \left(\frac{\alpha^2 Z_y^T Z_y}{\sigma_y^2} + \frac{\lambda^2 Z_x^T Z_x}{\sigma_x^2} + \frac{1}{\sigma_z^2} I_p\right)^{-1} \left(\frac{\lambda}{\sigma_x^2} Z_x^T(\mathbf{x} - \boldsymbol{\mu}_x) + \frac{\alpha}{\sigma_y^2} Z_y^T(\mathbf{y} - \boldsymbol{\mu}_y - Z_y\boldsymbol{\gamma})\right)$$

The complete likelihood for the parameter expanded model can be calculated as

$$f(\mathbf{y}|\boldsymbol{\beta})f(\mathbf{x}|\boldsymbol{\beta})f(\boldsymbol{\beta}) = (2\pi)^{-\frac{n_2}{2}}(\sigma_y^2)^{-\frac{n_2}{2}}\exp\left(-\frac{(\mathbf{y}-\boldsymbol{\mu}_y-Z_y\boldsymbol{\beta}\alpha-Z_y\boldsymbol{\gamma})^T(\mathbf{y}-\boldsymbol{\mu}_y-Z_y\boldsymbol{\beta}\alpha-Z_y\boldsymbol{\gamma})}{2\sigma_y^2}\right)\cdot$$

$$(2\pi)^{-\frac{n_1}{2}}(\sigma_x^2)^{-\frac{n_1}{2}}\exp\left(-\frac{(\mathbf{x}-\boldsymbol{\mu}_x-\lambda Z_x\boldsymbol{\beta})^T(\mathbf{x}-\boldsymbol{\mu}_x-\lambda Z_x\boldsymbol{\beta})}{2\sigma_x^2}\right)(2\pi)^{-\frac{p}{2}}(\sigma_z^2)^{-\frac{p}{2}}\exp\left(\frac{\boldsymbol{\beta}^T\boldsymbol{\beta}}{2\sigma_z^2}\right)$$

In the E-step, we derive $\boldsymbol{Q}$ function by taking expectation of the complete-data log-likelihood with respect to the distribution $\mathrm{N}(\boldsymbol{\beta}|\boldsymbol{\mu}_\beta, \Sigma_\beta)$. Remember that $E(\boldsymbol{\beta}^T A\boldsymbol{\beta}) = \boldsymbol{\mu}_\beta^T A\boldsymbol{\mu}_\beta + Tr(A\Sigma_\beta)$ for any symmetric matrix *A*, where $\mathrm{Tr}(M)$ denotes the trace of matrix *M*. We can get

$$E\left((\mathbf{x}-\boldsymbol{\mu}_x-\lambda Z_x\boldsymbol{\beta})^T(\mathbf{x}-\boldsymbol{\mu}_x-\lambda Z_x\boldsymbol{\beta})\right)$$
$$= \left(\mathbf{x}-\boldsymbol{\mu}_x-\lambda Z_x\boldsymbol{\mu}_\beta\right)^T\left(\mathbf{x}-\boldsymbol{\mu}_x-\lambda Z_x\boldsymbol{\mu}_\beta\right) + \lambda^2 Tr\left(Z_x^T Z_x \Sigma_\beta\right)$$

$$E\left((\mathbf{y}-\boldsymbol{\mu}_y-Z_y\boldsymbol{\beta}\alpha-Z_y\boldsymbol{\gamma})^T(\mathbf{y}-\boldsymbol{\mu}_y-Z_y\boldsymbol{\beta}\alpha-Z_y\boldsymbol{\gamma})\right)$$
$$= \left(\mathbf{y}-\boldsymbol{\mu}_y-Z_y\boldsymbol{\gamma}-\alpha Z_y\boldsymbol{\mu}_\beta\right)^T\left(\mathbf{y}-\boldsymbol{\mu}_y-Z_y\boldsymbol{\gamma}-\alpha Z_y\boldsymbol{\mu}_\beta\right) + \alpha^2 Tr\left(Z_y^T Z_y\Sigma_\beta\right)$$

$$E(\boldsymbol{\beta}^T\boldsymbol{\beta}) = \boldsymbol{\mu}_\beta^T\boldsymbol{\mu}_\beta + Tr(\Sigma_\beta)$$

Given the current value $\boldsymbol{\theta}_{old}$ and the observed data, the $\boldsymbol{Q}$ function is

$$\boldsymbol{Q}(\boldsymbol{\theta}|\boldsymbol{\theta}_{old}) = -\frac{n_1}{2}\log\sigma_x^2 - \frac{n_2}{2}\log\sigma_y^2 - \frac{p}{2}\log\sigma_z^2 - \frac{1}{2\sigma_z^2}\boldsymbol{\mu}_\beta^T\boldsymbol{\mu}_\beta$$
$$-\frac{1}{2\sigma_x^2}\left[\left(\mathbf{x}-\boldsymbol{\mu}_x-\lambda Z_x\boldsymbol{\mu}_\beta\right)^T\left(\mathbf{x}-\boldsymbol{\mu}_x-\lambda Z_x\boldsymbol{\mu}_\beta\right)\right]$$
$$-\frac{1}{2\sigma_y^2}\left[\left(\mathbf{y}-\boldsymbol{\mu}_y-Z_y\boldsymbol{\gamma}-\alpha Z_y\boldsymbol{\mu}_\beta\right)^T\left(\mathbf{y}-\boldsymbol{\mu}_y-Z_y\boldsymbol{\gamma}-\alpha Z_y\boldsymbol{\mu}_\beta\right)\right]$$
$$-Tr\left[\left(\frac{\lambda^2}{2\sigma_x^2}Z_x^T Z_x + \frac{\alpha^2}{2\sigma_y^2}Z_y^T Z_y + \frac{1}{2\sigma_z^2}I_p\right)\Sigma_\beta\right]$$

In the M-step, by setting the derivative of $\boldsymbol{Q}$ function to zero, we obtain the new updates for all parameters. Where

$$\lambda = \frac{(\mathbf{x}-\boldsymbol{\mu}_x)^T Z_x\boldsymbol{\mu}_\beta}{\boldsymbol{\mu}_\beta^T Z_x^T Z_x\boldsymbol{\mu}_\beta + Tr\left(Z_x^T Z_x\Sigma_\beta\right)}$$

$$\gamma = \frac{\mathbf{1}_p^T Z_y^T (\mathbf{y} - \boldsymbol{\mu}_y - \alpha Z_y \boldsymbol{\mu}_\beta)}{\mathbf{1}_p^T Z_y^T Z_y \mathbf{1}_p^T}$$

$$\alpha = \frac{(\mathbf{y} - \boldsymbol{\mu}_y - Z_y \boldsymbol{\gamma})^T Z_y \boldsymbol{\mu}_\beta}{\boldsymbol{\mu}_\beta^T Z_y^T Z_y \boldsymbol{\mu}_\beta + Tr(Z_y^T Z_y \Sigma_\beta)}$$

$$\sigma_x^2 = \frac{1}{n_1}\left[(\mathbf{x} - \boldsymbol{\mu}_x - \lambda Z_x \boldsymbol{\mu}_\beta)^T (\mathbf{x} - \boldsymbol{\mu}_x - \lambda Z_x \boldsymbol{\mu}_\beta) + Tr(\lambda^2 Z_x^T Z_x \Sigma_\beta)\right]$$

$$\sigma_y^2 = \frac{1}{n_2}\left[(\mathbf{y} - \boldsymbol{\mu}_y - Z_y \boldsymbol{\gamma} - \alpha Z_y \boldsymbol{\mu}_\beta)^T (\mathbf{y} - \boldsymbol{\mu}_y - Z_y \boldsymbol{\gamma} - \alpha Z_y \boldsymbol{\mu}_\beta) + Tr(\alpha^2 Z_y^T Z_y \Sigma_\beta)\right]$$

$$\sigma_z^2 = \frac{1}{p}[\boldsymbol{\mu}_\beta^T \boldsymbol{\mu}_\beta + Tr(\Sigma_\beta)]$$

$$\mu_x = \frac{1}{n_1}(\mathbf{x} - \lambda Z_x \boldsymbol{\mu}_\beta)^T \mathbf{1}_{n_1}$$

$$\mu_y = \frac{1}{n_2}(\mathbf{y} - Z_y \boldsymbol{\gamma} - \alpha Z_y \boldsymbol{\mu}_\beta)^T \mathbf{1}_{n_2}$$

Where $\mathbf{1}_d$ denote the length *d* vector with all elements to be 1.

In the Reduction step, we re-set the estimation of parameters using the reduction function R, and re-assigned $\lambda = 1$.

Finally, for TWAS applications, we note that some genes have close to zero heritability. For genes whose expression levels are not affected by cis-SNP genotypes, not much information is available for the estimation of $\boldsymbol{\beta}$, which subsequently leads to an extremely large standard error of $\hat{\alpha}$. Therefore, PMR-Egger can return the p value equal or close to 1 for these genes.

3. Causal effect identification

The causal interpretation of the parameter α and its identification can be derived under the framework of decision-theoretic causal inference[2-5]. We define the causal effect of gene expression X on the phenotype Y as the difference between the expected values of Y under an intervention that imposes on X a reference value x_0 and another intervention that imposes another value x. Let the symbol F_X label the regime under which the value of X is generated, with $F_X = x$ indicating that X is fixed to value x by an intervention, and $F_X = \emptyset$ denoting the observational regime under which the data have actually been generated. Then the average causal effect (ACE) of X on the continuous phenotype Y is defined by

$$\mathrm{ACE} = \mathrm{E}(Y|F_X = x) - \mathrm{E}(Y|F_X = x_0)$$

Let the notation $A \perp\!\!\!\perp \mathrm{B}|\, C$ indicates that A is independent of B given C.

Our proposed MR model based on the observational data obtained under $F_X = \emptyset$ has been presented in Figure S1. Note that we directly model the horizontal pleiotropic effects through the $Z \to y$ arrow. Therefore, our model does not require the Exclusion Restriction condition of traditional MR. However, the other two assumptions in tradtional MR must be satisfied; that is, Z is associated with X, and $U \perp\!\!\!\perp Z$ **(1)**.

One must note that requirements relating only to the observational regime can never be sufficient to

estimate the causal effect of X on y, which is defined in terms of interventional regimes. Instead, we need to make additional assumptions that relate the observational regime $F_X = \emptyset$ to the interventional regimes $F_X = x$. Under the assumption that the unobserved U is a sufficient covariate for the effect of X on y, we can do this by elaborating Figure S1 to explicitly include the nonstochastic regime indicator F_X for X, as in the following Figure. For $F_X = \emptyset$, this recovers the assumptions embedded in Figure S1, but in addition it relates the observational structure to what would happen under an intervention to set X.

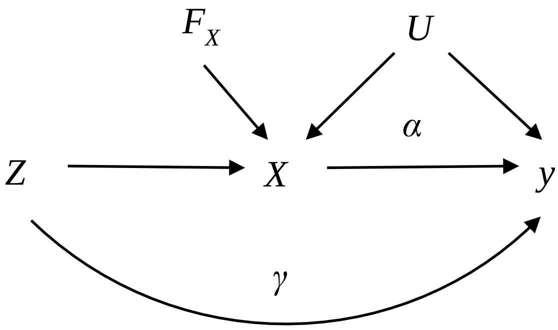

Figure. The causal diagram of PMR-Egger with the nonstochastic regime indicator F_X

It is illustrated that an intervention on X will not affect Z or U, that is $F_X \perp\!\!\!\perp (\mathrm{U}, Z)$ **(2)**. And conditional on Z and U, the distribution of y given X does not depend on whether the value of X has been generated by passive observation or intervention; that is $y \perp\!\!\!\perp F_X | (X, Z, \mathrm{U})$ **(3).** Furthermore, the formula **(1)** can be extended to $\mathrm{U} \perp\!\!\!\perp Z | F_X$ **(4)**.

We can describe the dependence of y on (X, U) (the same in all regimes by (3)) by a linear model: $\mathrm{E}(y|X, Z, \mathrm{U}) = \mathrm{W} + \alpha X + \gamma Z$ **(5)**, where W is some function of U.

Because (5) holds in the interventional regime $F_X = x$, we deduce

$$\mathrm{E}(y|F_X = x) = \mathrm{W}_0 + \alpha x + \mathrm{Z}_0$$

where $\mathrm{W}_0 := \mathrm{E}(\mathrm{W}|F_X = x)$ and $\mathrm{Z}_0 := \gamma \mathrm{E}(Z|F_X = x)$ is a constant independent of x following (2). Thus α can be interpreted causally, as it describs how the mean of y responds to manipulation of X. Next we show how to estimate α.

Again by (3), the formula (5) is also $\mathrm{E}(y|X, Z, \mathrm{U}, \mathrm{F}_X = \emptyset)$. Then

$$\mathrm{E}(y|Z, \mathrm{F}_X = \emptyset) = \mathrm{E}(\mathrm{W}|Z, \mathrm{F}_\mathrm{X} = \emptyset) + \alpha \mathrm{E}(X|Z, \mathrm{F}_\mathrm{X} = \emptyset) + \gamma Z$$

By (4), the first term on the right side is constant, thus

$\mathrm{E}(y|Z, \mathrm{F}_X = \emptyset) = \mathrm{constant} + \alpha \mathrm{E}(X|Z, \mathrm{F}_\mathrm{X} = \emptyset) + \gamma Z$ **(6)**

Equation (6) relates two functions of Z, each of which can be identified from observational data. Consequently, we can estimate the causal parameter α from such data.

4. PMR-Egger model for summary statistics

We denote the LD structure of the cis-SNPs for one specific gene as Σ_1 in gene expression data, and Σ_2 in GWAS data; both are $p \times p$ symmetric positive definite matrices. Note that Σ_1 and Σ_2

may be from the same LD reference panels. The marginal estimates of Z_x on $\mathbf{x}$ are $\widehat{\boldsymbol{\beta}}_x^*$ (eQTL effects), the marginal estimates of Z_y on $\mathbf{y}$ are $\widehat{\boldsymbol{\beta}}_y^*$ (GWAS effects). While the corresponding conditional estimates are $\widehat{\boldsymbol{\beta}}_x = \Sigma_1^{-1}\widehat{\boldsymbol{\beta}}_x^*$, $\widehat{\boldsymbol{\beta}}_y = \Sigma_2^{-1}\widehat{\boldsymbol{\beta}}_y^*$.

The corresponding model for summary statistics are

$$\begin{cases} \widehat{\boldsymbol{\beta}}_x = \boldsymbol{\beta} + E_1 & (3) \\ \widehat{\boldsymbol{\beta}}_y = \boldsymbol{\gamma} + \alpha\boldsymbol{\beta} + E & (4) \end{cases}$$

where $\boldsymbol{\beta} \sim N(\mathbf{0}, \sigma_z^2 I_p)$, $E_1 \sim N(\mathbf{0}, \Sigma_1^{-1}\sigma_x^2)$, $E \sim N\left(\mathbf{0}, \Sigma_2^{-1}\sigma_y^2\right)$, $\boldsymbol{\gamma}$ is the pleiotropy effect vector with each element equal to common parameter $\boldsymbol{\gamma}$.

Given $\boldsymbol{\beta}$, $\widehat{\boldsymbol{\beta}}_x$ and $\widehat{\boldsymbol{\beta}}_y$ are independent.

The observed likelihood function is

$$f(\widehat{\boldsymbol{\beta}}_x, \widehat{\boldsymbol{\beta}}_y) = \int f(\widehat{\boldsymbol{\beta}}_x, \widehat{\boldsymbol{\beta}}_y, \boldsymbol{\beta}) d\boldsymbol{\beta} = \int f(\widehat{\boldsymbol{\beta}}_x, \widehat{\boldsymbol{\beta}}_y|\boldsymbol{\beta}) f(\boldsymbol{\beta}) d\boldsymbol{\beta} = \int f(\widehat{\boldsymbol{\beta}}_y|\boldsymbol{\beta}) f(\widehat{\boldsymbol{\beta}}_x|\boldsymbol{\beta}) f(\boldsymbol{\beta}) d\boldsymbol{\beta}$$

$$f(\widehat{\boldsymbol{\beta}}_y|\boldsymbol{\beta}) f(\widehat{\boldsymbol{\beta}}_x|\boldsymbol{\beta}) f(\boldsymbol{\beta}) = (2\pi)^{-\frac{p}{2}} |\Sigma_1^{-1}\sigma_x^2|^{-\frac{1}{2}} \exp\left(-\frac{(\widehat{\boldsymbol{\beta}}_x - \boldsymbol{\beta})^T \Sigma_1 (\widehat{\boldsymbol{\beta}}_x - \boldsymbol{\beta})}{2\sigma_x^2}\right) (2\pi)^{-\frac{p}{2}} |\Sigma_2^{-1}\sigma_y^2|^{-\frac{1}{2}}$$

$$\exp\left(-\frac{(\widehat{\boldsymbol{\beta}}_y - \boldsymbol{\gamma} - \alpha\boldsymbol{\beta})^T \Sigma_2 (\widehat{\boldsymbol{\beta}}_y - \boldsymbol{\gamma} - \alpha\boldsymbol{\beta})}{2\sigma_y^2}\right) (2\pi)^{-\frac{p}{2}} (\sigma_z^2)^{-\frac{p}{2}} \exp\left(-\frac{\boldsymbol{\beta}^T\boldsymbol{\beta}}{2\sigma_z^2}\right)$$

$$= C \cdot \exp\left(-\frac{(\widehat{\boldsymbol{\beta}}_y - \boldsymbol{\gamma})^T \Sigma_2 (\widehat{\boldsymbol{\beta}}_y - \boldsymbol{\gamma})}{2\sigma_y^2}\right) \cdot \exp\left(-\frac{\widehat{\boldsymbol{\beta}}_x^T \Sigma_1 \widehat{\boldsymbol{\beta}}_x}{2\sigma_x^2}\right)$$

$$\cdot \exp\left(-\frac{\boldsymbol{\beta}^T K \boldsymbol{\beta} - 2\left(\frac{(\widehat{\boldsymbol{\beta}}_y - \boldsymbol{\gamma})^T \Sigma_2 \alpha}{\sigma_y^2} + \frac{\widehat{\boldsymbol{\beta}}_x^T \Sigma_1}{\sigma_x^2}\right)\boldsymbol{\beta}}{2}\right)$$

where $C = (2\pi)^{-\frac{p}{2}} |\Sigma_1^{-1}\sigma_x^2|^{-\frac{1}{2}} (2\pi)^{-\frac{p}{2}} |\Sigma_2^{-1}\sigma_y^2|^{-\frac{1}{2}} (2\pi)^{-\frac{p}{2}} (\sigma_z^2)^{-\frac{p}{2}}$, $K = \frac{1}{\sigma_z^2} I_p + \frac{\alpha^2}{\sigma_y^2}\Sigma_2 + \frac{\Sigma_1}{\sigma_x^2}$

Let $S = K^{-1}$

The above formula is equal to

$$C \cdot \exp\left(-\frac{(\widehat{\boldsymbol{\beta}}_y - \boldsymbol{\gamma})^T \Sigma_2 (\widehat{\boldsymbol{\beta}}_y - \boldsymbol{\gamma})}{2\sigma_y^2}\right) \cdot \exp\left(-\frac{\widehat{\boldsymbol{\beta}}_x^T \Sigma_1 \widehat{\boldsymbol{\beta}}_x}{2\sigma_x^2}\right) \exp\left(-\frac{\boldsymbol{\beta}^T S^{-1}\boldsymbol{\beta} - 2Q^T S^{-1}\boldsymbol{\beta}}{2}\right)$$

where $Q_{1\times p}^T = \left(\frac{(\widehat{\boldsymbol{\beta}}_y - \boldsymbol{\gamma})^T \Sigma_2 \alpha}{\sigma_y^2} + \frac{\widehat{\boldsymbol{\beta}}_x^T \Sigma_1}{\sigma_x^2}\right) \cdot S$. Thus

$$f(\widehat{\boldsymbol{\beta}}_x, \widehat{\boldsymbol{\beta}}_y) = C \cdot \exp\left(-\frac{(\widehat{\boldsymbol{\beta}}_y - \boldsymbol{\gamma})^T \Sigma_2 (\widehat{\boldsymbol{\beta}}_y - \boldsymbol{\gamma})}{2\sigma_y^2}\right) \cdot \exp\left(-\frac{\widehat{\boldsymbol{\beta}}_x^T \Sigma_1 \widehat{\boldsymbol{\beta}}_x}{2\sigma_x^2}\right) \cdot \exp\left(\frac{Q^T K Q}{2}\right) \cdot (2\pi)^{\frac{p}{2}} |S|^{\frac{1}{2}}$$

$$= (2\pi)^{-\frac{p}{2}} |\Sigma_1^{-1}\sigma_x^2|^{-\frac{1}{2}} (2\pi)^{-\frac{p}{2}} |\Sigma_2^{-1}\sigma_y^2|^{-\frac{1}{2}} (\sigma_z^2)^{-\frac{p}{2}} |K|^{-\frac{1}{2}} \exp\left(-\frac{(\widehat{\boldsymbol{\beta}}_y - \boldsymbol{\gamma})^T \Sigma_2 (\widehat{\boldsymbol{\beta}}_y - \boldsymbol{\gamma})}{2\sigma_y^2}\right)$$

$$\cdot \exp\left(-\frac{\widehat{\boldsymbol{\beta}}_x^T \Sigma_1 \widehat{\boldsymbol{\beta}}_x}{2\sigma_x^2}\right) \exp\left(\frac{\left(\frac{(\widehat{\boldsymbol{\beta}}_y - \boldsymbol{\gamma})^T \Sigma_2 \alpha}{\sigma_y^2} + \frac{\widehat{\boldsymbol{\beta}}_x^T \Sigma_1}{\sigma_x^2}\right) K^{-1} \left(\frac{(\widehat{\boldsymbol{\beta}}_y - \boldsymbol{\gamma})^T \Sigma_2 \alpha}{\sigma_y^2} + \frac{\widehat{\boldsymbol{\beta}}_x^T \Sigma_1}{\sigma_x^2}\right)^T}{2}\right)$$

Let $\boldsymbol{\Omega} = \left(\alpha, \gamma, \sigma_y^2, \sigma_x^2, \sigma_z^2\right)$ indicate all model parameters.

The hypothesis test for α is $H_0: \alpha = 0 \ v.s. H_1: \alpha \neq 0$.

The likelihood ratio test (LRT) is given by

$$\Lambda_\alpha = 2\left\{logf(\widehat{\boldsymbol{\beta}}_x, \widehat{\boldsymbol{\beta}}_y|\widehat{\boldsymbol{\Omega}}) - logf(\widehat{\boldsymbol{\beta}}_x, \widehat{\boldsymbol{\beta}}_y|\widehat{\boldsymbol{\Omega}}_{\alpha=0})\right\}$$

where $\widehat{\mathbf{\Omega}}$ is the parameter estimator, and $\widehat{\mathbf{\Omega}}_{\alpha=0}$ is the estimator under $\alpha = 0$. Similarly, the hypothesis test for γ is $H_0: \gamma = 0 \ v.s. \ H_1: \gamma \neq 0$.

The LRT is given by

$$\Lambda_\gamma = 2\left\{logf\left(\widehat{\boldsymbol{\beta}}_x, \widehat{\boldsymbol{\beta}}_y \middle| \widehat{\mathbf{\Omega}}\right) - logf\left(\widehat{\boldsymbol{\beta}}_x, \widehat{\boldsymbol{\beta}}_y \middle| \widehat{\mathbf{\Omega}}_{\gamma=0}\right)\right\}$$

where $\widehat{\mathbf{\Omega}}_{\gamma=0}$ is the parameter estimator under $\gamma = 0$.

5. PX-EM algorithm for summary statistics

The parameter expanded version of our model is

$$\begin{cases} \widehat{\boldsymbol{\beta}}_x = \lambda\boldsymbol{\beta} + E_1 & (3) \\ \widehat{\boldsymbol{\beta}}_y = \boldsymbol{\gamma} + \alpha\boldsymbol{\beta} + E & (4) \end{cases}$$

where λ is the expanded scalar parameter. Let $\mathbf{\Omega} = \left(\lambda, \alpha, \gamma, \sigma_y^2, \sigma_x^2, \sigma_z^2\right)$ denote all parameters for parameter expanded model. When $\lambda = 1$, the expanded model is equal to the model for the observated data. The reduction function can be defined as $R\left(\lambda, \alpha, \gamma, \sigma_y^2, \sigma_x^2, \sigma_z^2\right) = \left({}^{\alpha}/_{\lambda}, \gamma, \sigma_y^2, \sigma_x^2, \lambda^2\sigma_z^2\right)$.

Treating $\boldsymbol{\beta}$ as the missing variable, the complete data likelihood is

$$(2\pi)^{-\frac{p}{2}}|\Sigma_1^{-1}\sigma_x^2|^{-\frac{1}{2}} \exp\left(-\frac{\left(\widehat{\boldsymbol{\beta}}_x - \lambda\boldsymbol{\beta}\right)^T \Sigma_1 \left(\widehat{\boldsymbol{\beta}}_x - \lambda\boldsymbol{\beta}\right)}{2\sigma_x^2}\right)(2\pi)^{-\frac{p}{2}}|\Sigma_2^{-1}\sigma_y^2|^{-\frac{1}{2}}$$

$$\exp\left(-\frac{\left(\widehat{\boldsymbol{\beta}}_y - \boldsymbol{\gamma} - \alpha\boldsymbol{\beta}\right)^T \Sigma_2 \left(\widehat{\boldsymbol{\beta}}_y - \boldsymbol{\gamma} - \alpha\boldsymbol{\beta}\right)}{2\sigma_y^2}\right)(2\pi)^{-\frac{p}{2}}(\sigma_z^2)^{-\frac{p}{2}}\exp\left(-\frac{\boldsymbol{\beta}^T\boldsymbol{\beta}}{2\sigma_z^2}\right)$$

Given the data $\widehat{\boldsymbol{\beta}}_x$ and $\widehat{\boldsymbol{\beta}}_y$, and parameters $\mathbf{\Omega}$, it is easy to obtain the distribution of $\boldsymbol{\beta}$ is Gaussian with the mean

$$\boldsymbol{\mu}_\beta = \left(\frac{1}{\sigma_z^2}I_p + \frac{\alpha^2}{\sigma_y^2}\Sigma_2 + \frac{\lambda^2}{\sigma_x^2}\Sigma_1\right)^{-1} \cdot \left(\frac{\left(\widehat{\boldsymbol{\beta}}_y - \boldsymbol{\gamma}\right)^T\Sigma_2\alpha}{\sigma_y^2} + \frac{\widehat{\boldsymbol{\beta}}_x^T\lambda\Sigma_1}{\sigma_x^2}\right)^T$$

the covariance matrix $\Sigma_\beta = \left(\frac{1}{\sigma_z^2}I_p + \frac{\alpha^2}{\sigma_y^2}\Sigma_2 + \frac{\lambda^2}{\sigma_x^2}\Sigma_1\right)^{-1}$

Using formula $E(\boldsymbol{\beta}^T A\boldsymbol{\beta}) = \boldsymbol{\mu}_\beta^T A\boldsymbol{\mu}_\beta + Tr\left(A\Sigma_\beta\right)$ for any symmetrix *A*.

The *E*-step can generate the following $\mathbf{Q}$ function

$$\begin{aligned} \boldsymbol{Q}\left(\mathbf{\Omega}|\widehat{\boldsymbol{\beta}}_x, \widehat{\boldsymbol{\beta}}_y\right) = &-\frac{p}{2}log2\pi + \frac{1}{2}log|\Sigma_1| - \frac{p}{2}log\,\sigma_x^2 - \frac{p}{2}log2\pi + \frac{1}{2}log|\Sigma_2| - \frac{p}{2}log\,\sigma_y^2 - \frac{p}{2}log2\pi \\ &- \frac{p}{2}log\,\sigma_z^2 - \frac{1}{2\sigma_z^2}\left[\boldsymbol{\mu}_\beta^T\boldsymbol{\mu}_\beta + tr\left(\Sigma_\beta\right)\right] \\ &- \frac{1}{2\sigma_x^2}\left[\widehat{\boldsymbol{\beta}}_x^T\Sigma_1\widehat{\boldsymbol{\beta}}_x - 2\widehat{\boldsymbol{\beta}}_x^T\lambda\Sigma_1\boldsymbol{\mu}_\beta + \lambda^2\left(\boldsymbol{\mu}_\beta^T\Sigma_1\boldsymbol{\mu}_\beta + Tr\left(\Sigma_1\Sigma_\beta\right)\right)\right] \\ &- \frac{1}{2\sigma_y^2}\left\{\left(\widehat{\boldsymbol{\beta}}_y - \boldsymbol{\gamma}\right)^T\Sigma_2\left(\widehat{\boldsymbol{\beta}}_y - \boldsymbol{\gamma}\right) - 2\left(\widehat{\boldsymbol{\beta}}_y - \boldsymbol{\gamma}\right)^T\Sigma_2\alpha\boldsymbol{\mu}_\beta \right. \\ &\left. + \alpha^2\left[\boldsymbol{\mu}_\beta^T\Sigma_2\boldsymbol{\mu}_\beta + Tr\left(\Sigma_2\Sigma_\beta\right)\right]\right\} \end{aligned}$$

In the M-step, we set the derivative of the $\mathbf{Q}$ function to zero and obtain the following updated equations for all parameters.

$$\lambda = \frac{\widehat{\boldsymbol{\beta}}_x^T\Sigma_1\boldsymbol{\mu}_\beta}{\boldsymbol{\mu}_\beta^T\Sigma_1\boldsymbol{\mu}_\beta + Tr\left(\Sigma_1\Sigma_\beta\right)}$$

$$\alpha = \frac{\left(\widehat{\boldsymbol{\beta}}_y - \boldsymbol{\gamma}\right)^T\Sigma_2\boldsymbol{\mu}_\beta}{\boldsymbol{\mu}_\beta^T\Sigma_2\boldsymbol{\mu}_\beta + Tr\left(\Sigma_2\Sigma_\beta\right)}$$

$$\sigma_x^2 = \frac{1}{p}\left[\widehat{\boldsymbol{\beta}}_x^T \Sigma_1 \widehat{\boldsymbol{\beta}}_x - 2\widehat{\boldsymbol{\beta}}_x^T \lambda \Sigma_1 \boldsymbol{\mu}_\beta + \lambda^2 \left(\boldsymbol{\mu}_\beta^T \Sigma_1 \boldsymbol{\mu}_\beta + Tr(\Sigma_1 \Sigma_\beta)\right)\right]$$

$$\sigma_y^2 = \frac{1}{p}\left\{(\widehat{\boldsymbol{\beta}}_y - \boldsymbol{\gamma})^T \Sigma_2 (\widehat{\boldsymbol{\beta}}_y - \boldsymbol{\gamma}) - 2(\widehat{\boldsymbol{\beta}}_y - \boldsymbol{\gamma})^T \Sigma_2 \alpha \boldsymbol{\mu}_\beta + \alpha^2 \left[\boldsymbol{\mu}_\beta^T \Sigma_2 \boldsymbol{\mu}_\beta + Tr(\Sigma_2 \Sigma_\beta)\right]\right\}$$

$$\sigma_z^2 = \frac{1}{p}\left[\boldsymbol{\mu}_\beta^T \boldsymbol{\mu}_\beta + Tr(\Sigma_\beta)\right]$$

$$\gamma = \frac{\mathbf{1}_p^T \Sigma_2 (\widehat{\boldsymbol{\beta}}_y - \alpha \boldsymbol{\mu}_\beta)}{\mathbf{1}_p^T \Sigma_2 \mathbf{1}_p^T}$$

where $\mathbf{1}_p$ is a length p vector with elements equal to 1.

In the Reduction step, we re-set the estimation of parameters using the reduction function R, and re-assigned $\lambda = 1$.

6. Sparse vs polygenic modeling assumptions in previous TWAS methods

Currently, almost all previous TWAS methods (TWAS[6], PrediXcan[7], CoMM[8], DPR[9],TIGAR[10] etc.) make a polygenic modeling assumption and assume that cis-SNPs have non-zero polygenic effects on gene expression. Specifically, TWAS makes the BSLMM[11] polygenic modeling assumption: all cis-SNPs have non-zero effects and their effect sizes follow a mixture of two normal distributions. PrediXcan[7] makes the ElasticNet modeling assumption: all cis-SNPs have non-zero effects a priori and their effect sizes follows a mixture of Laplace (L1) and normal (L2) distributions. Both TIGAR and DPR makes the Bayesian non-parametric polygenic modeling assumption: all cis-SNPs have non-zero effects and their effect sizes follow a mixture of many normal distributions. CoMM makes the standard polygenic modeling assumption: all cis-SNPs have non-zero effects and their effect sizes follow a normal distribution. Perhaps the only method previously used in TWAS setting that makes a sparse modeling assumption is SMR[12]. Certainly, while the modeling assumption underlying PrediXcan is polygenic, it does use an optimization algorithm that obtains the posterior mode estimates (which is sparse) instead of the posterior mean estimates (which is polygenic). However, it is important to distinguish the modeling assumption from the inference algorithm. Overall, the polygenic modeling assumption made in most existing TWAS methods are consistent with the previously known fact that polygenic models often outperform sparse models in gene expression prediction[4,7] and are also consistent with our current results showing that polygenic models also outperform sparse models for TWAS applications.

7. Number of discoveries and the genomic control factor in real data applications

The number of genes identified above a genome-wide threshold is commonly used in the literature as a measure of statistical power in real data applications. The genomic control factor λ is commonly used in the literature to measure type I error control for real data applications, as λ captures approximately the type I error control at the level of 0.05. Power and λ are two different statistical terms that are not necessarily correlated with each other. A similar λ among different methods would suggest similar type I error control at the nominal level of 0.05, while a higher number of genes identified by one method over another would suggests its higher power. Certainly, for a data with a relatively large sample size (e.g. UK Biobank) and a trait with a highly polygenic genetic architecture, then λ may also be influenced by power of the method in addition to its type I error control. In addition, the number of significant genes may not be a perfect measure of power in certain cases and can be influenced by λ, as a method that fails to control for type I error could yield inflated *p*-values, leading to a high number of false discoveries.

8. Horizontal pleiotropic effect tests can help to explain the discrepancy in terms of the causal associations detected by PMR-Egger and the other methods

For example, for the trait of red blood cell count in UK Biobank, the *MAPT* gene on chromosome 17 shows a significant pleiotropy effect ($p = 2.35 \times 10^{-9}$) but displays no significant causal effect (p=0.98) by PMR-Egger. In contrast, *MAPT* is detected to be significantly associated with red blood cell count by PrediXcan ($p = 8.11 \times 10^{-10}$), and, to a much lesser extent, by TWAS ($p = 1.72 \times 10^{-3}$). However, no previous evidence suggests that *MAPT* is associated with red blood cell count. Indeed, we found that the genomic location of *MAPT* (43,871,748-44,205,700) is close to and partially overlapped with *KANSL1* (44,007,282-44,402,733), which has been previously identified to be associated with red blood cell traits[13,14]. The association between *KANSL1* and red blood cell count is also detected by PMR-Egger ($p = 1.02 \times 10^{-7}$), by CoMM ($p = 2.72 \times 10^{-8}$), and, to a much lesser extent, by TWAS ($p = 1.66 \times 10^{-3}$) in the present study. By controlling for the expression level of the *KANSL1* gene in the PrediXcan framework, the association between the predicted *MAPT* expression level and red blood cell count is no longer significant (p = 0.10). Therefore, the causal association between *MAPT* and red blood cell count detected by PrediXcan likely reflects either the true horizontal pleiotropic effect of *MAPT* cis-SNPs on red blood cell count through *KANSL1* or their tagging effects of the neighboring eQTLs of *KANSL1*. As another example, for height in the UK Biobank, the pseudogene *RP11-9E13.2* (70,137,755-70,340,521) on chromosome 10 has a significant pleiotropy effect ($p = 1.08 \times 10^{-13}$) but displays no significant causal effect (p=0.93) by PMR-Egger. In contrast, *RP11-9E13.2* is detected to be significantly associated with height by PrediXcan ($p = 4.34 \times 10^{-10}$), and, to a lesser extent, by TWAS ($p = 9.05 \times 10^{-6}$). The pseudogene *RP11-9E13.2* is in the neighborhood of *MYPN* (69,765,912-70,071,774), which has been previously identified to be associated with height[15]. The association between *MYPN* and height is also detected by PMR-Egger ($p = 1.82 \times 10^{-7}$), CoMM ($p = 2.13 \times 10^{-14}$), and to a lesser extent, PrediXcan ($p = 3.94 \times 10^{-4}$) and TWAS ($p = 1.55 \times 10^{-3}$), in the present study. By controlling for the predicted expression level of *MYPN* gene in the PrediXcan framework, the association between the predicted *RP11-9E13.2* expression level and height is no longer significant at the genome-wide threshold ($p = 3.37 \times 10^{-4}$). Therefore, the causal association between the pseudogene *RP11-9E13.2* and height as detected by PrediXcan and TWAS likely reflects either the horizontal pleiotropic effect of *RP11-9E13.2* cis-SNPs on height through *MYPN* or their tagging effects of the neighboring eQTLs of *MYPN*. The results suggest the practical importance of testing and controlling for pleiotropic effects in TWAS applications. Certainly, we acknowledge that, both these examples are focused on the special case where the false gene association with the trait disappears when conditional on a neighboring gene. We did not provide examples where the apparently false gene association with the trait may be explained by horizontal pleiotropic effects acted upon/through a gene far away, as it is often challenging to convincingly identify trans eQTL effects. In the special case we focused on, while it is possible that SNPs display true horizontal pleiotropic effects through the neighboring gene, it is equally likely that SNPs used in the model are simply tagging nearby eQTLs of the neighboring causal gene[16,17] and thus display apparent "horizontal pleiotropic effects" through the neighboring gene, as also mentioned above. Subsequently, the horizontal pleiotropic effect term in PMR-Egger may represent the apparent "horizontal pleiotropic effects" through SNP tagging to the nearby eQTLs of the causal gene, rather than the truly horizontal pleiotropic effect acted through other molecular pathways. Regardless of the interpretation of the horizontal pleiotropic effect term, we found it reassuring that by modeling the horizontal pleiotropic effect term in PMR-Egger can reduce false discoveries in the case of SNP tagging.

Supplementary Figures

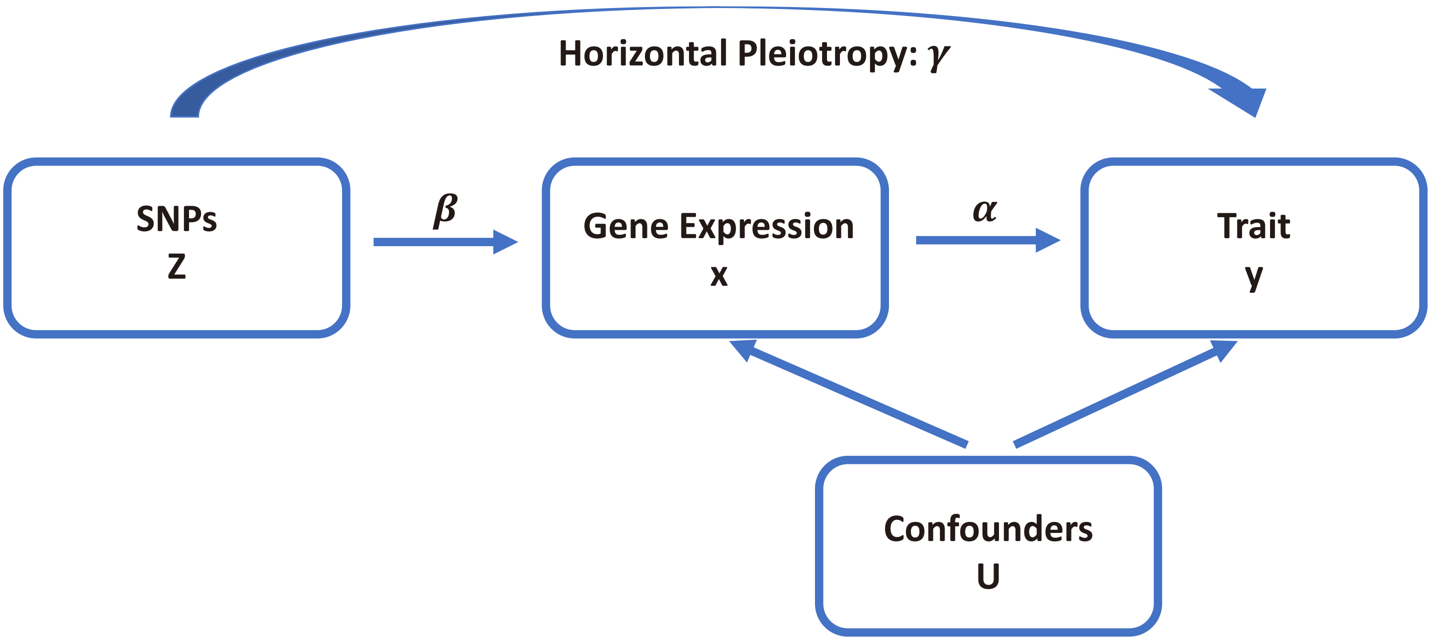

Supplementary Figure 1. An illustrative diagram for Mendelian randomization analysis. Mendelian randomization analysis in the TWAS setting attempts to estimate the causal effect of gene expression (x) on the trait of interest (y) in the presence of confounding factors (U) by using cis-SNPs (Z) as instrumental variables. An important requirement of Mendelian randomization analysis is to model and control for horizontal pleiotropic effects.

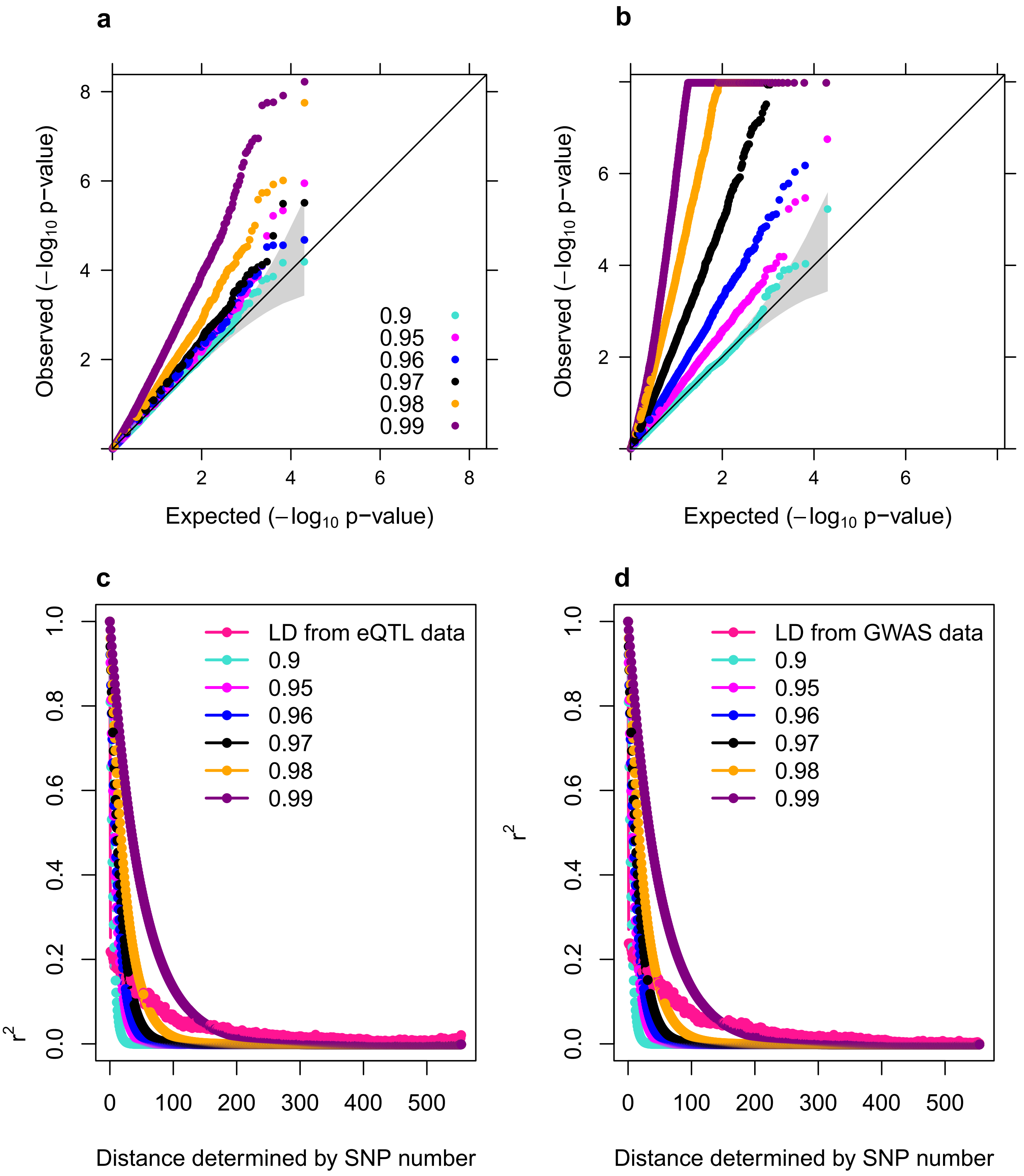

Supplementary Figure 2. Quantile-quantile plot of -log10 *p*-values from LDA MR-Egger under null simulations. LDA MR-Egger tests for either causal effect (**a**) or horizontal pleiotropic effect (**b**). We follow the same simulation design in the original LDA MR-Egger paper to perform simulations. The simulation design assumes that the SNP covariance matrix is an AR(1) covariance structure, where we set the autocorrelation parameter to be either 0.9 (turquoise), 0.95 (magenta), 0.96 (blue), 0.97 (black), 0.98 (orange), and 0.99 (purple). The *p*-values from LDA MR-Egger are reasonably well calibrated when the autocorrelation parameter is set to be moderate as used in the original paper. However, when the correlation parameter is set to be realistically high (>0.9) or if we used SNPs from real data to carry out the same set of simulations, then LDA MR-Egger p-values become inflated. The LD decay pattern under these covariance structures are plotted together with the realistic LD pattern of the *BACE1* gene (pink). The LD pattern of the *BACE1* gene is estimated either in the eQTL data (**c**) or in the GWAS data (**d**).

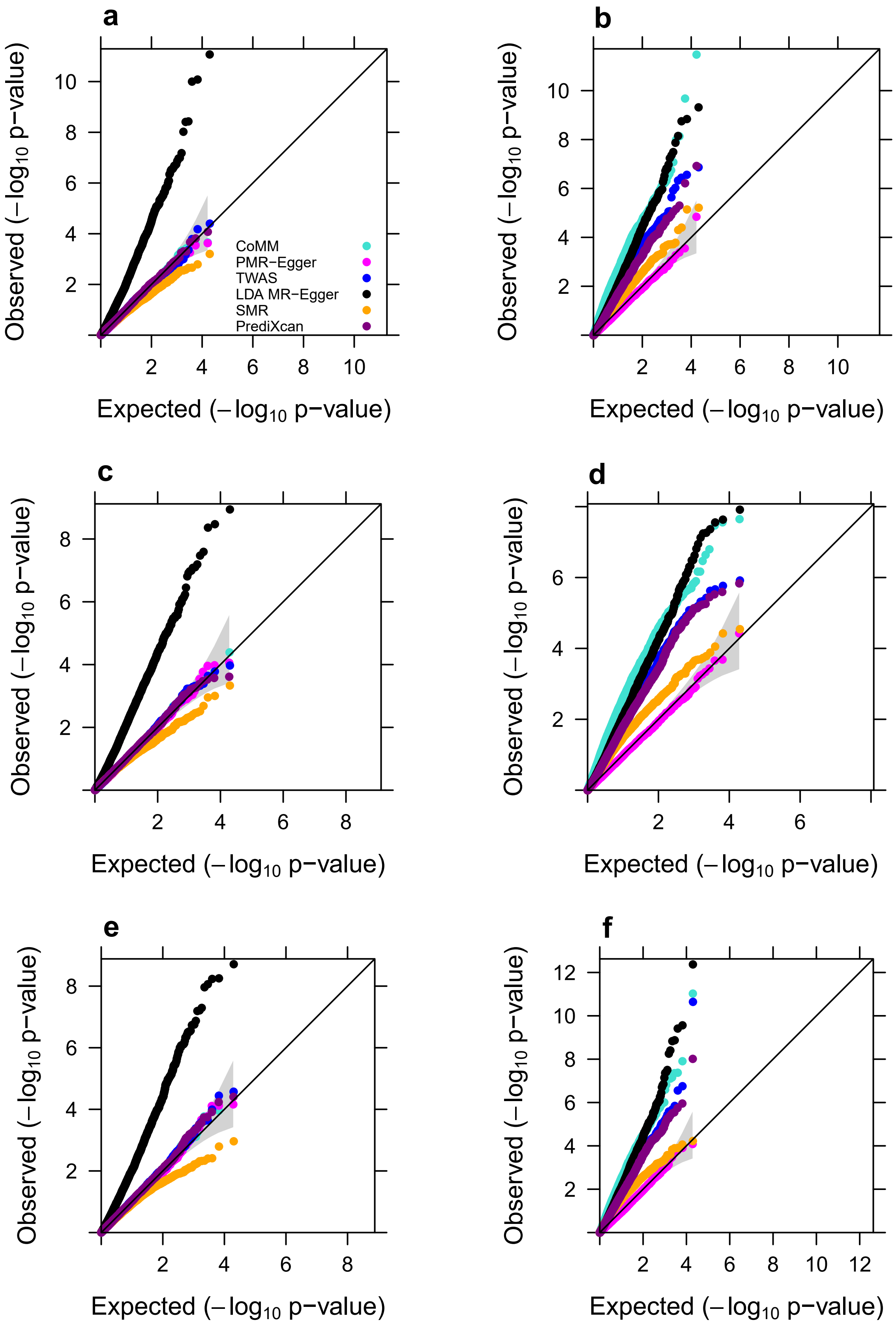

Supplementary Figure 3. Quantile-quantile plot of -log10 *p*-values from different methods for testing the causal effect under the null simulations, in various sparse settings where only a small proportion of SNPs are associated with the gene expression level. Compared methods include CoMM (turquoise), PMR-Egger (magenta), TWAS (blue), LDA MR-Egger (black), SMR (orange), and PrediXcan (purple). Simulations are performed either in the absence (γ=0; **a**, **c**, **e**) or in the presence of horizontal pleiotropic effect (γ=0.001; **b**, **d**, **f**). Either one SNP (**a**, **b**), 1% of SNPs (**c**, **d**), or 10% SNPs (**e**, **f**) have non-zero effects on gene expression. Only *p*-values from PMR-Egger adhere to the expected diagonal line across a range of settings.

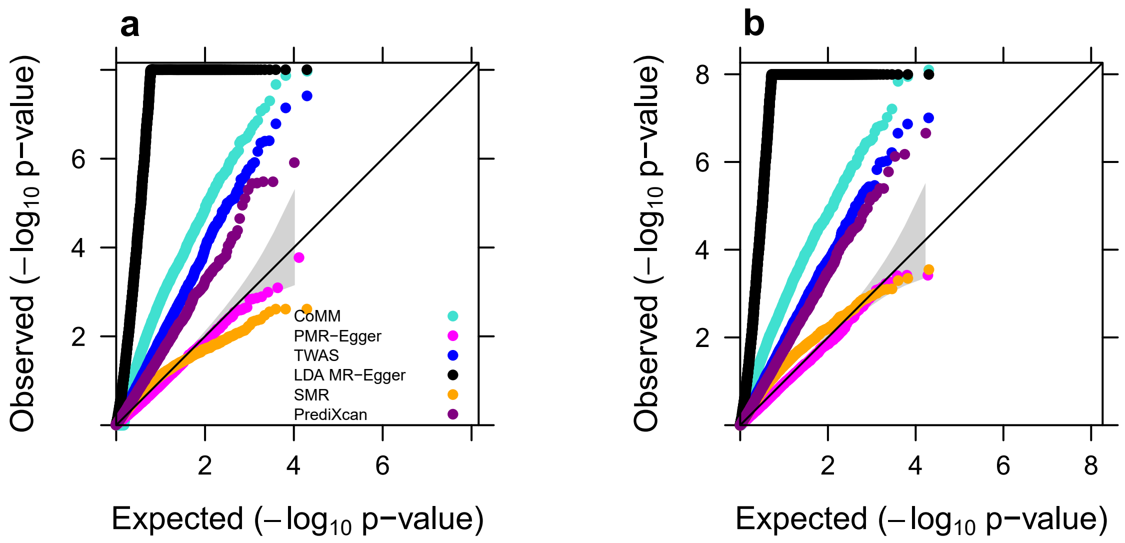

Supplementary Figure 4. Quantile-quantile plot of -log10 *p*-values from different methods for testing the causal effect under the null, across different gene expression heritability values. Compared methods include CoMM (turquoise), PMR-Egger (magenta), TWAS (blue), LDA MR-Egger (black), SMR (orange), and PrediXcan (purple). Simulations are performed or in the presence of horizontal pleiotropic effect (γ=0.001) with gene expression heritability being either (**a**) PVE_{zx}=1% or (**b**) PVE_{zx}=5%. Only *p*-values from PMR-Egger adhere to the expected diagonal line across a range of settings.

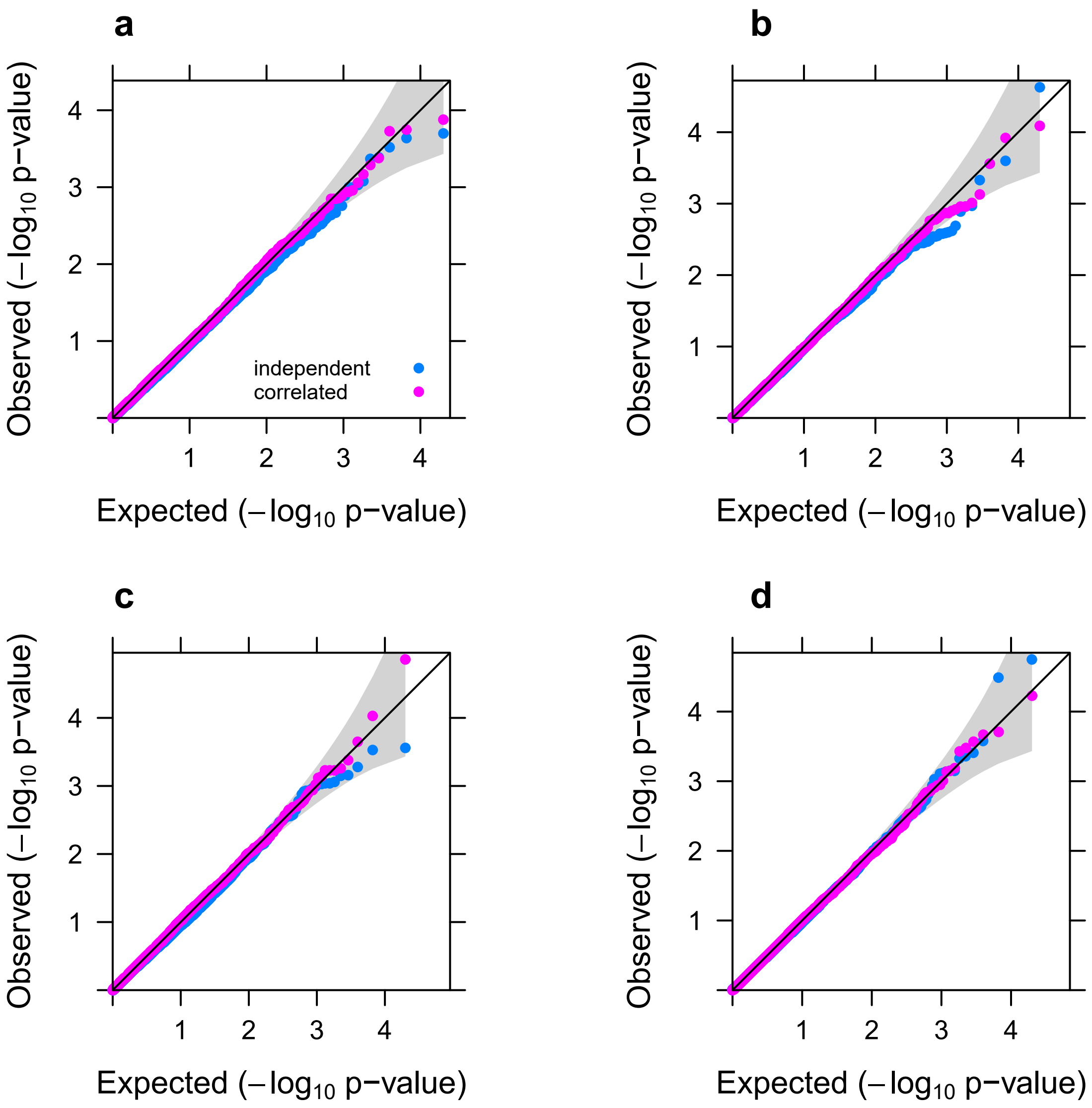

Supplementary Figure 5. Quantile-quantile plot of -log10 *p*-values of PMR-Egger in null simulations. Results are shown under simulations where the SNP effects on the gene expression are either correlated (pink) or independent (blue). The *p*-values for testing the causal effect under the null (PVE_{zy}=0) are shown in the absence (γ=0; **a**) or presence (γ=0.0005; **b**) of horizontal pleiotropic effects. The *p*-values for testing the pleotropic effect under the null (γ=0) are shown in the absence (PVE_{zy}=0; **c**) or presence (PVE_{zy}=0.6%; **d**) of causal effect.

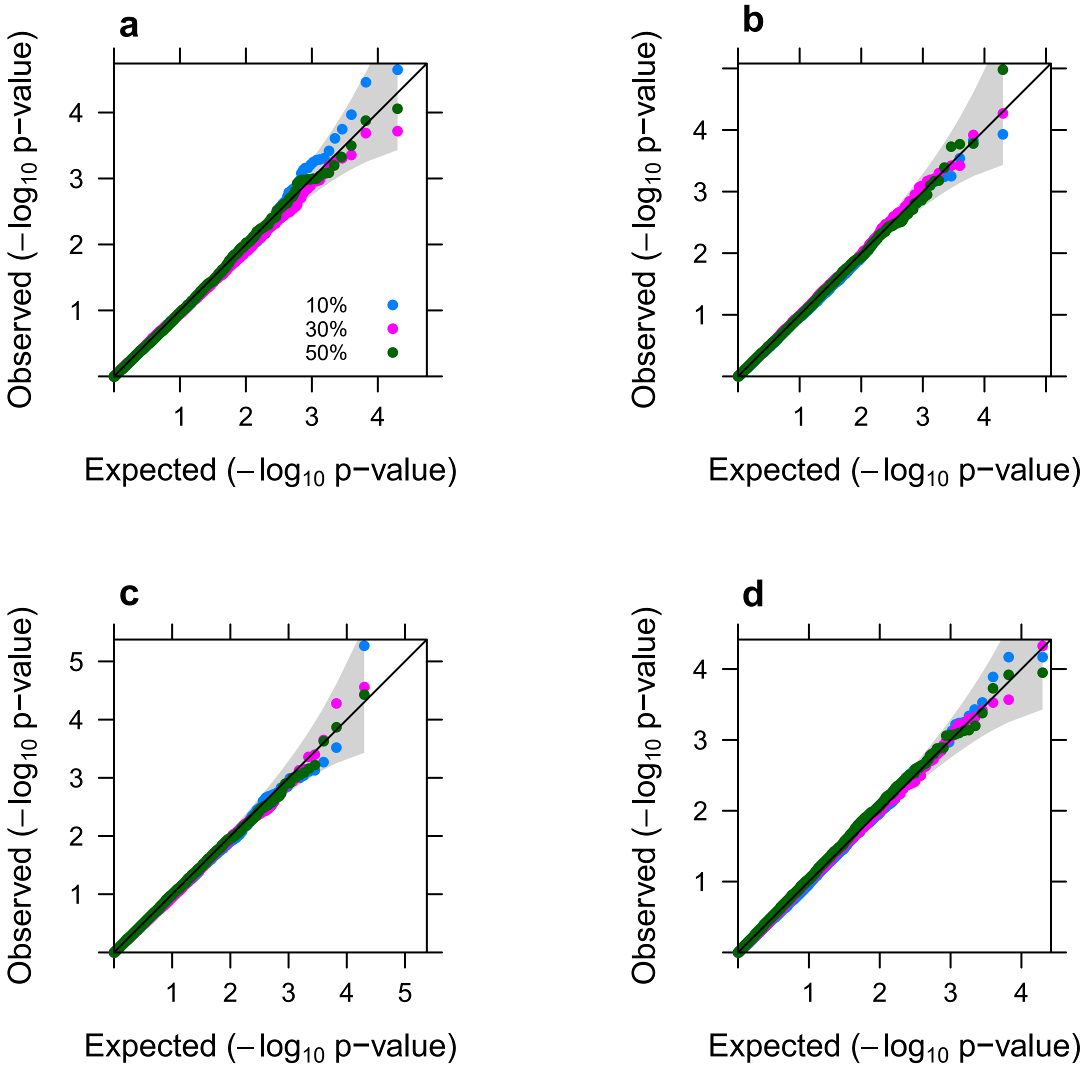

Supplementary Figure 6. Quantile-quantile plot of -log10 *p*-values from PMR-Egger for testing the causal effect under the null, under various sparse horizontal pleiotropic effect settings. Simulations are performed under different horizontal pleiotropic effect sizes: (**a**) γ=0.0001; (**b**) γ=0.0005; (**c**) γ=0.001; (**d**) γ=0.002. In each panel, only a fixed proportion of SNPs (10%, 30%, or 50%) have non-zero horizontal pleiotropic effects. *P*-values from PMR-Egger behave well across a range of sparse horizontal pleiotropic effect settings.

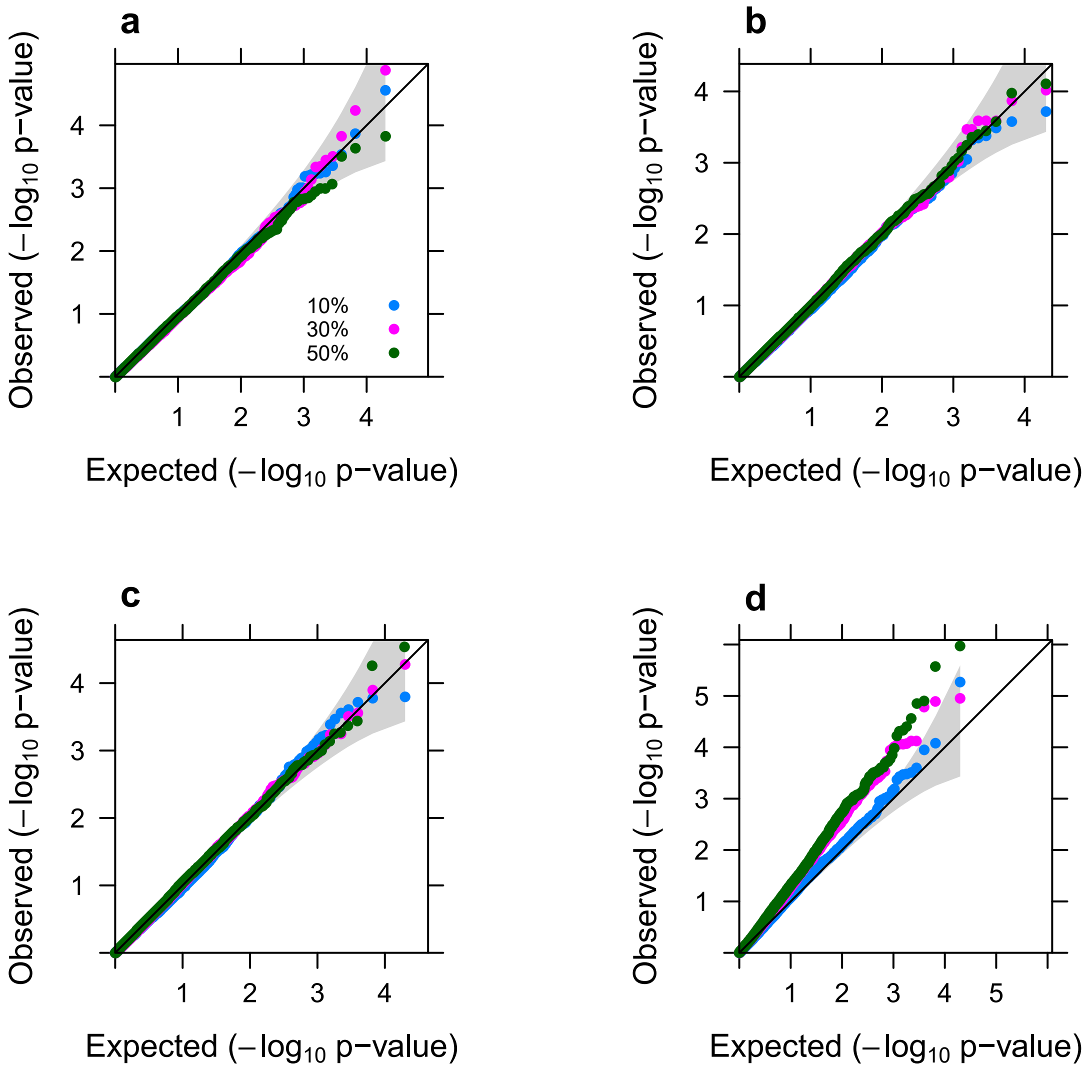

Supplementary Figure 7. Quantile-quantile plot of -log10 *p*-values from PMR-Egger for testing the causal effect under the null, under various directional horizontal pleiotropic effect assumptions. Simulations are performed under different horizontal pleiotropic effect sizes: (**a**) γ=0.0001; (**b**) γ=0.0005; (**c**) γ=0.001; (**d**) γ=0.002. In each panel, a fixed proportion of SNPs (10%, 30%, or 50%) have positive horizontal pleiotropic effects while the remaining proportion of SNPs have negative horizontal pleiotropic effects. *P*-values from PMR-Egger behave reasonably well across a range of directional or balanced horizontal pleiotropic effect settings, except in the extreme case where horizontal pleiotropic effect size is very large (γ=0.002) and where the effect size signs across SNPs are approximately balanced.

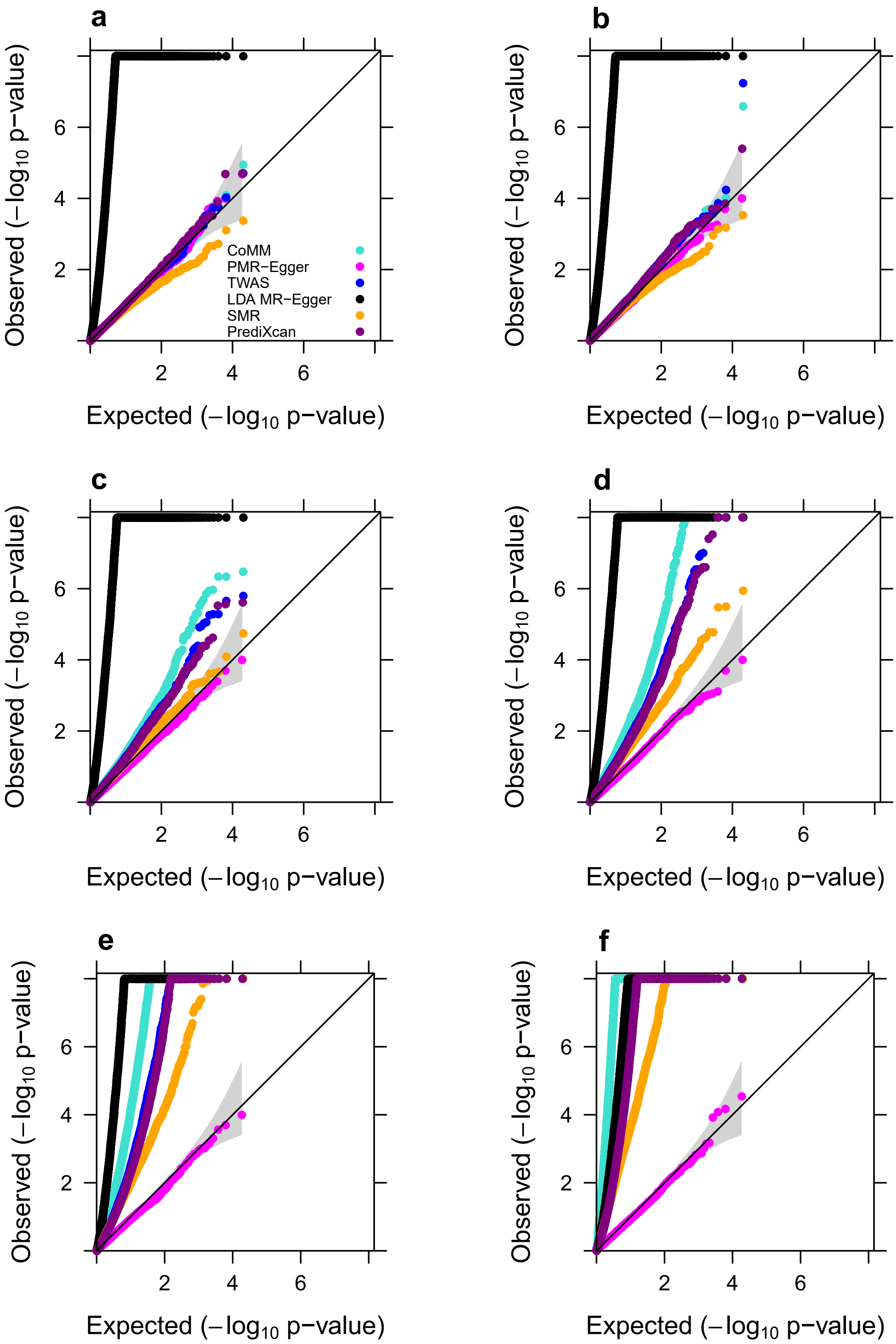

Supplementary Figure 8. Quantile-quantile plot of -log10 *p*-values from cross-gene simulations for different methods for testing the causal effect either in the absence or in the presence of horizontal pleiotropic effect under null simulations. Compared methods include CoMM (turquoise), PMR-Egger (magenta), TWAS (blue), LDA MR-Egger (black), SMR (orange), and PrediXcan (purple). Null simulations are performed under different horizontal pleiotropic effect sizes: (**a**) γ=0; (**b**) γ=0.0001; (**c**) γ=0.0002; (**d**) γ=0.0003; (**e**) γ=0.0005; (**f**) γ=0.001. Only *p*-values from PMR-Egger adhere to the expected diagonal line across a range of horizontal pleiotropic effect sizes.

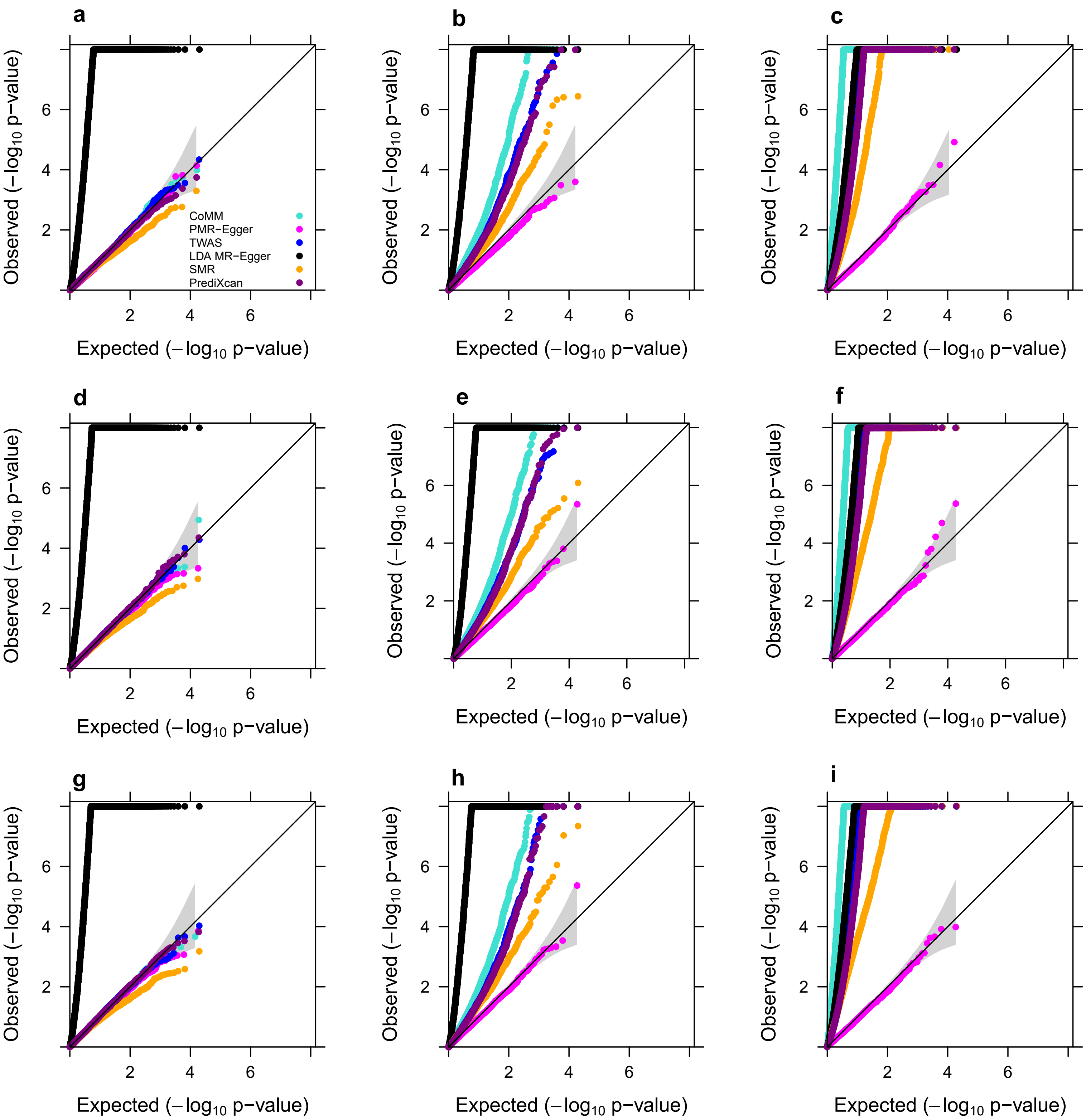

Supplementary Figure 9. Quantile-quantile plot of -log10 *p*-values from cross-gene simulations of different methods for testing the causal effect under the null simulations, in various sparse settings where only a small proportion of SNPs are associated with the gene expression level. Compared methods include CoMM (turquoise), PMR-Egger (magenta), TWAS (blue), LDA MR-Egger (black), SMR (orange), and PrediXcan (purple). Simulations are performed either in the absence (γ=0; **a**, **d**, **g**) or in the presence of horizontal pleiotropic effect (γ=0.0003; **b**, **e**, **h**; γ=0.001; **c**, **f**, **i**). Either one SNP (**a**, **b**, **c**), 1% of SNPs (**d**, **e**, **f**), or 10% SNPs (**g**, **h**, **i**) have non-zero effects on gene expression. Only *p*-values from PMR-Egger adhere to the expected diagonal line across a range of settings.

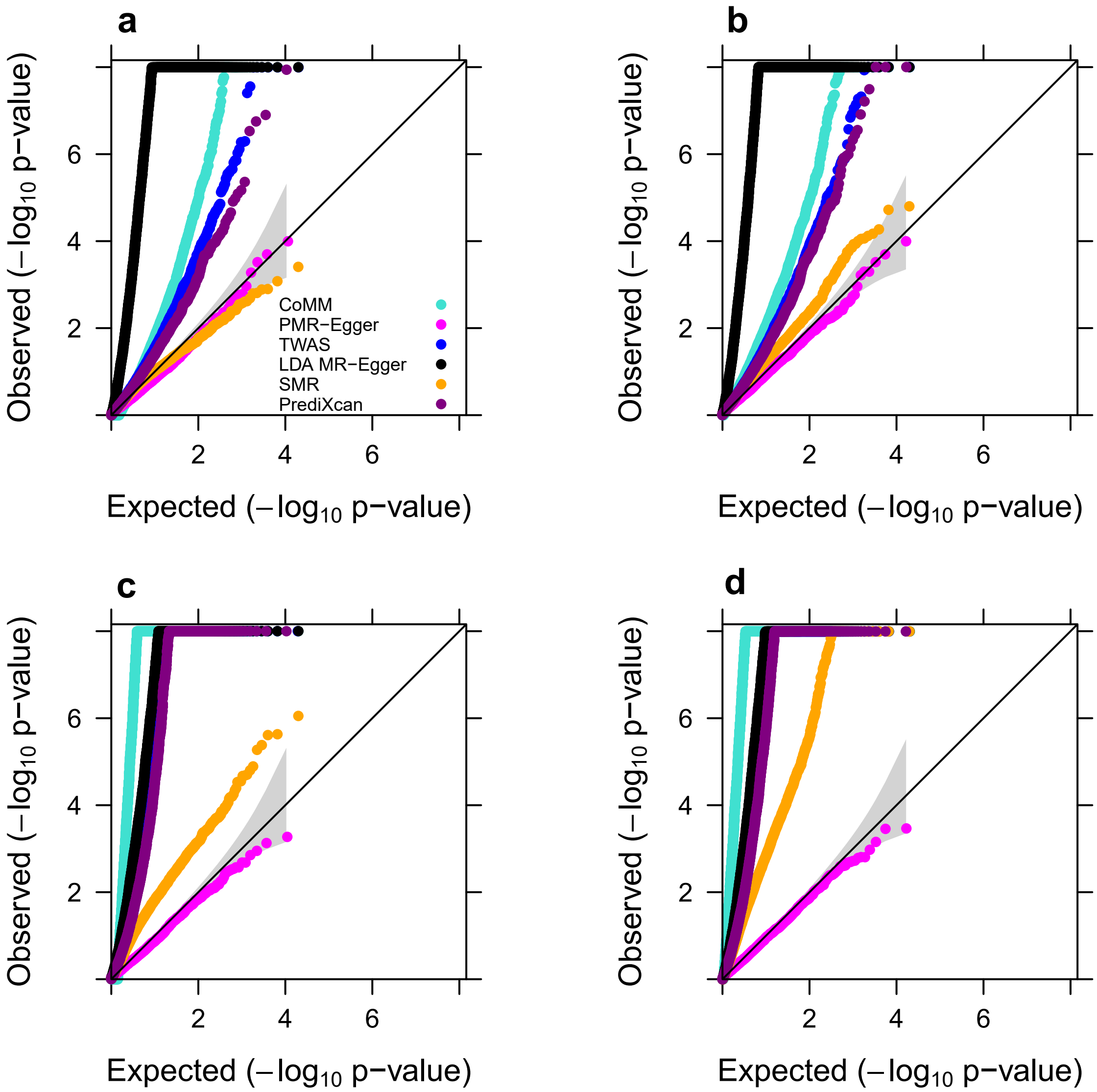

Supplementary Figure 10. Quantile-quantile plot of -log10 *p*-values from cross-gene simulations of different methods for testing the causal effect under the null, across different gene expression heritability values. Compared methods include CoMM (turquoise), PMR-Egger (magenta), TWAS (blue), LDA MR-Egger (black), SMR (orange), and PrediXcan (purple). Simulations are performed or in the presence of horizontal pleiotropic effect (γ=0.0003, **a**, **b**; γ=0.001, **c**, **d**;) with gene expression heritability being either PVE_{zx}=1% (**a**, **c**) or PVE_{zx}=5% (**b**, **d**). Only *p*-values from PMR-Egger adhere to the expected diagonal line across a range of settings

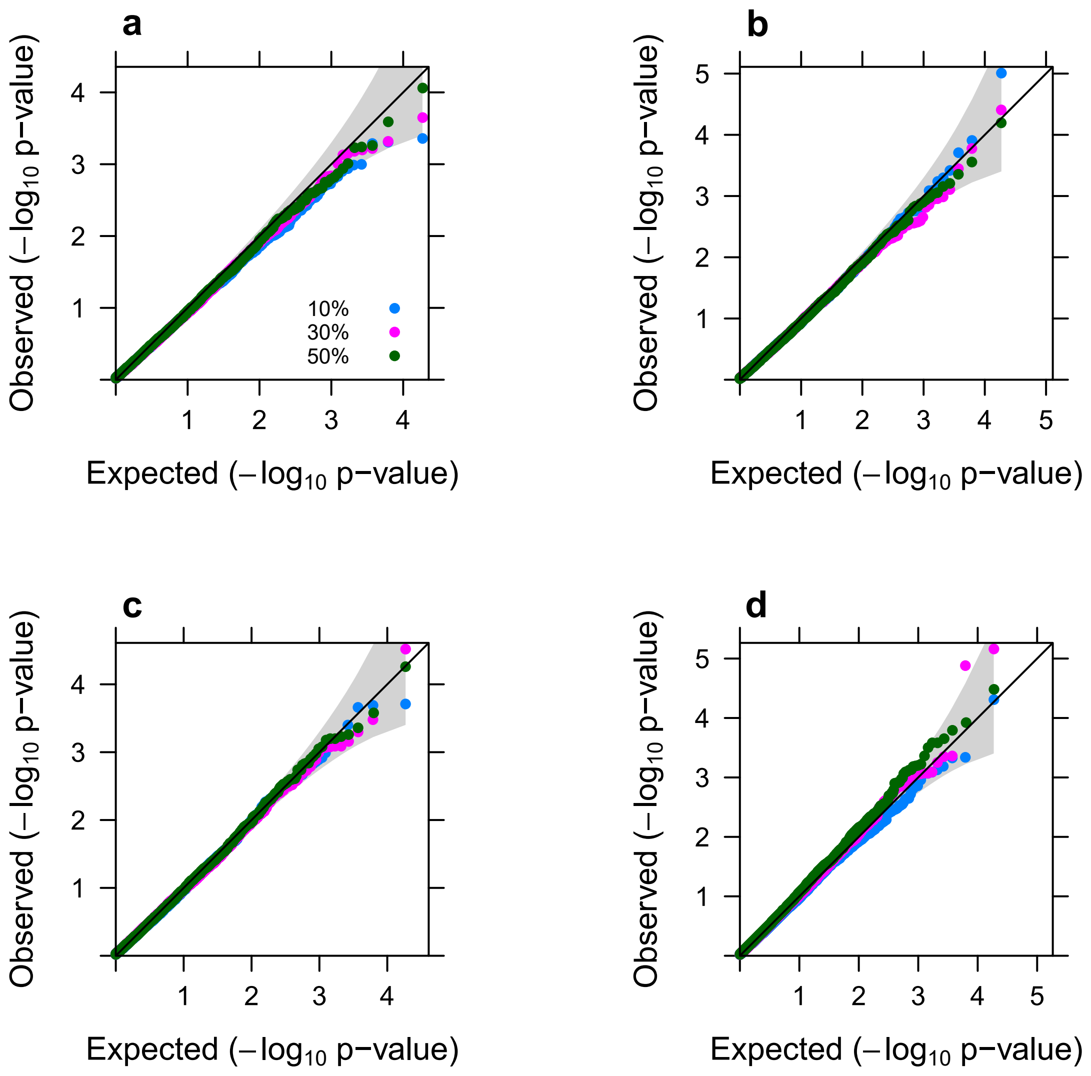

Supplementary Figure 11. Quantile-quantile plot of -log10 *p*-values from PMR-Egger under cross-gene simulations for testing the causal effect under the null, under various sparse horizontal pleiotropic effect settings. Simulations are performed under different horizontal pleiotropic effect sizes: (**a**) γ=0.0001; (**b**) γ=0.0005; (**c**) γ=0.001; (**d**) γ=0.002. In each panel, only a fixed proportion of SNPs (10%, 30%, or 50%) have non-zero horizontal pleiotropic effects. *P*-values from PMR-Egger behave well across a range of sparse horizontal pleiotropic effect settings.

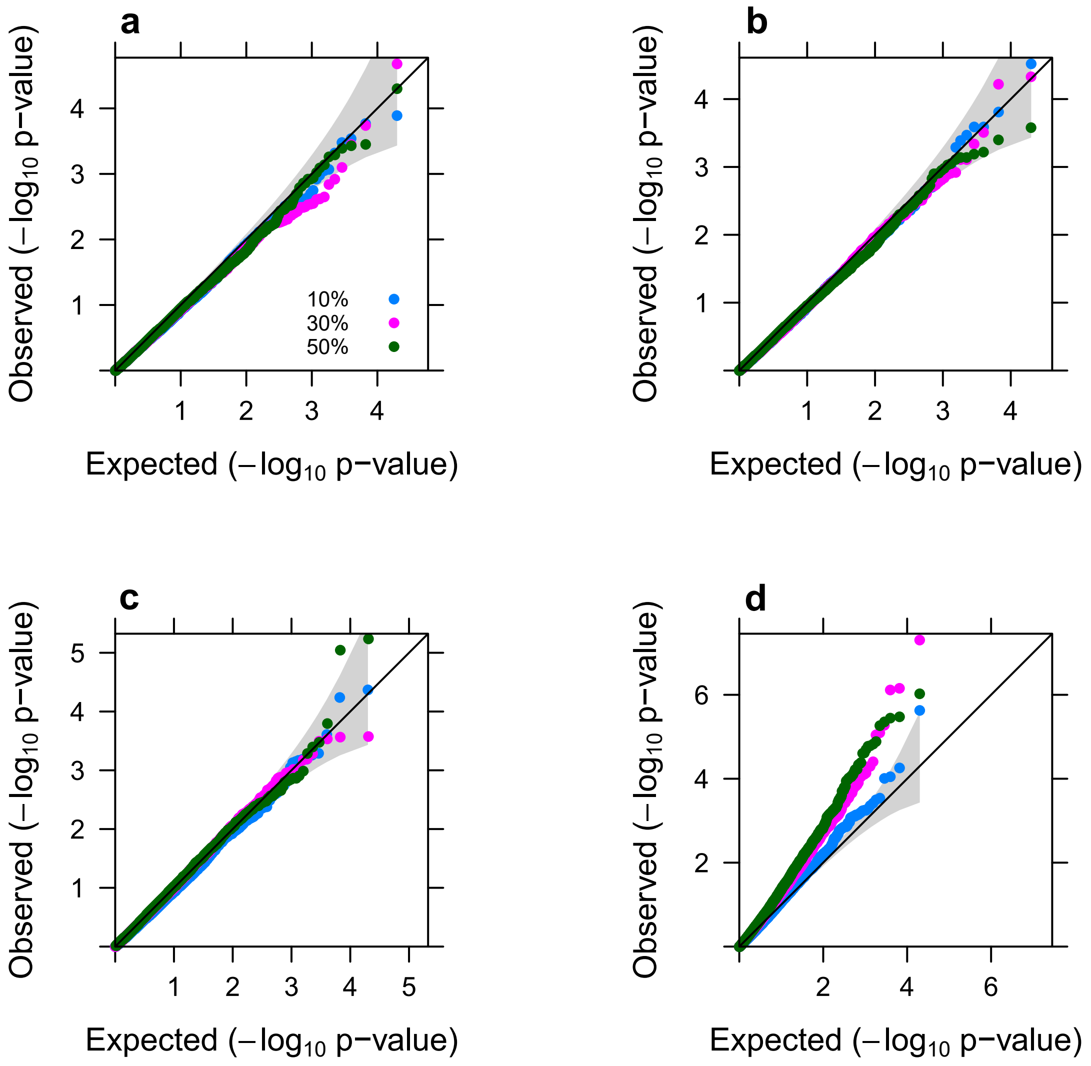

Supplementary Figure 12. Quantile-quantile plot of -log10 *p*-values from PMR-Egger under cross-gene simulations for testing the causal effect under the null, under various directional horizontal pleiotropic effect assumptions. Simulations are performed under different horizontal pleiotropic effect sizes: (**a**) γ=0.0001; (**b**) γ=0.0005; (**c**) γ=0.001; (**d**) γ=0.002. In each panel, a fixed proportion of SNPs (10%, 30%, or 50%) have positive horizontal pleiotropic effects while the remaining proportion of SNPs have negative horizontal pleiotropic effects. *P*-values from PMR-Egger behave reasonably well across a range of directional or balanced horizontal pleiotropic effect settings, except in the extreme case where horizontal pleiotropic effect size is very large (γ=0.002) and where the effect size signs across SNPs are approximately balanced.

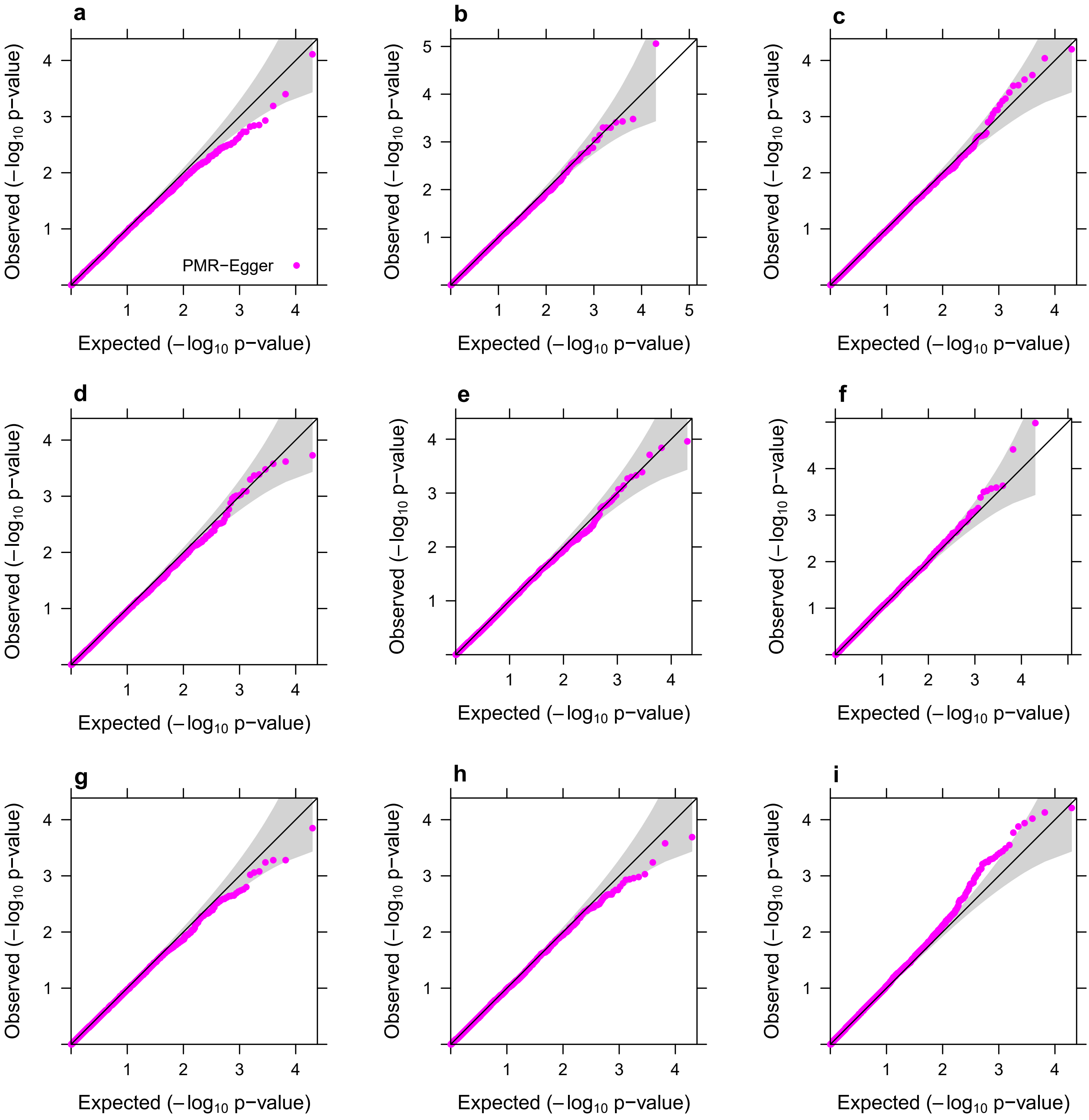

Supplementary Figure 13. Quantile-quantile plot of -log10 *p*-values from cross-gene simulations of PMR-Egger for testing the causal effect under null simulations, when randomly flipping the encoding at a fixed proportion of cis-SNPs, the proportion equals 10% (**a**, **b**, **c**), 30% (**d**, **e**, **f**) and 50% (**g**, **h**, **i**). Simulations are performed under different pleiotropy effect sizes, γ=0 (**a**, **d**, **g**), γ=0.0005 (**b**, **e**, **h**) or γ=0.001 (**c**, **f**, **i**). *P*-values from PMR-Egger behave reasonably well across most of the scenarios, and it is a little inflated in the case where the flipping proportion reached 50% and horizontal pleiotropic effect size is large (γ=0.001).

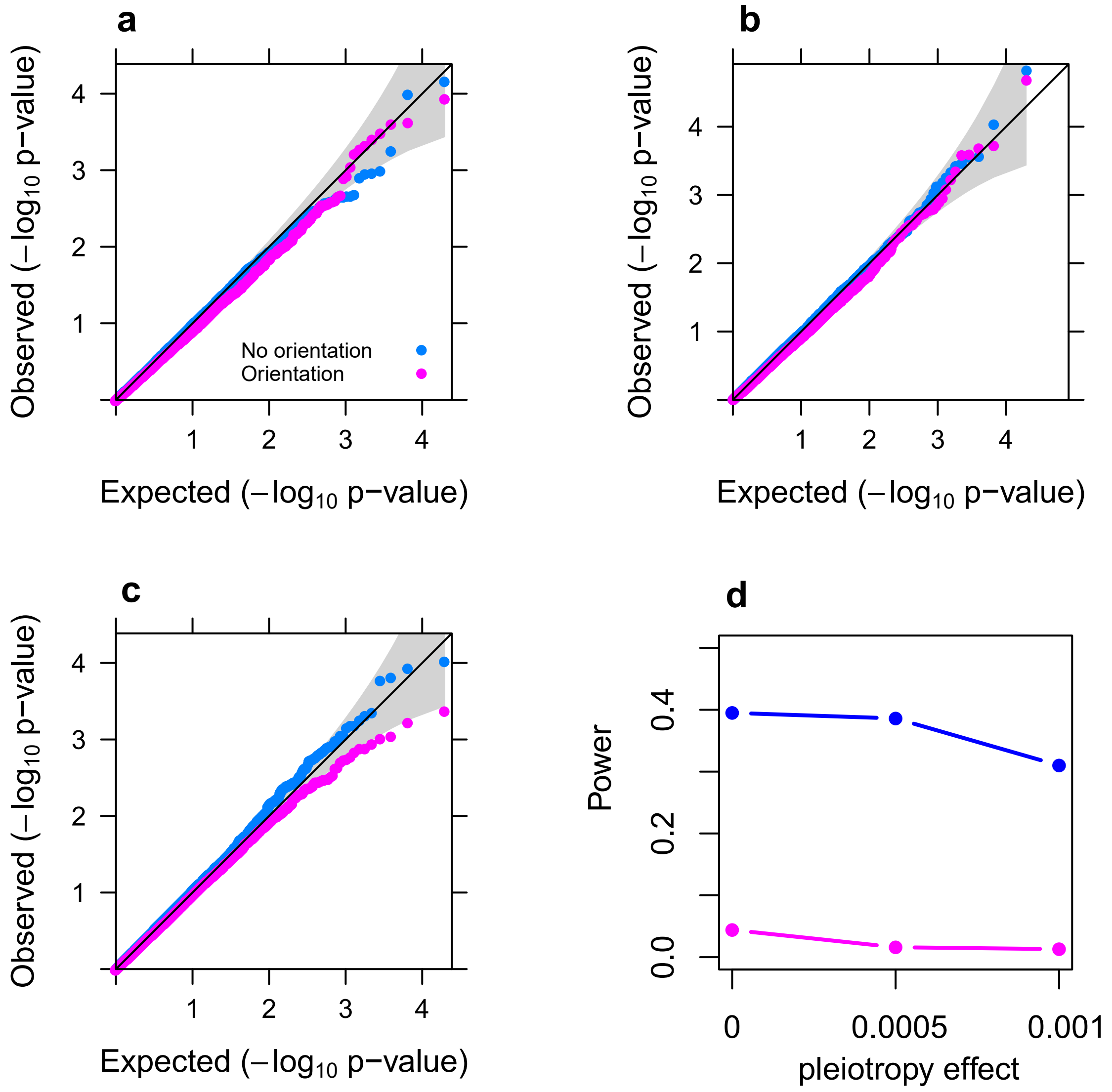

Supplementary Figure 14. Simulation results of PMR-Egger for causal effect test when randomly flipping the genotype encoding at 50% of cis-SNPs. The 'orientation' strategy is that genotypes are oriented so that the estimated SNP marginal effects of the gene expression are all positive. The *p*-values for testing the causal effect under the null are shown under different pleiotropy effect sizes, γ=0 (**a**), γ=0.0005 (**b**), γ=0.001 (**c**). Power (y-axis) at a false discovery rate of 0.1 to detect the causal effect (PVE_{zy}=0.6%) is plotted against different horizontal pleiotropy effect size (**d**).

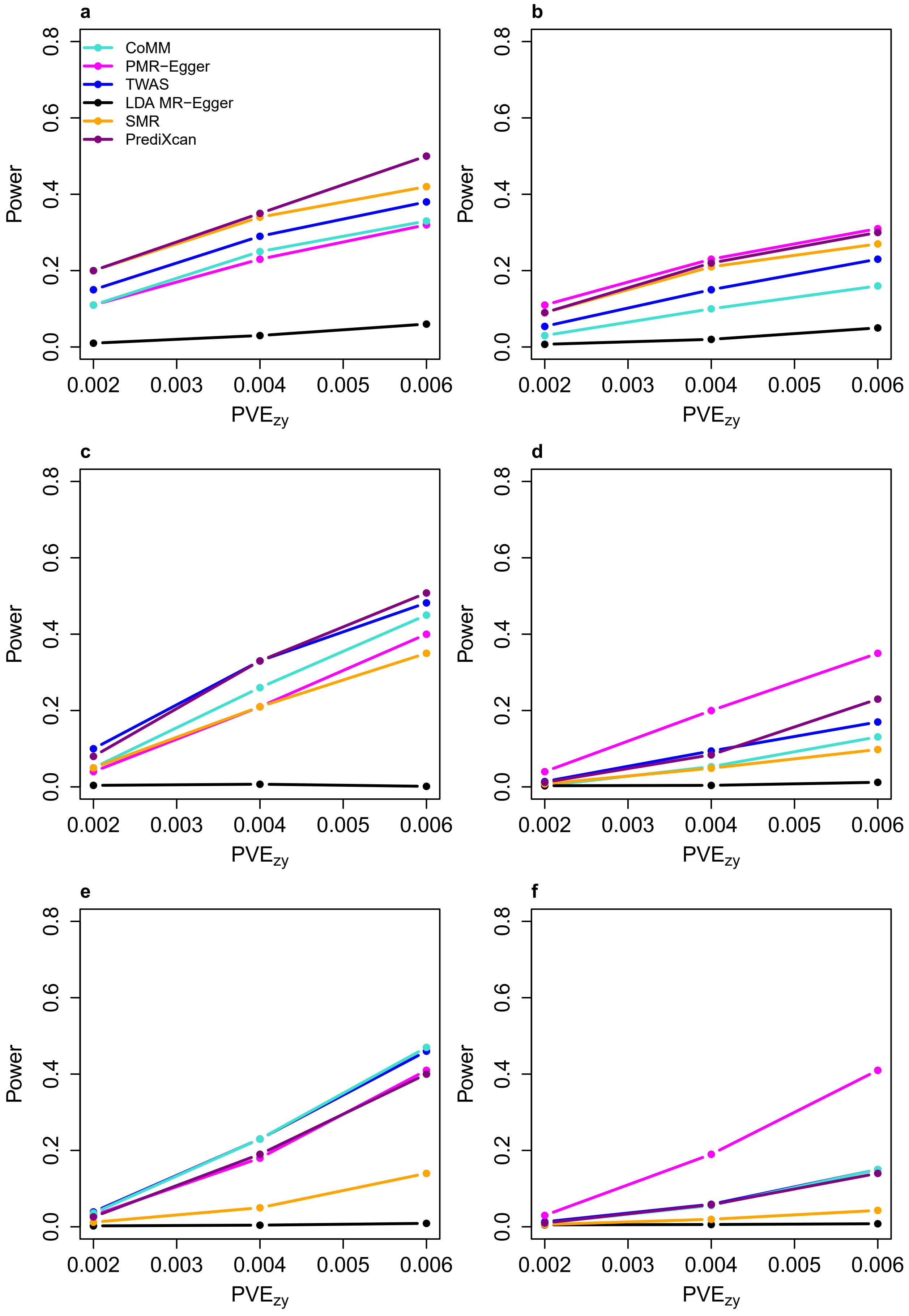

Supplementary Figure 15. Power for testing causal effect by different methods in various sparse settings where only a small proportion of SNPs are associated with the gene expression level. Power (y-axis) at a false discovery rate of 0.1 to detect the causal effect is plotted against different causal effect size characterized by PVE_{zy} (x-axis). Compared methods include CoMM (turquoise), PMR-Egger (magenta), TWAS (blue), LDA MR-Egger (black), SMR (orange), and PrediXcan (purple). Simulations are performed either in the absence (γ=0; **a**, **c**, **e**) or in the presence of horizontal pleiotropic effect (γ=0.001; **b**, **d**, **f**). Either one SNP (**a**, **b**), 1% of SNPs (**c**, **d**), or 10% SNPs (**e**, **f**) have non-zero effects on gene expression.

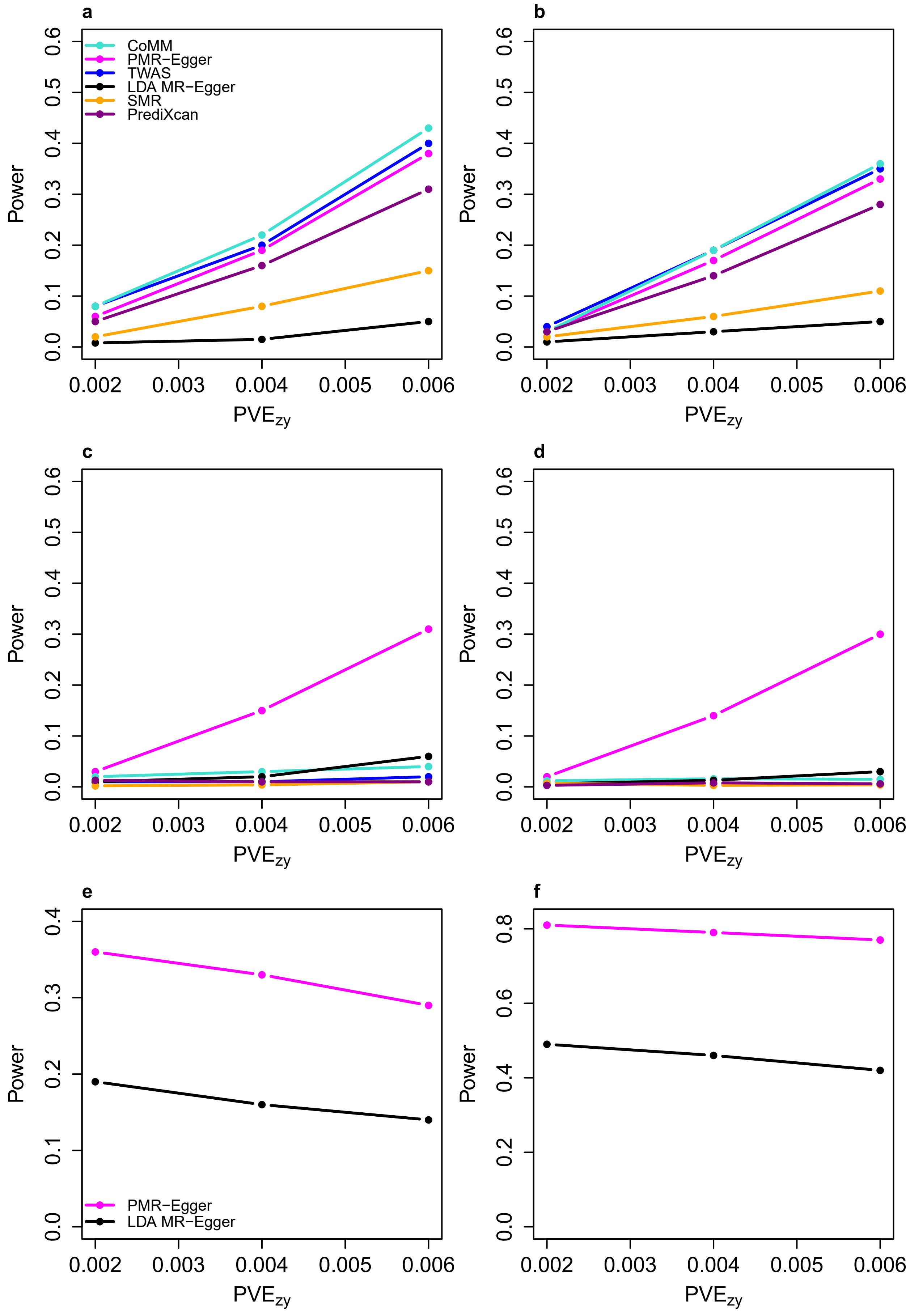

Supplementary Figure 16. Power of different methods under various cross-gene simulation scenarios. Power (y-axis) at a false discovery rate of 0.1 to detect the causal effect (**a-d**) or the horizontal pleiotropic effect (**e-f**) is plotted against different causal effect size characterized by PVE_{zy} (x-axis). Compared methods include CoMM (turquoise), PMR-Egger (magenta), TWAS (blue), LDA MR-Egger (black), SMR (orange), and PrediXcan (purple). Simulations are performed under different horizontal pleiotropic effect sizes: (**a**) γ=0; (**b**) γ=0.0001; (**c**, **e**) γ=0.0005; (**d**, **f**) γ=0.001.

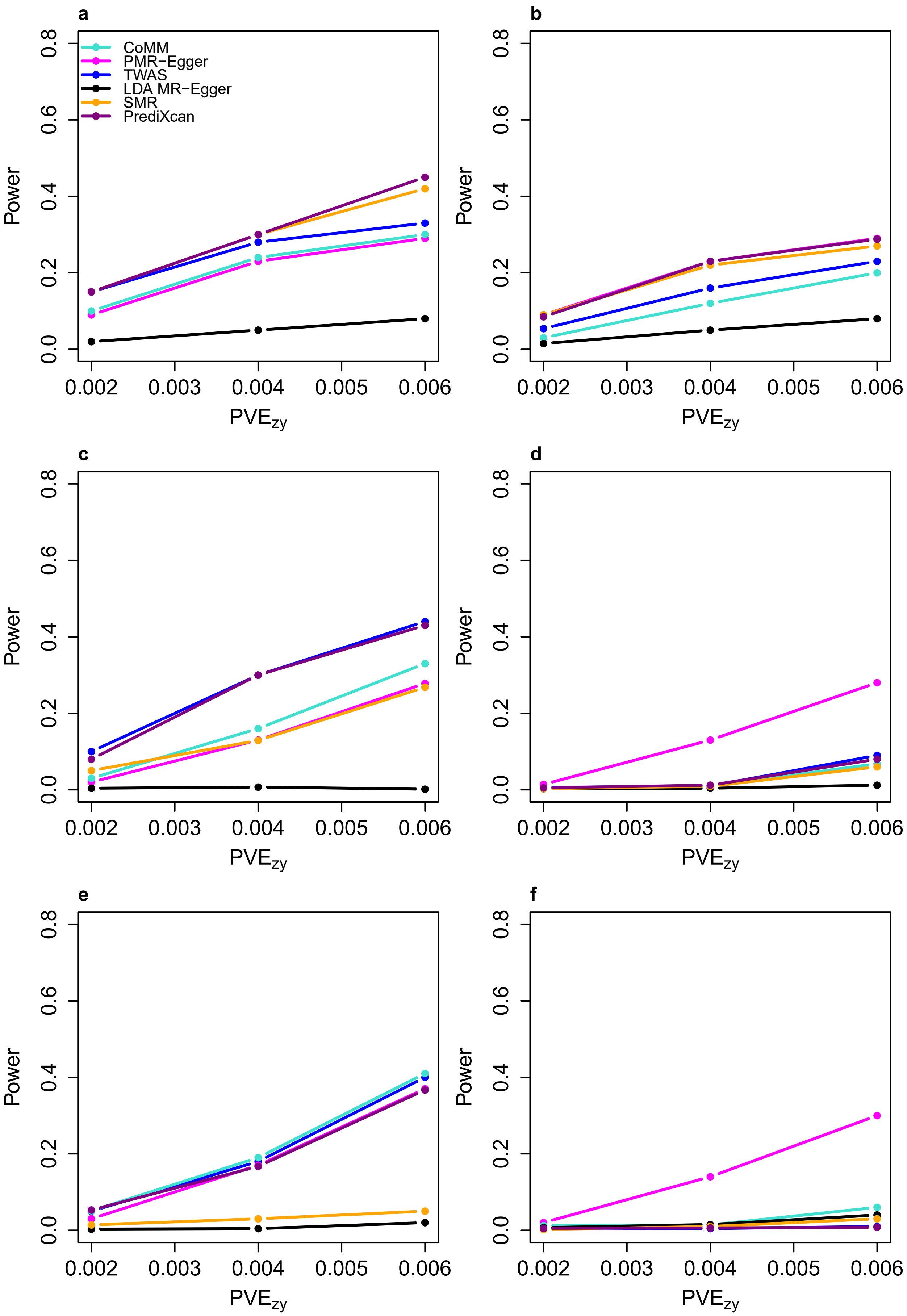

Supplementary Figure 17. Power for testing causal effect by different methods under various cross-gene simulation sparse settings where only a small proportion of SNPs are associated with the gene expression level. Power (y-axis) at a false discovery rate of 0.1 to detect the causal effect is plotted against different causal effect size characterized by PVE_{zy} (x-axis). Compared methods include CoMM (turquoise), PMR-Egger (magenta), TWAS (blue), LDA MR-Egger (black), SMR (orange), and PrediXcan (purple). Simulations are performed either in the absence (γ=0; **a**, **c**, **e**) or in the presence of horizontal pleiotropic effect (γ=0.001; **b**, **d**, **f**). Either one SNP (**a**, **b**), 1% of SNPs (**c**, **d**), or 10% SNPs (**e**, **f**) have non-zero effects on gene expression.

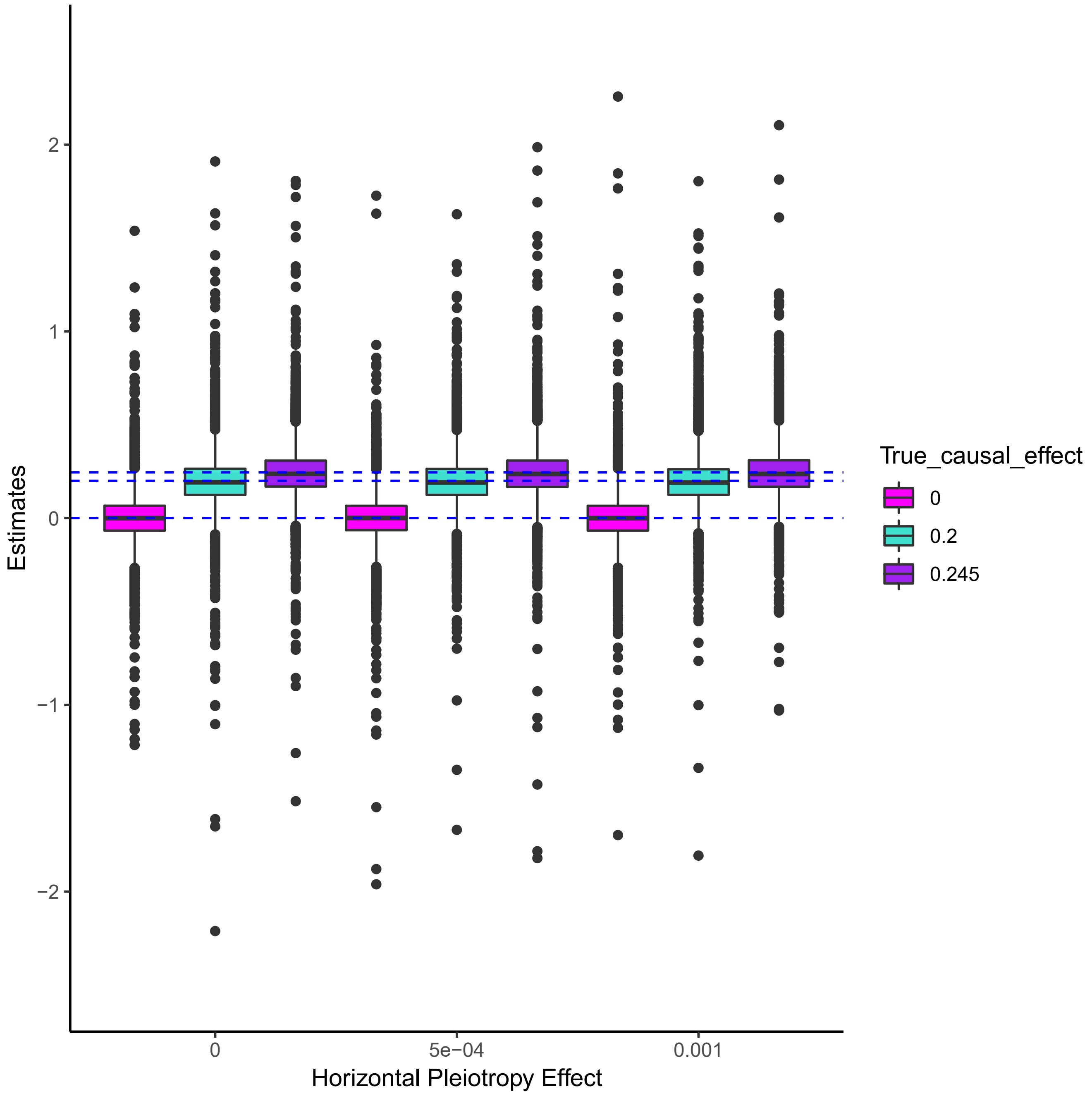

Supplementary Figure 18. Boxplot displays causal effect estimates by PMR-Egger in the absence or presence of horizontal pleiotropic effect. Simulations are performed under different horizontal pleiotropic effect sizes (x-axis: γ=0, γ=0.0005 or γ=0.001). For each horizontal pleiotropic effect size, we examined three true causal effect sizes α=0 (magenta), 0.2 (turquoise), or 0.245 (purple), which corresponds to PVE_{zy}=0, 0.4% and 0.6%, respectively. The horizontal blue dashed lines represent the three true values of α. The box is drawn from the first and third quantile (25th and 75th percentile) with a horizontal line drawn in the middle to denote the median. The lowest point is the minimum and the highest point is the maximum. PMR-Egger produces approximately unbiased causal effect size estimates across different scenarios. 10000 replicates are included for each simulation scenario.

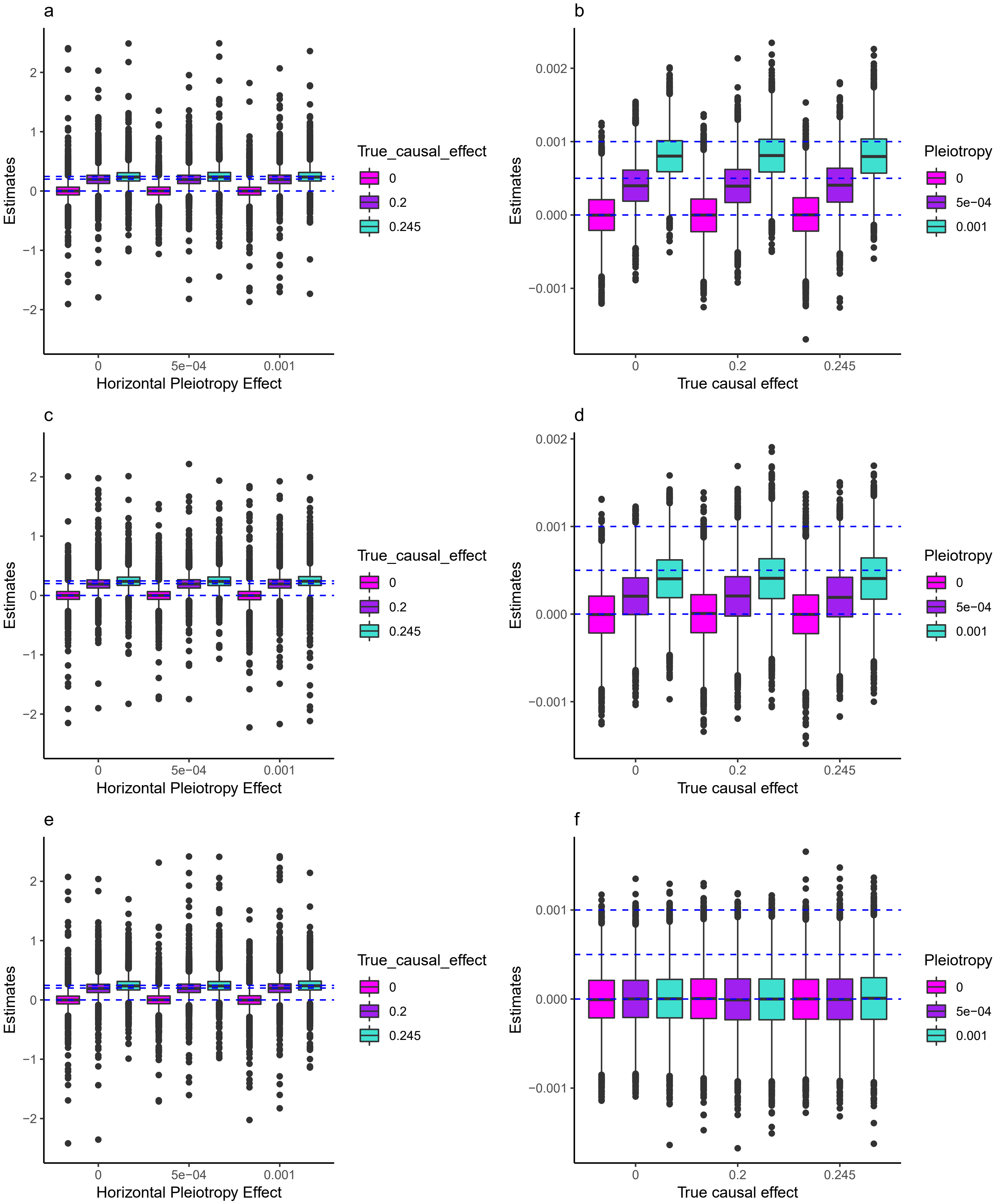

Supplementary Figure 19. Boxplot displays causal and horizontal pleiotropic effect estimates in cross-gene simulations by PMR-Egger when randomly flipping the encoding at a fixed proportion of cis-SNPs, the proportion equals 10% (**a**, **b**), 30% (**c**, **d**) and 50% (**e**, **f**). Simulations are performed under different causal effect sizes (α=0 (PVE_{zy}=0), α=0.2(PVE_{zy}=0.4%) or α=0.245 (PVE_{zy}=0.6%)) and pleiotropy effect sizes (γ=0, γ=0.0005 or γ=0.001). The horizontal blue dashed lines represent the three true values of α (**a**, **c**, **e**) and γ (**b**, **d**, **f**). In the boxplot, each box is drawn from the first and third quantile (25th and 75th percentile) with a horizontal line drawn in the middle to denote the median. The lowest point is the minimum and the highest point is the maximum. 10,000 replicates are included for each simulation scenario. The causal effect estimates are always unbiased regardless of the pleiotropy effect size and the proportion of flipping coding. When there is no pleiotropy effect (γ=0), the estimation of pleiotropy effect is always unbiased regardless of the causal effect size and the proportion of flipping encoding. However, in the presence of pleiotropy effect (γ=0.0005,0.001), PMR-Egger seems to underestimate the pleiotropy effect, and more so with the increasing proportion of flipping encoding. As expected, when the proportion of flipping encoding is 50%, the estimation of pleiotropy effect is always close to 0. Furthermore, the strength of underestimation seems unrelated with the causal effect size.

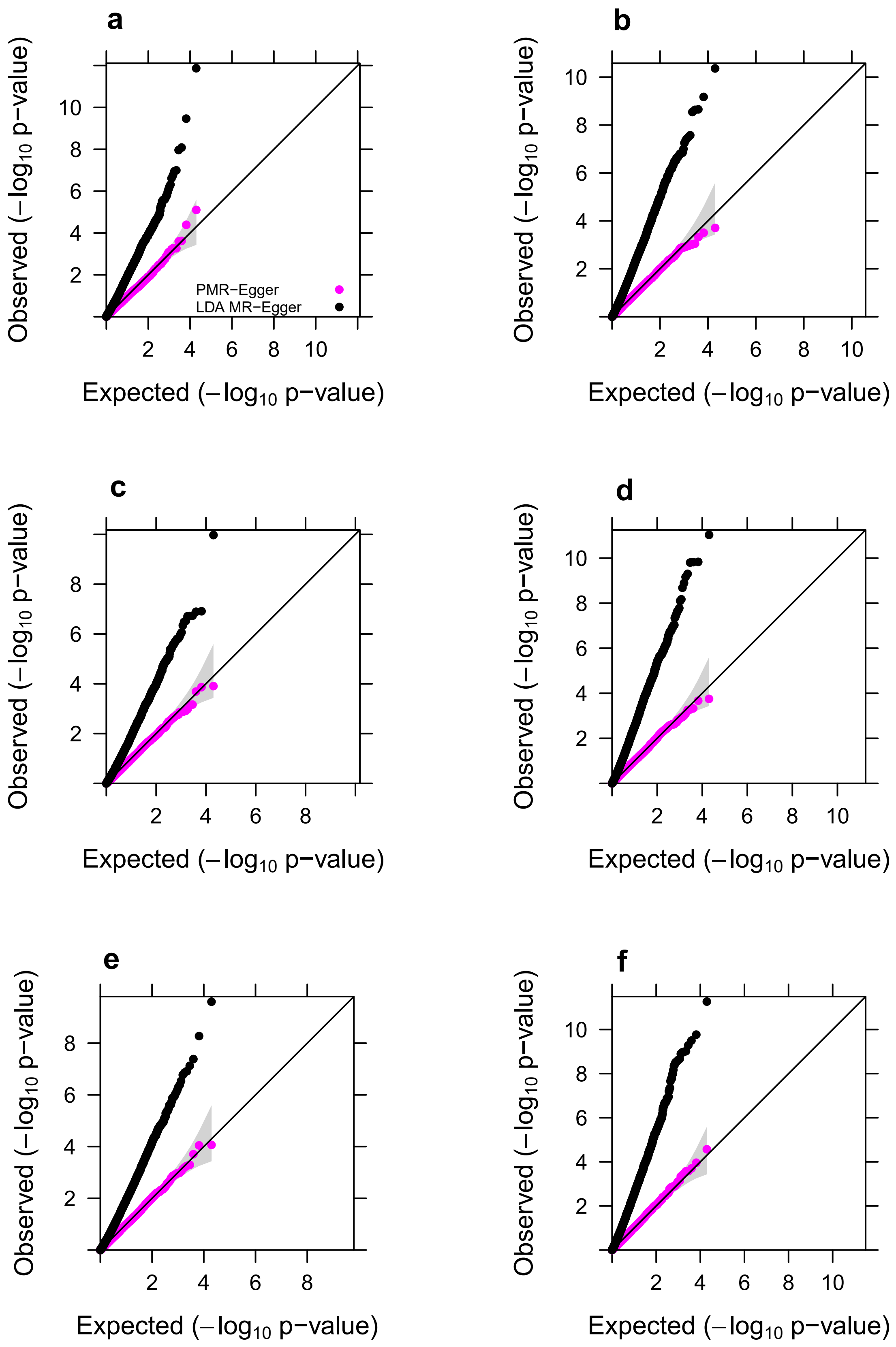

Supplementary Figure 20. Quantile-quantile plot of -log10 *p*-values from different methods for testing the horizontal pleiotropic effect under null simulations, in various sparse settings where only a small proportion of SNPs are associated with the gene expression level. Compared methods include PMR-Egger (magenta) and LDA MR-Egger (black). Simulations are performed either in the absence (PVE_{zy}=0; **a**, **c**, **e**) or in the presence of causal effect (PVE_{zy}=0.6%; **b**, **d**, **f**). Either one SNP (**a**, **b**), 1% of SNPs (**c**, **d**), or 10% SNPs (**e**, **f**) have non-zero effects on gene expression. Only *p*-values from PMR-Egger adhere to the expected diagonal line across a range of settings. Note that we do not include MR-PRESSO into comparison here due to the relatively higher computation burden and it is difficult in MR-PRESSO to pre-specify the number of simulated expected distribution and obtain the exact *p*-values.

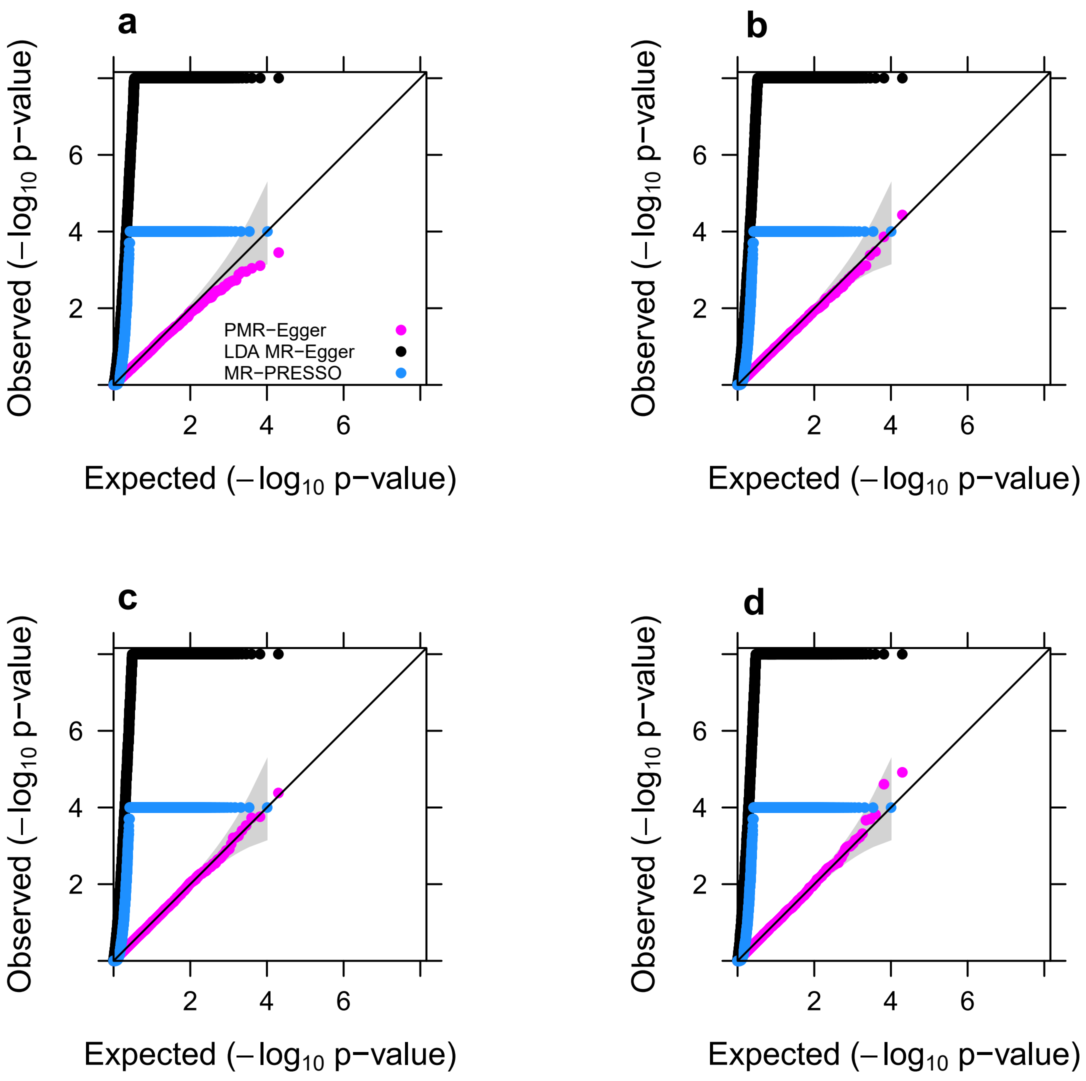

Supplementary Figure 21. Quantile-quantile plot of -log10 *p*-values from cross-gene simulations of different methods for testing the horizontal pleiotropic effect either in the absence or in the presence of causal effect under null simulations. Compared methods include PMR-Egger (magenta), LDA MR-Egger (black), and MR-PRESSO (dodger blue). Null simulations are performed under different causal effect sizes characterized by PVE_{zy}: (**a**) PVE_{zy}=0; (**b**) PVE_{zy}=0.2%; (**c**) PVE_{zy}=0.4%; and (**d**) PVE_{zy}=0.6%. Only *p*-values from PMR-Egger adhere to the expected diagonal line across a range of horizontal pleiotropic effect sizes. Due to heavy computational burden, we are only able to run 10,000 permutations for MR-PRESSO. Therefore, the minimal *p*-value from MR-PRESSO is 10^{-4}.

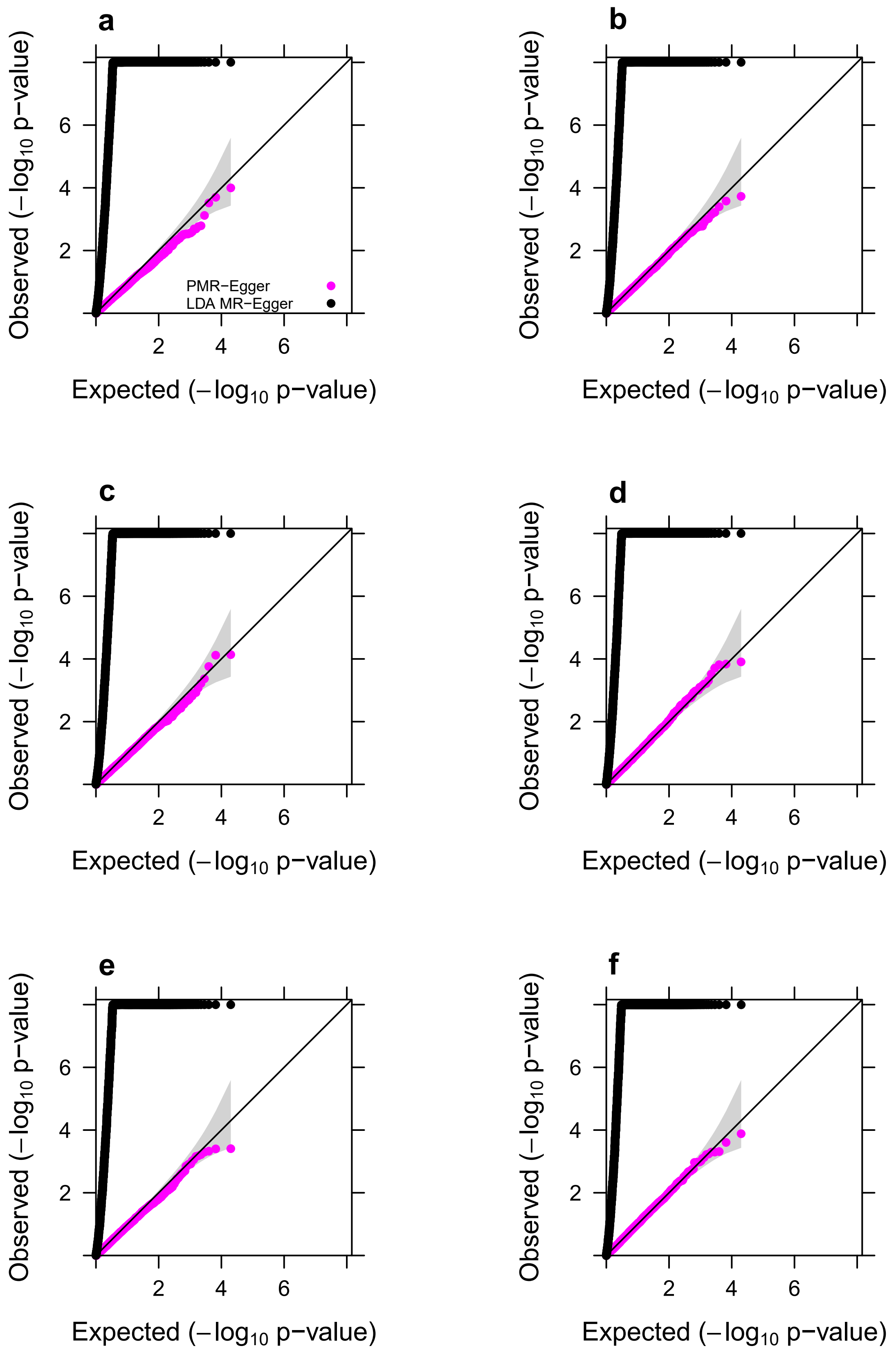

Supplementary Figure 22. Quantile-quantile plot of -log10 *p*-values from cross-gene simulations of different methods for testing the horizontal pleiotropic effect under null simulations, in various sparse settings where only a small proportion of SNPs are associated with the gene expression level. Compared methods include PMR-Egger (magenta) and LDA MR-Egger (black). Simulations are performed either in the absence (PVE_{zy}=0; **a**, **c**, **e**) or in the presence of causal effect (PVE_{zy}=0.6%; **b**, **d**, **f**). Either one SNP (**a**, **b**), 1% of SNPs (**c**, **d**), or 10% SNPs (**e**, **f**) have non-zero effects on gene expression. Only *p*-values from PMR-Egger adhere to the expected diagonal line across a range of settings. Note that we do not include MR-PRESSO into comparison here due to the relatively higher computation burden and it is difficult in MR-PRESSO to pre-specify the number of simulated expected distribution and obtain the exact *p*-values.

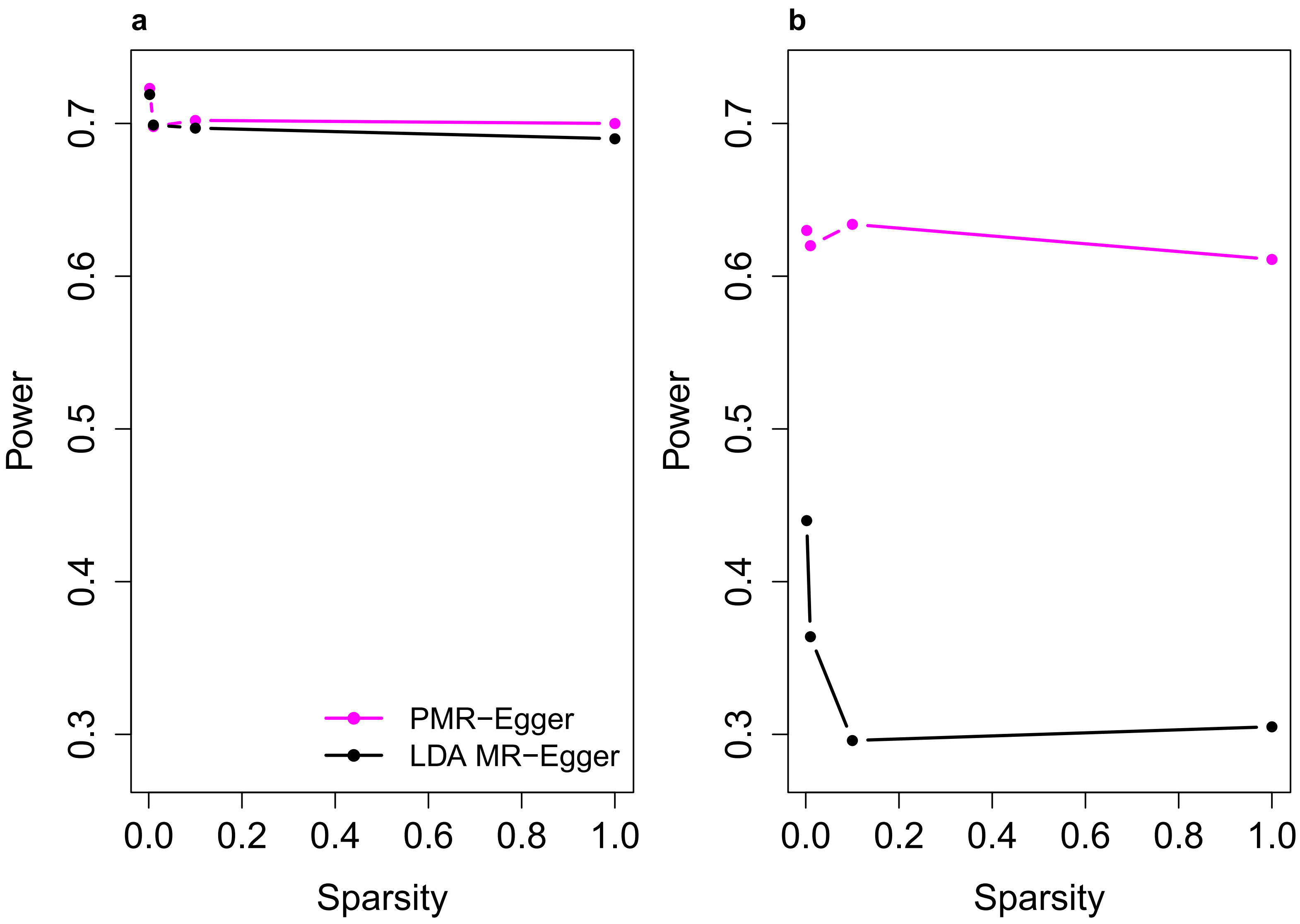

Supplementary Figure 23. Power of different methods for identifying horizontal pleiotropic effect, in various sparse settings where only a small proportion of SNPs are associated with the gene expression level. Compared methods include PMR-Egger (magenta) and LDA MR-Egger (black). Power (y-axis) at a false discovery rate of 0.1 to detect the horizontal pleiotropic effect is plotted against sparsity levels, either in the absence (PVE_{zy}=0; **a**) or in the presence of causal effect (PVE_{zy}=0.6%; **b**). In terms of sparsity level (x-axis), either one SNP, 1% SNPs, 10% SNPs and 100% SNPs have non-zero effects on gene expression.

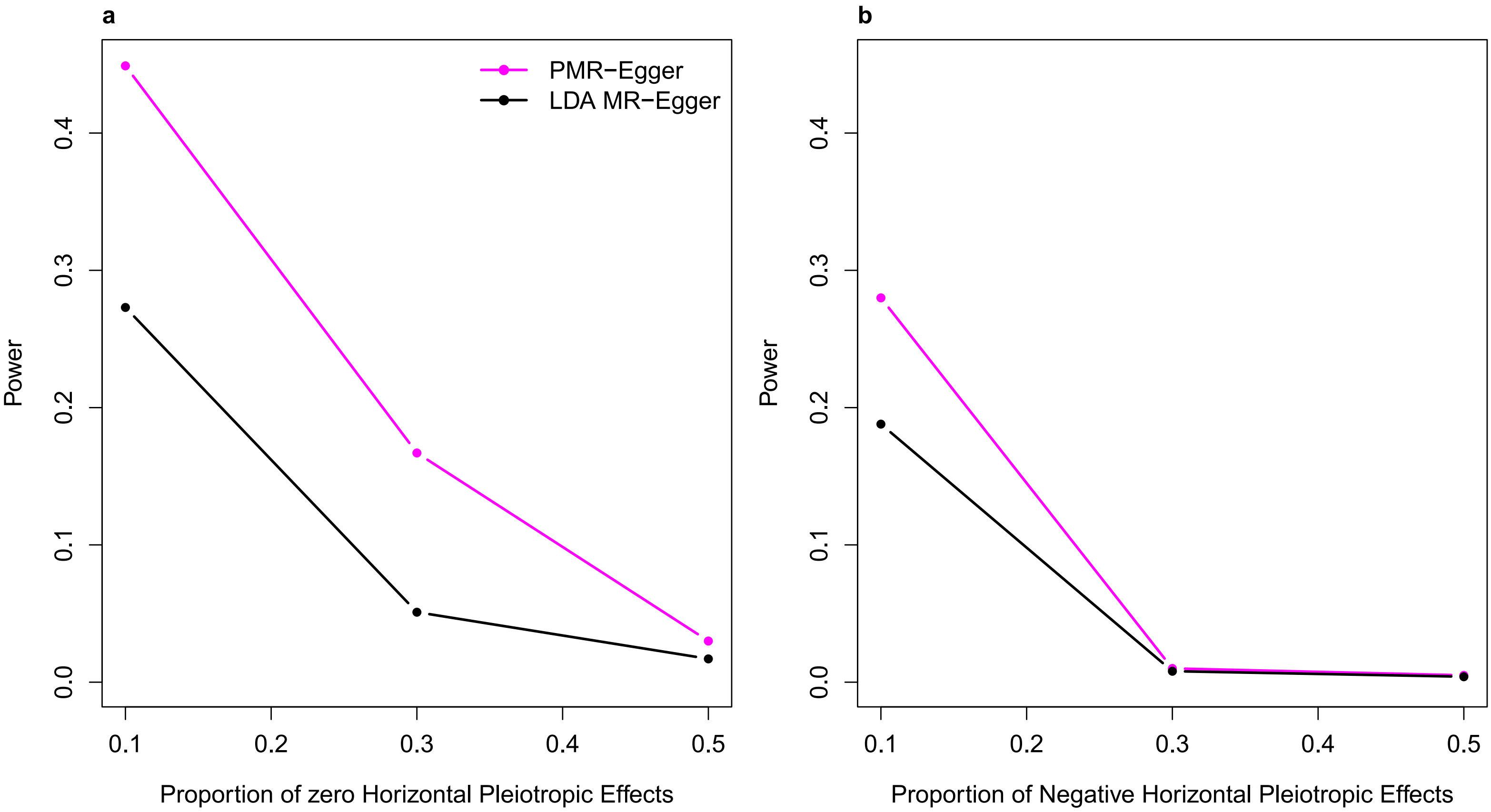

Supplementary Figure 24. Power of different methods for identifying horizontal pleiotropic effect under various model misspecifications of the horizontal pleiotropic effect. Compared methods include PMR-Egger (magenta) and LDA MR-Egger (black). On both panels, power (y-axis) to detect the horizontal pleiotropic effect is measured at a false discovery rate of 0.1 for a fixed horizontal pleiotropic effect size γ=0.001. (**a**) Power is plotted against the proportion of SNPs displaying zero horizontal pleiotropy effects (10%, 30%, or 50%). (**b**) Power is plotted against the proportion of SNPs exhibiting negative horizontal pleiotropy effects (10%, 30%, or 50%), whereas the remaining proportion of SNPs exhibiting positive effects.

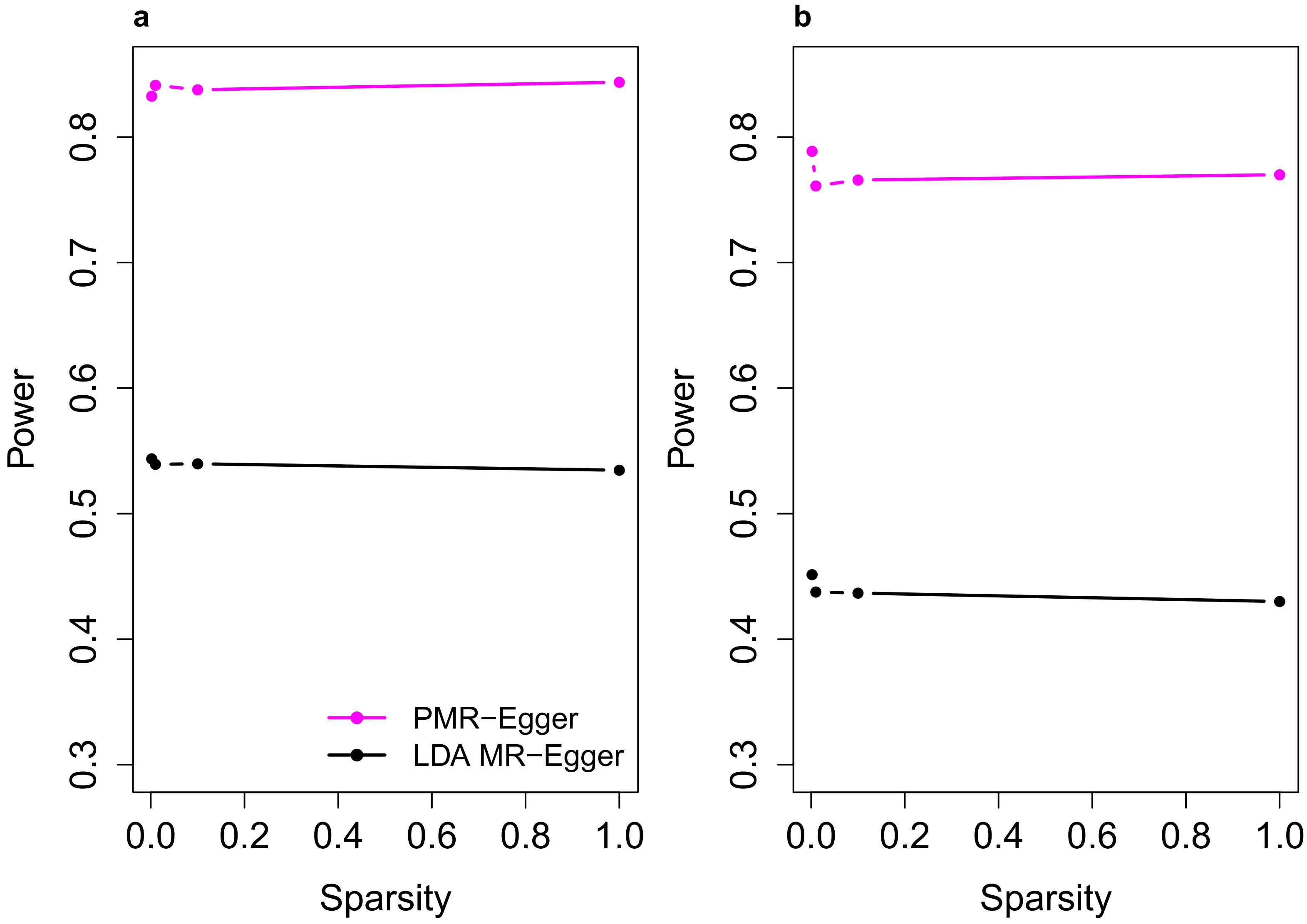

Supplementary Figure 25. Power of different methods for identifying horizontal pleiotropic effect, in various cross-gene simulation sparse settings where only a small proportion of SNPs are associated with the gene expression level. Compared methods include PMR-Egger (magenta) and LDA MR-Egger (black). Power (y-axis) at a false discovery rate of 0.1 to detect the horizontal pleiotropic effect is plotted against sparsity levels, either in the absence (PVE_{zy}=0; **a**) or in the presence of causal effect (PVE_{zy}=0.6%; **b**). In terms of sparsity level (x-axis), either one SNP, 1% SNPs, 10% SNPs and 100% SNPs have non-zero effects on gene expression.

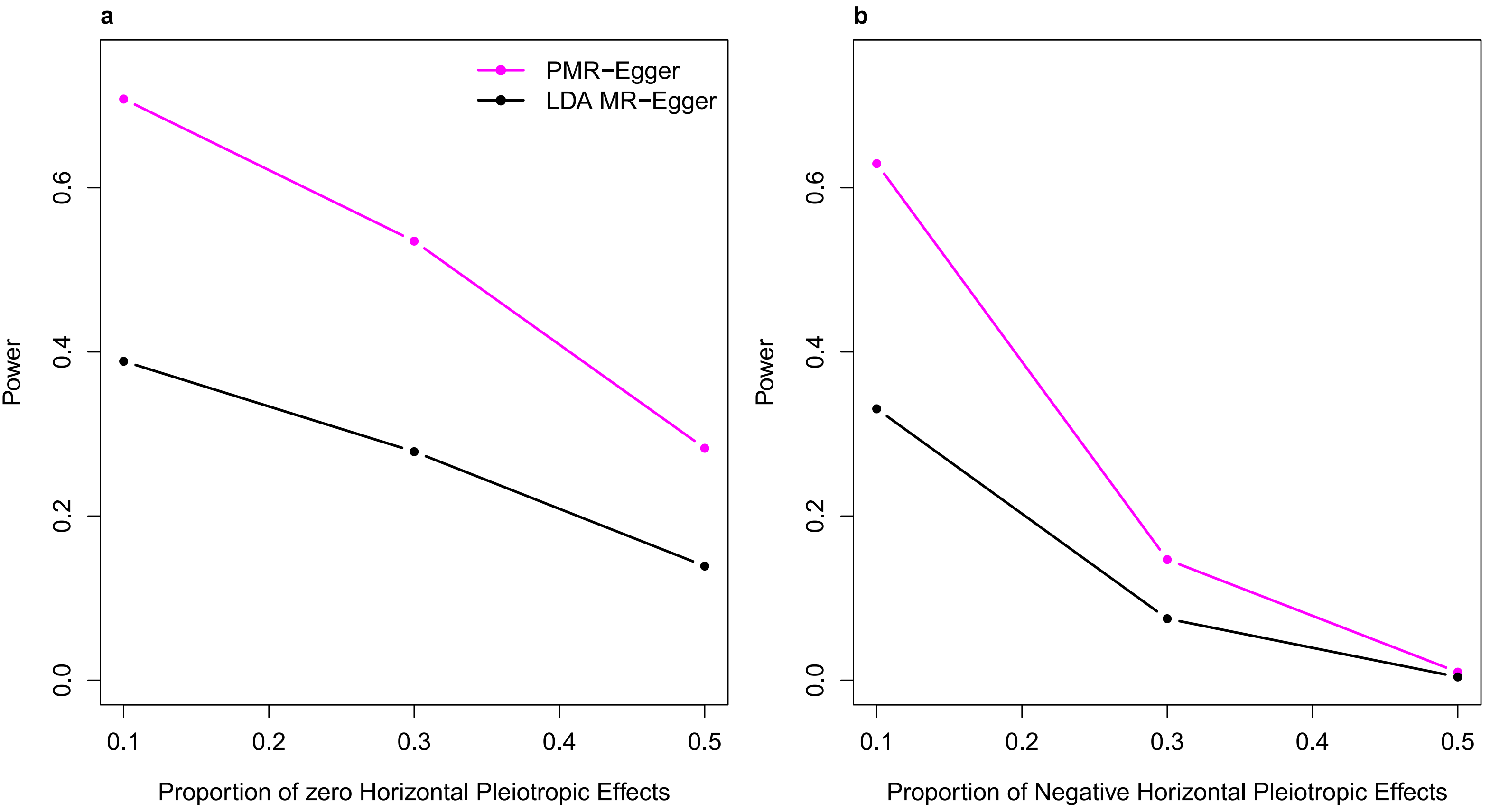

Supplementary Figure 26. Power of different methods for identifying horizontal pleiotropic effect in cross-gene simulations under various model misspecifications of the horizontal pleiotropic effect. Compared methods include PMR-Egger (magenta) and LDA MR-Egger (black). On both panels, power (y-axis) to detect the horizontal pleiotropic effect is measured at a false discovery rate of 0.1 for a fixed horizontal pleiotropic effect size γ=0.001. (**a**) Power is plotted against the proportion of SNPs displaying zero horizontal pleiotropy effects (10%, 30%, or 50%). (**b**) Power is plotted against the proportion of SNPs exhibiting negative horizontal pleiotropy effects (10%, 30%, or 50%), whereas the remaining proportion of SNPs exhibiting positive effects.

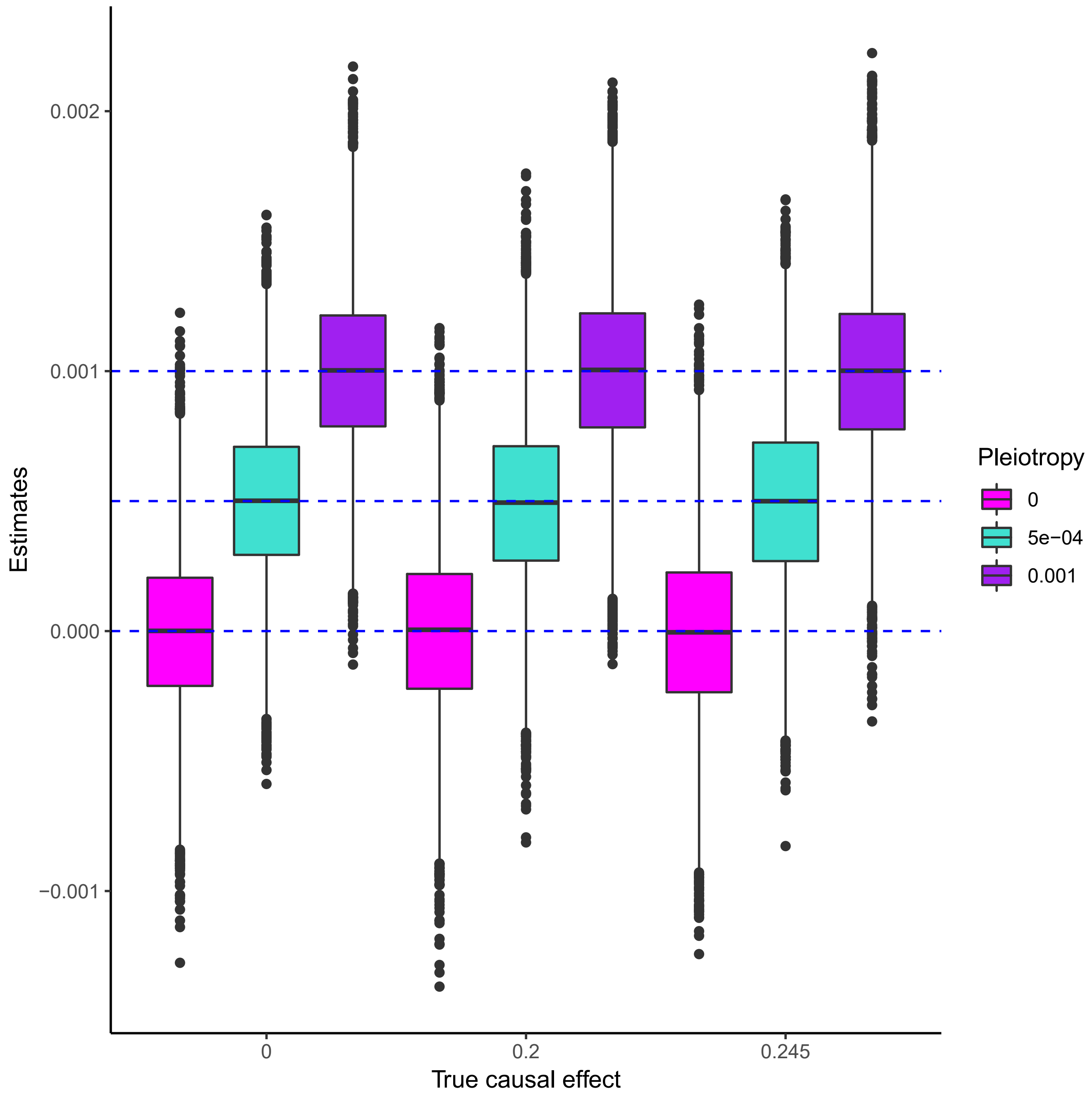

Supplementary Figure 27. Boxplot displays horizontal pleiotropic effect estimates by PMR-Egger in the absence or presence of causal effect. Simulations are performed under different causal effect sizes (x-axis: α=0 (PVE_{zy}=0), α=0.2 (PVE_{zy}=0.4%) or α=0.245 (PVE_{zy}=0.6%)). For each causal effect size, we examined three true horizontal pleiotropic effect sizes γ=0 (magenta), 0.0005 (turquoise), or 0.001 (purple). The horizontal blue dashed lines represent the three true values of γ. In the boxplot, each box is drawn from the first and third quantile (25th and 75th percentile) with a horizontal line drawn in the middle to denote the median. The lowest point is the minimum and the highest point is the maximum. PMR-Egger produces approximately unbiased pleiotropy effect size estimates across different scenarios. 10,000 replicates are included for each simulation scenario.

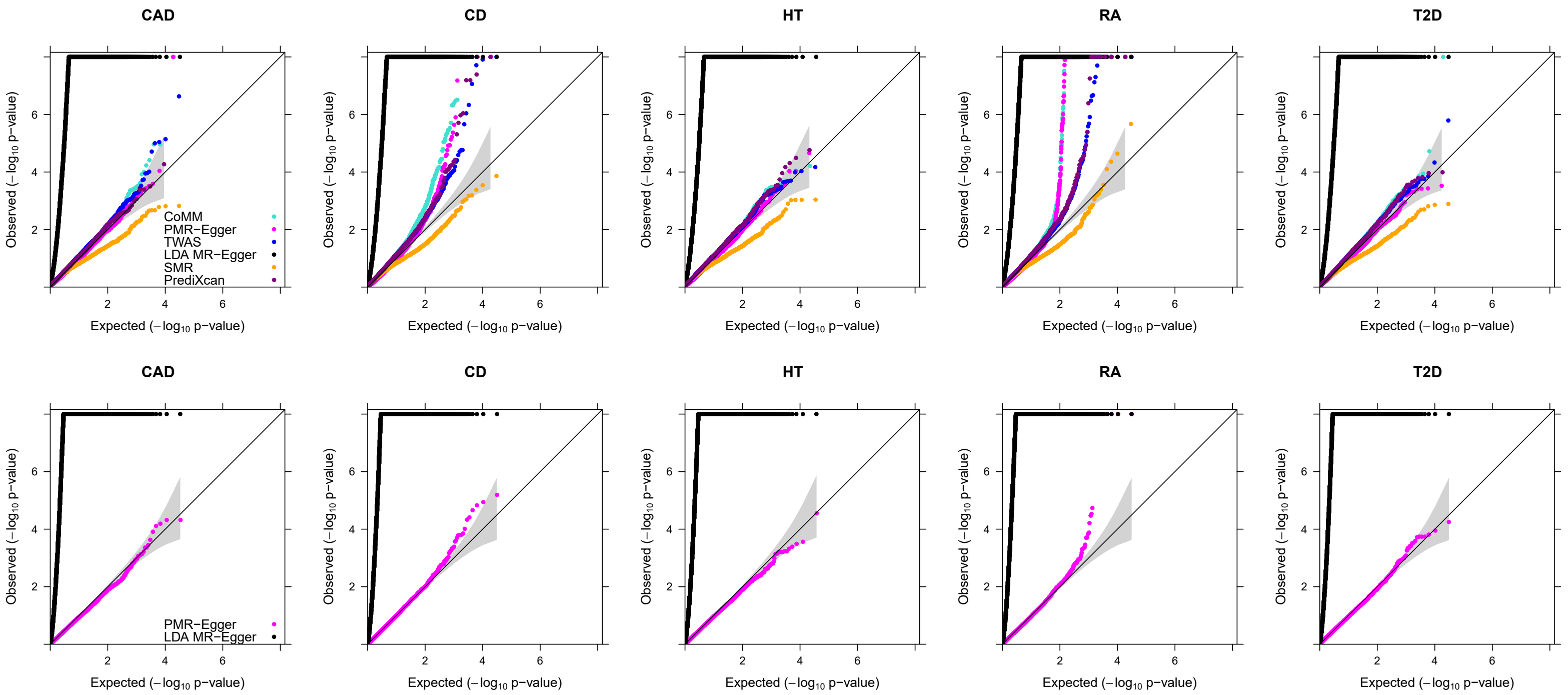

Supplementary Figure 28. Quantile-quantile plot of -log10 *p*-values from different methods in the TWAS application to WTCCC. Compared methods include CoMM (turquoise), PMR-Egger (magenta), TWAS (blue), LDA MR-Egger (black), SMR (orange), and PrediXcan (purple). Top panels: Quantile-quantile plot of -log10 *p*-values from different methods for testing the causal effect for five traits (CAD, CD, HT, RA and T2D). Bottom panels: Quantile-quantile plot of -log10 *p*-values from different methods for testing the horizontal pleiotropic effect for the same five traits.

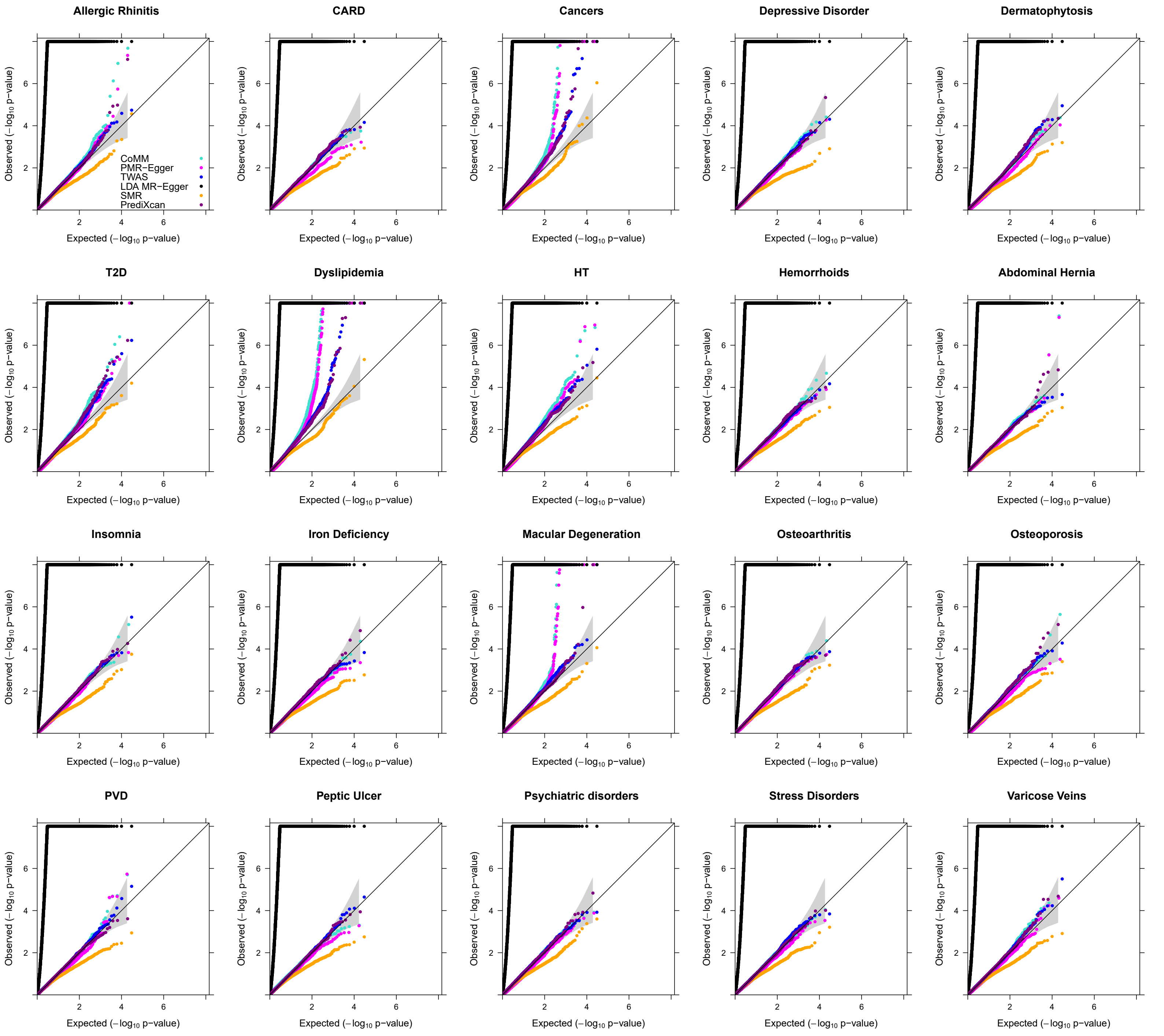

Supplementary Figure 29. Quantile-quantile plot of -log10 *p*-values from different methods for testing causal effect in the TWAS application to 20 traits in GERA. Compared methods include CoMM (turquoise), PMR-Egger (magenta), TWAS (blue), LDA MR-Egger (black), SMR (orange), and PrediXcan (purple).

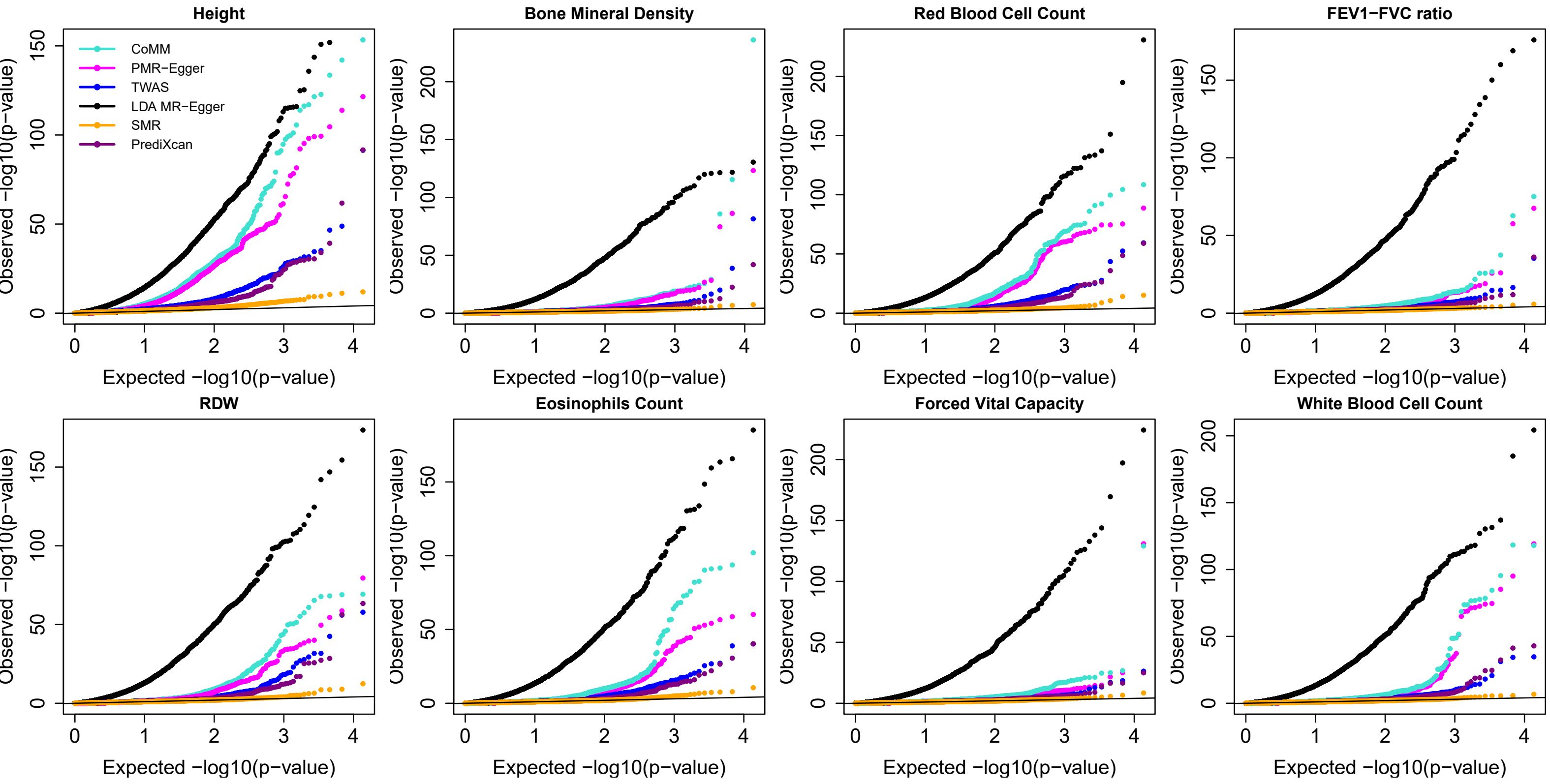

Supplementary Figure 30. Quantile-quantile plot of -log10 *p*-values from different methods for testing causal effect in the TWAS application to 8 traits in UK Biobank. Compared methods include CoMM (turquoise), PMR-Egger (magenta), TWAS (blue), LDA MR-Egger (black), SMR (orange), and PrediXcan (purple).

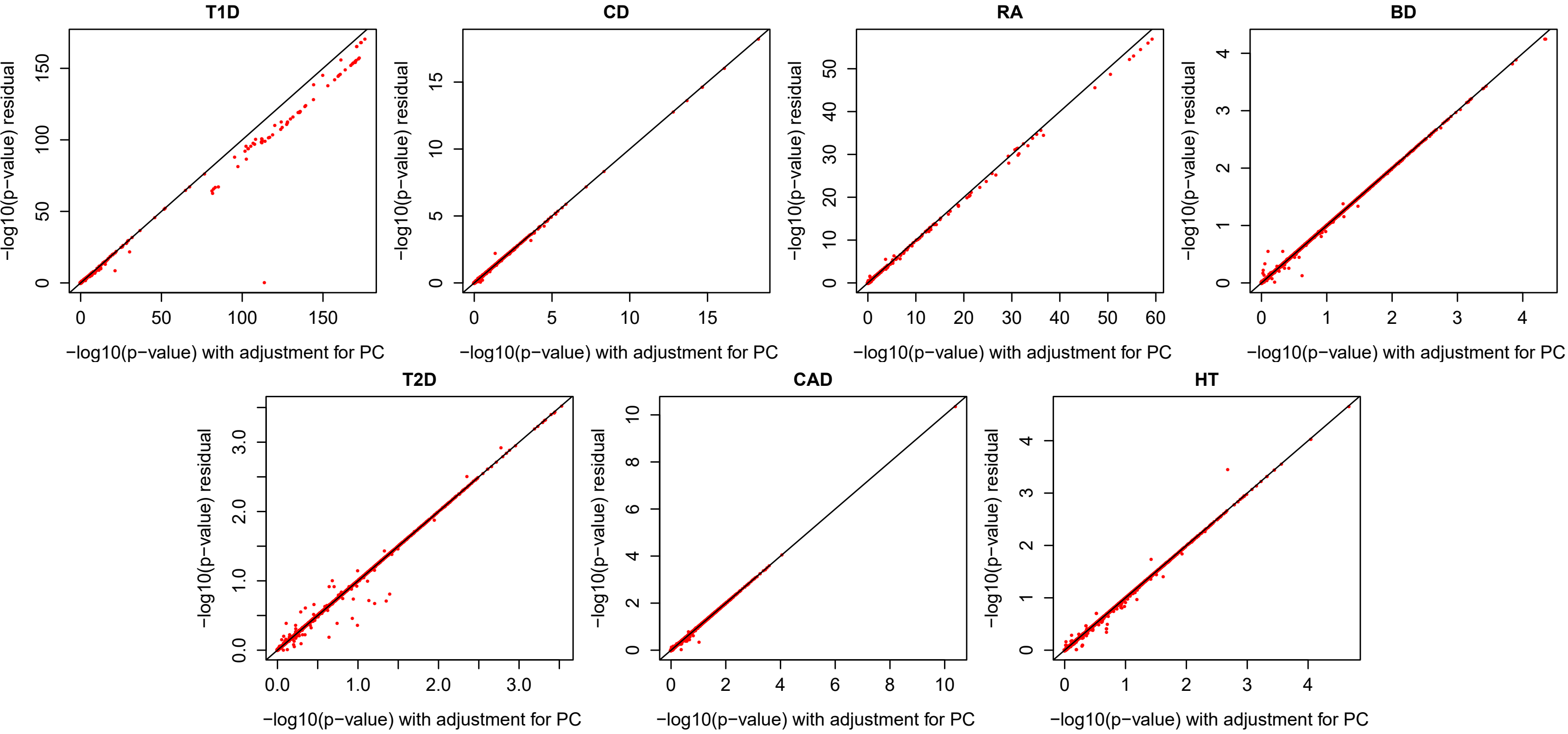

Supplementary Figure 31. The scatter plots of -log10 *p*-values across genes for the 7 WTCCC traits. The *p*-values from phenotype residual analysis (y-axis) is plotted against the *p*-values from analysis that treating the top 10 PCs as fixed covariates.

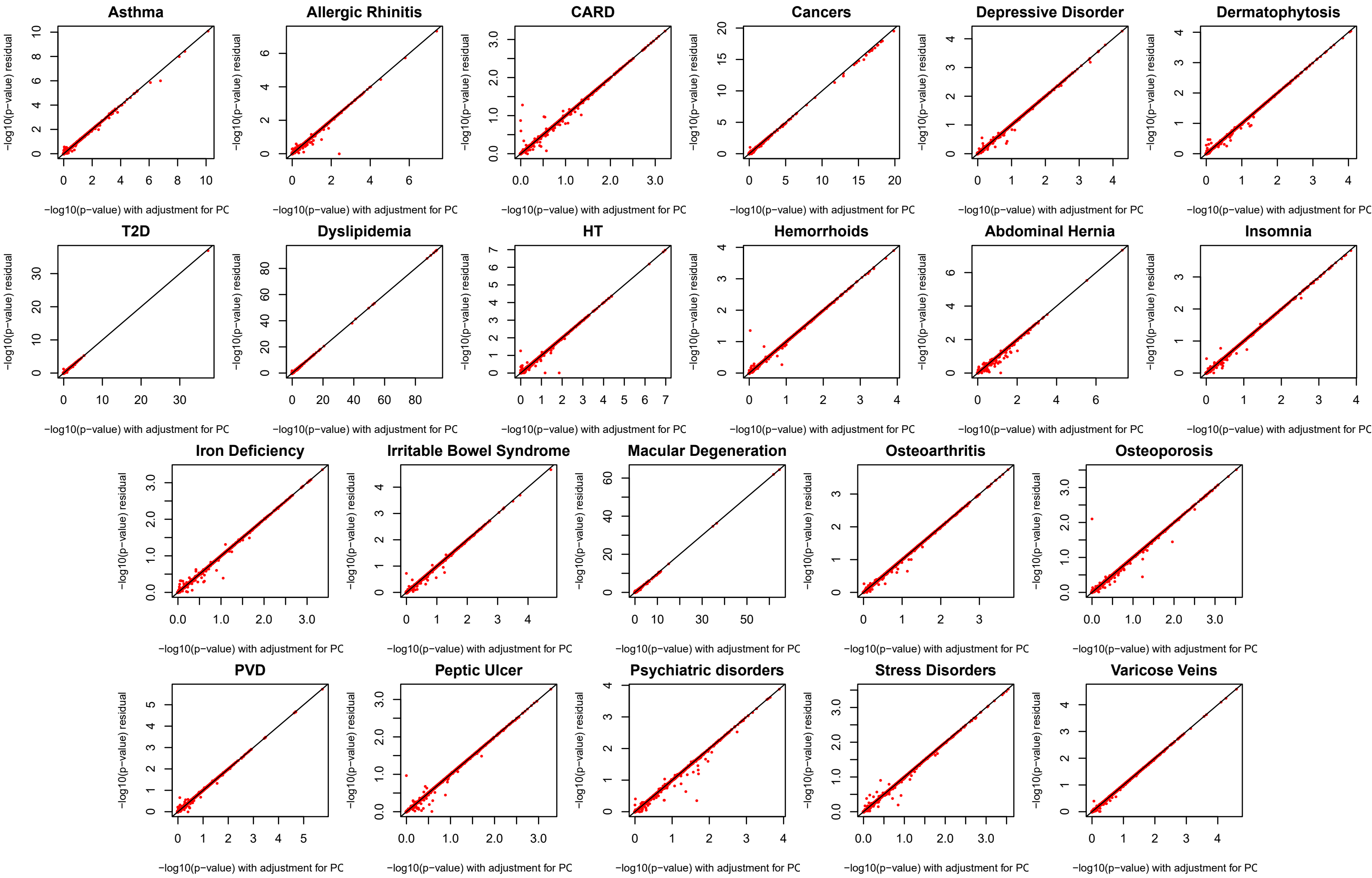

Supplementary Figure 32. The scatter plots of -log10 *p*-values across genes for the 22 GERA traits. The *p*-values from phenotype residual analysis (y-axis) is plotted against the *p*-values from analysis that treating the top 10 PCs as fixed covariates.

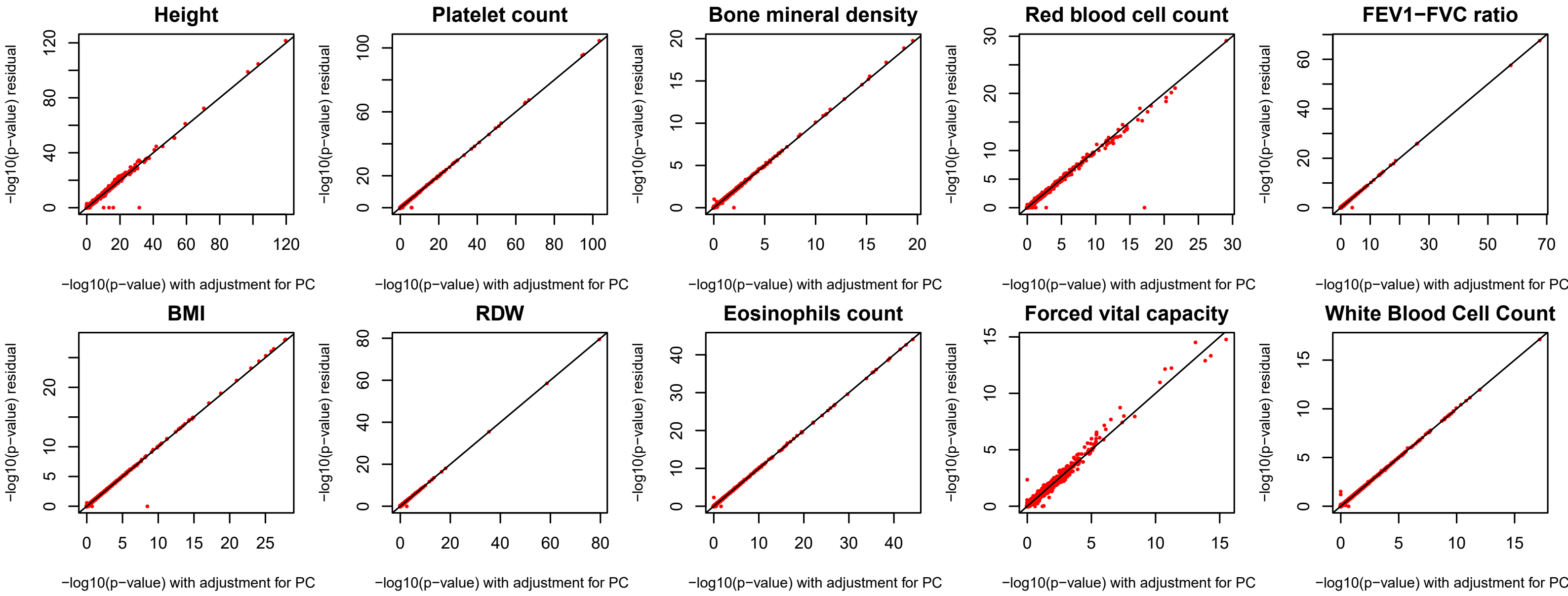

Supplementary Figure 33. The scatter plots of -log10 *p*-values across genes for the 10 UKBB traits. The *p*-values from phenotype residual analysis (y-axis) is plotted against the *p*-values from analysis that treating the top 10 PCs as fixed covariates. Note that we only used the genes locating at first 5 chromosomes (4977 total) given the relatively high computation burden for the large sample UKBB data.

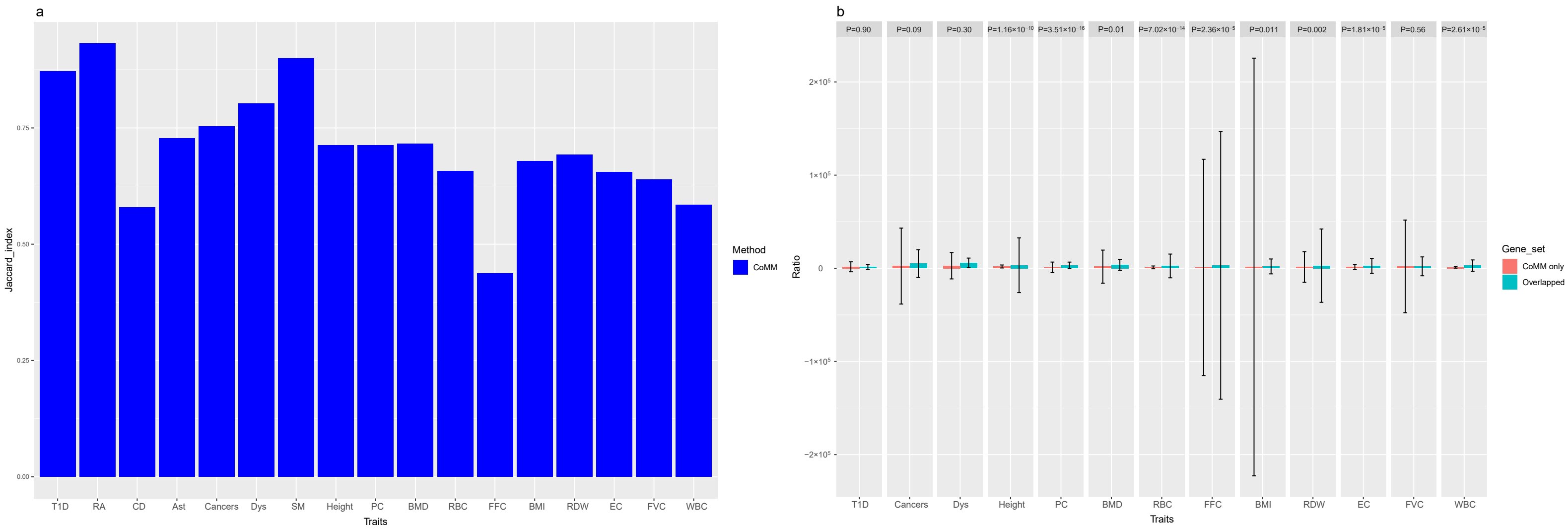

Supplementary Figure 34. Bar plots display overlap of genes detected by PMR-Egger and CoMM. (**a**) Jaccard index measures relatively high overlap between genes detected by PMR-Egger and genes detected by CoMM. (**b**) Mean of the estimated $|\alpha/\gamma|$ for the set of genes that are detected by both CoMM and PMR-Egger ("overlapped"), and for the set of genes that only detected by CoMM ("CoMM only"), across 13 traits that have at least 2 detected associations. In (**b**), data are presented as mean±standard error of the mean (SEM), with p-values calculated based on Wilcoxon rank sum test. The traits on x-axis are: Ast, Asthma; Dys, Dyslipidemia; MD, Macular Degeneration; PC, Platelet count; BMD, Bone mineral density; RBC, Red blood cell count; FFC, FEV1-FVC ratio; EC, Eosinophils count; FVC, Forced vital capacity; WBC, White blood cell count.

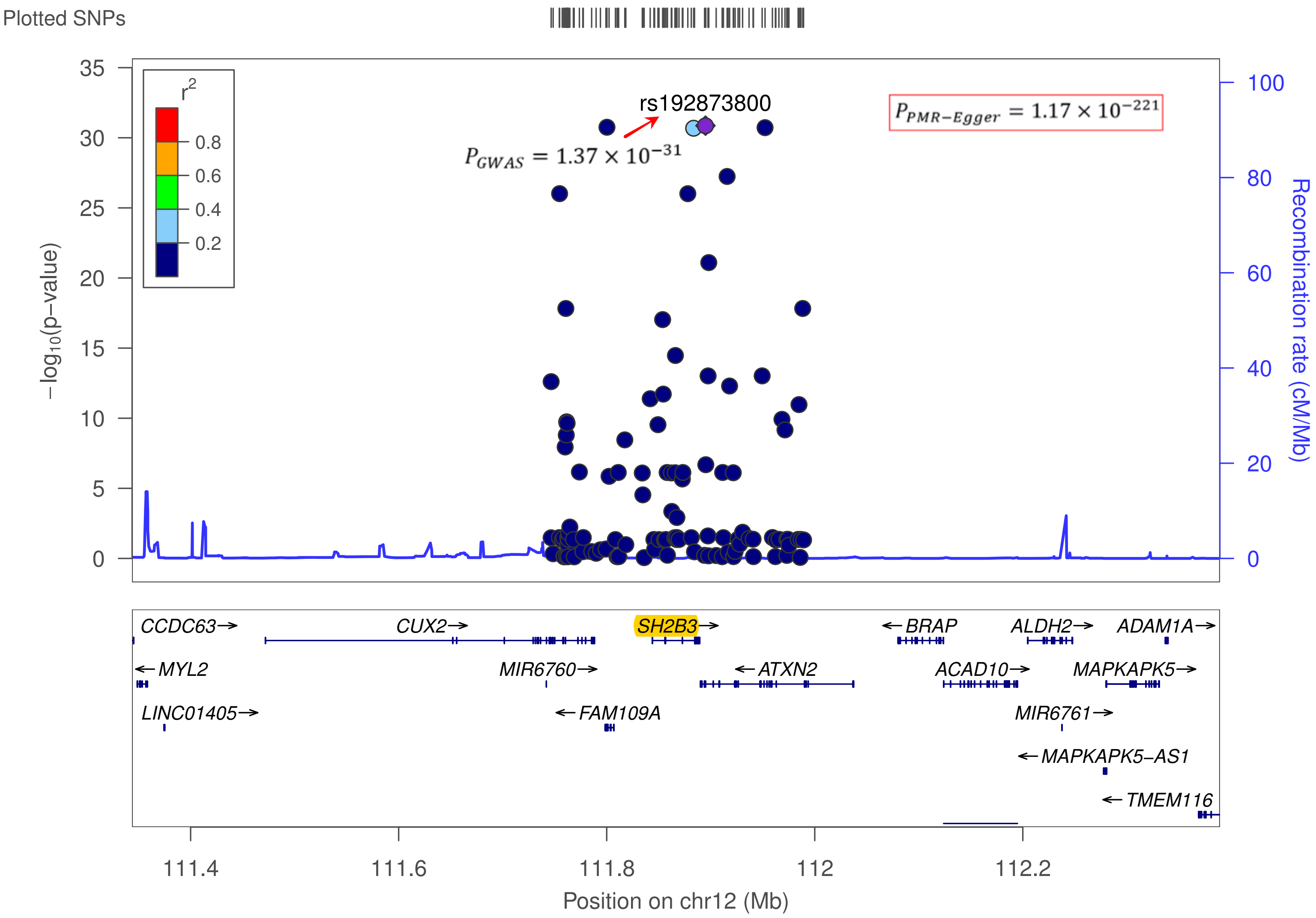

Supplementary Figure 35. Regional association plots with lead variants indicated by a purple diamond at 12q24.12. The association of an individual variant is plotted as -log10 *p*-value against chromosomal position. The y axis shows the recombination rate estimated from 1000 Genomes Project EUR data. The identified gene *SH2B3* by PMR-Egger for Platelet count is highlighted in yellow with *p*-value showing in the top right.

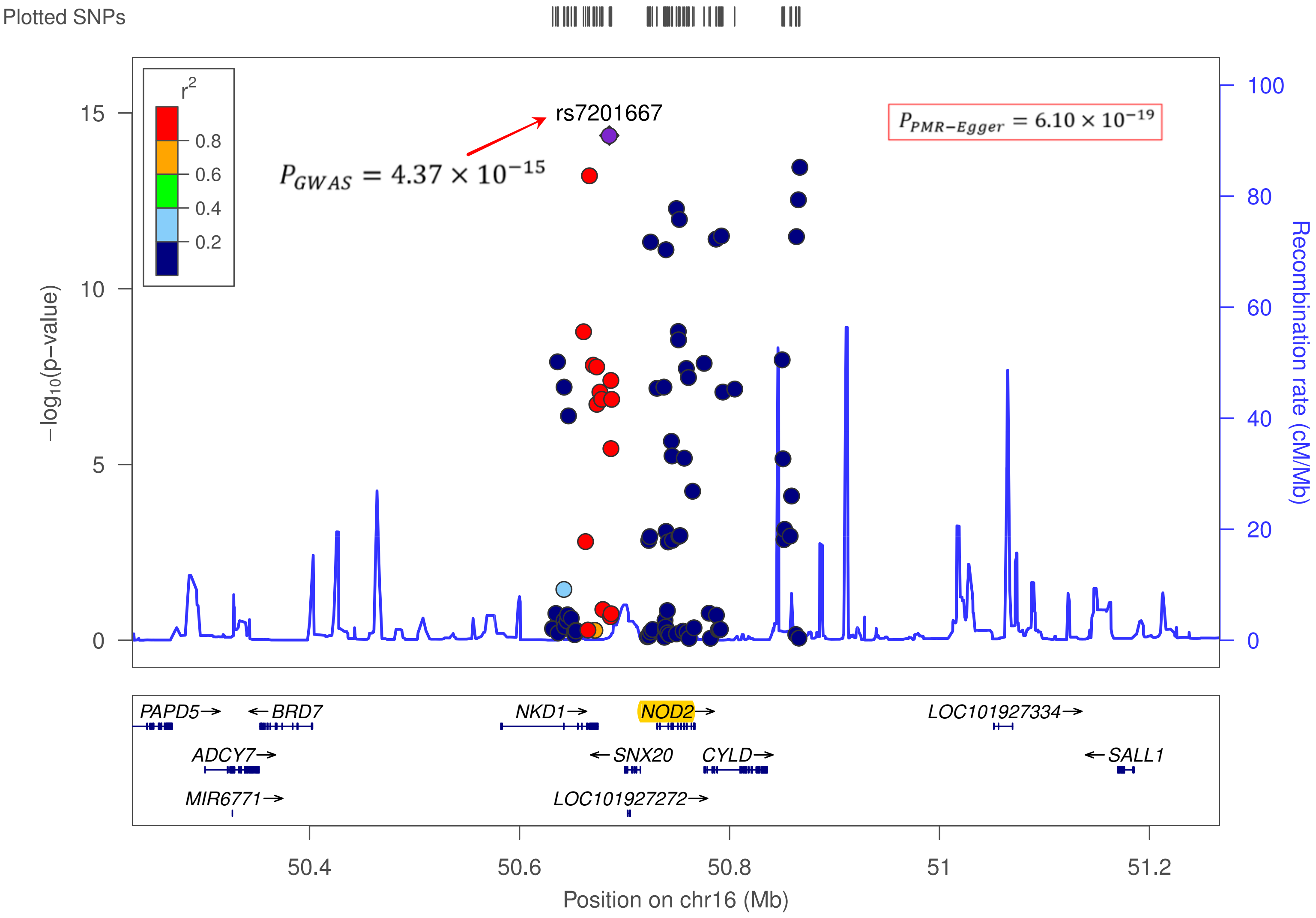

Supplementary Figure 36. Regional association plots with lead variants indicated by a purple diamond at 16q12.1. The association of an individual variant is plotted as -log10 *p*-values against chromosomal position. The y axis shows the recombination rate estimated from 1000 Genomes Project EUR data. The identified gene *NOD2* by PMR-Egger for Crohn's disease is highlighted in yellow with *p*-value showing in the top right.

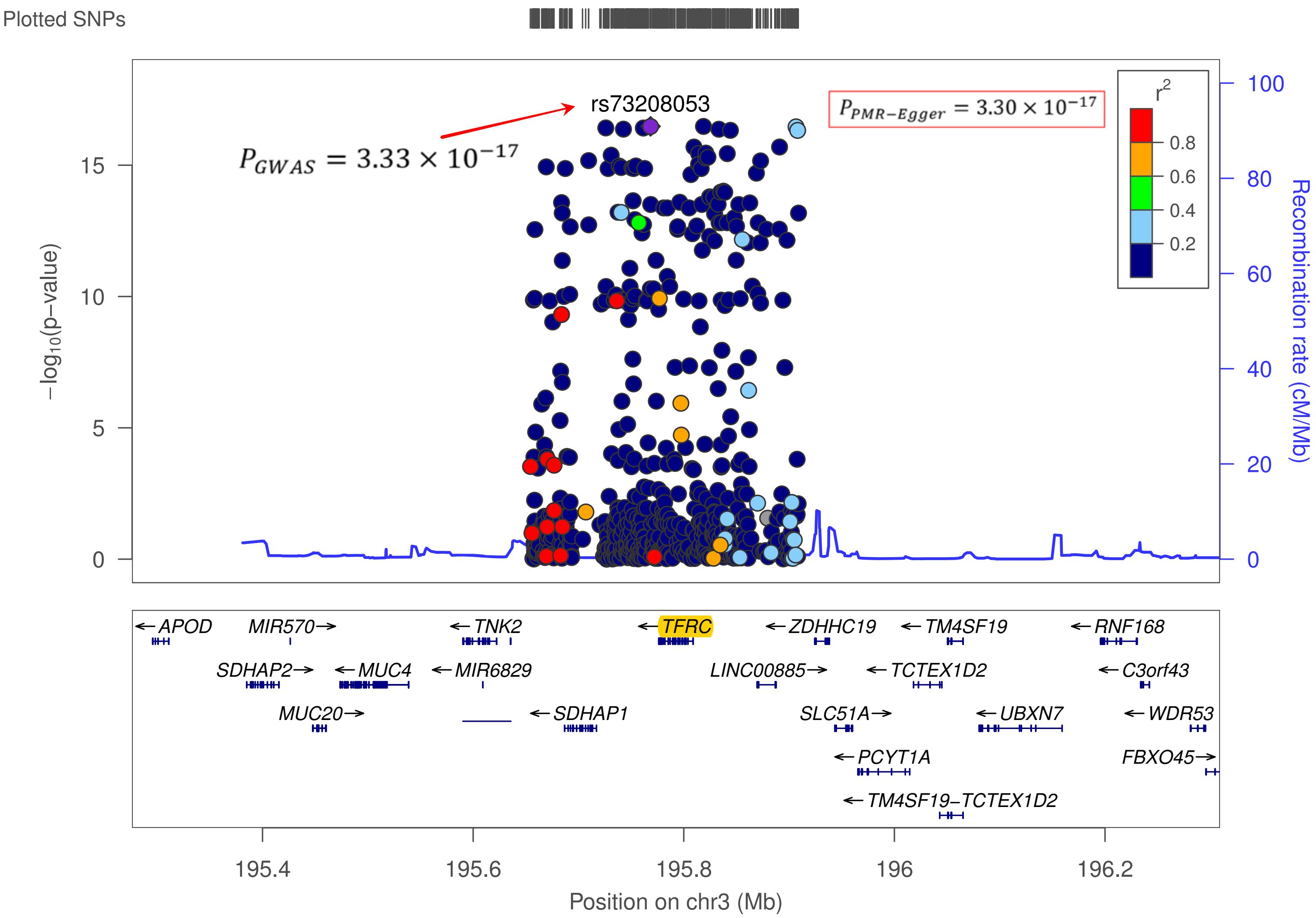

Supplementary Figure 37. Regional association plots with lead variants indicated by a purple diamond at 3q29. The association of an individual variant is plotted as -log10 *p*-value against chromosomal position. The y axis shows the recombination rate estimated from 1000 Genomes Project EUR data. The identified gene *TFRC* by PMR-Egger for RDW is highlighted in yellow with *p*-value showing in the top right.

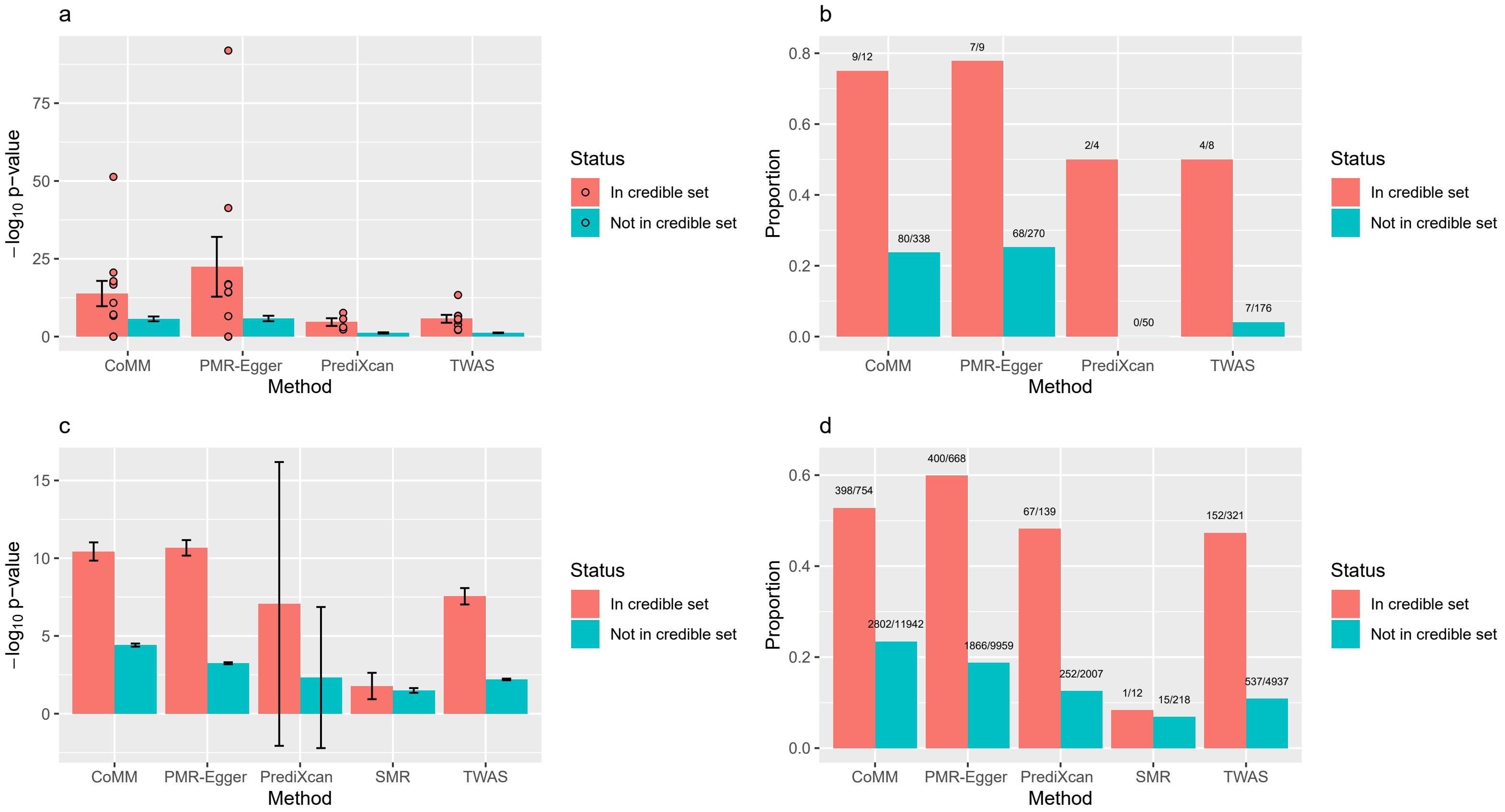

Supplementary Figure 38. Bar plots display comparison of standard TWAS/MR methods with the TWAS fine-mapping method FOCUS. Results are shown for the GERA data (**a**, **b**) and the UKBiobank data (**c**, **d**). (**a**, **c**): For each method (x-axis), we computed the average -log10 *p*-value for genes in the 90% FOCUS credible gene set (red bars) and the average -log10 *p*-value for genes outside the FOCUS credible gene set (green bars). For both (**a**) and (**c**), data are presented as mean±SEM. (**b**, **d**): For each method (x-axis), we also computed the proportion of genes in the 90% FOCUS credible gene set that are detected by the method (red bars) and the proportion of genes outside the FOCUS credible gene set detected by the method (green bars). The significant genes detected by each method is declared based on the Bonferroni threshold (0.05/15810). The number of significant genes/total genes in each category is also shown on top of the bars. Note that the number of total genes inside or outside the credible set is different across different methods, as FOCUS analyzes independent non-overlapping genomic regions that harbor at least one significant TWAS gene declared by the corresponding method. Overall, the PMR-Egger results are highly consistent with that of FOCUS, more so than the other methods.

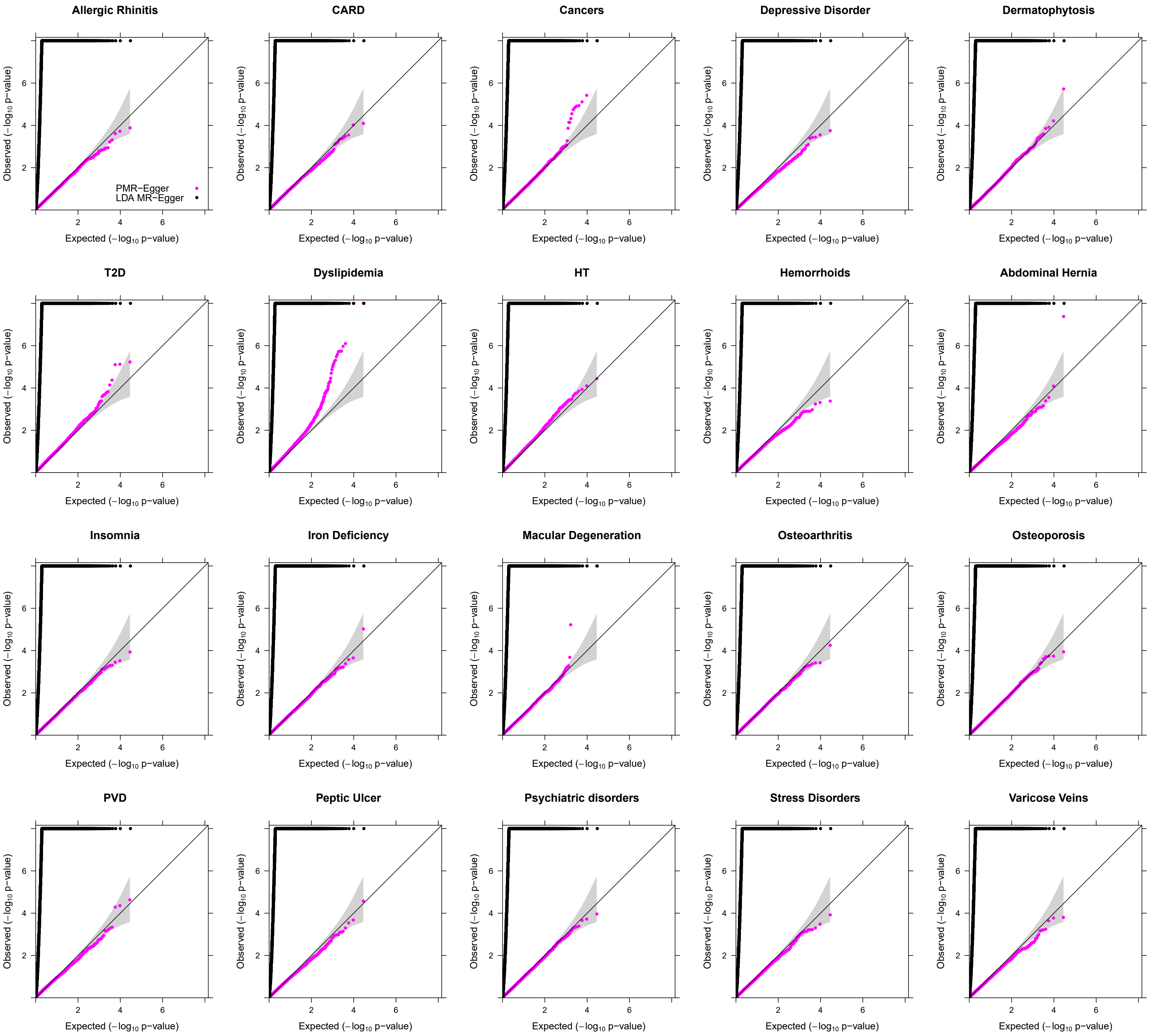

Supplementary Figure 39. Quantile-quantile plot of -log10 *p*-values from different methods for testing horizontal pleiotropic effect in the TWAS application to 20 traits in GERA. Compared methods include CoMM (turquoise), PMR-Egger (magenta), TWAS (blue), LDA MR-Egger (black), SMR (orange), and PrediXcan (purple).

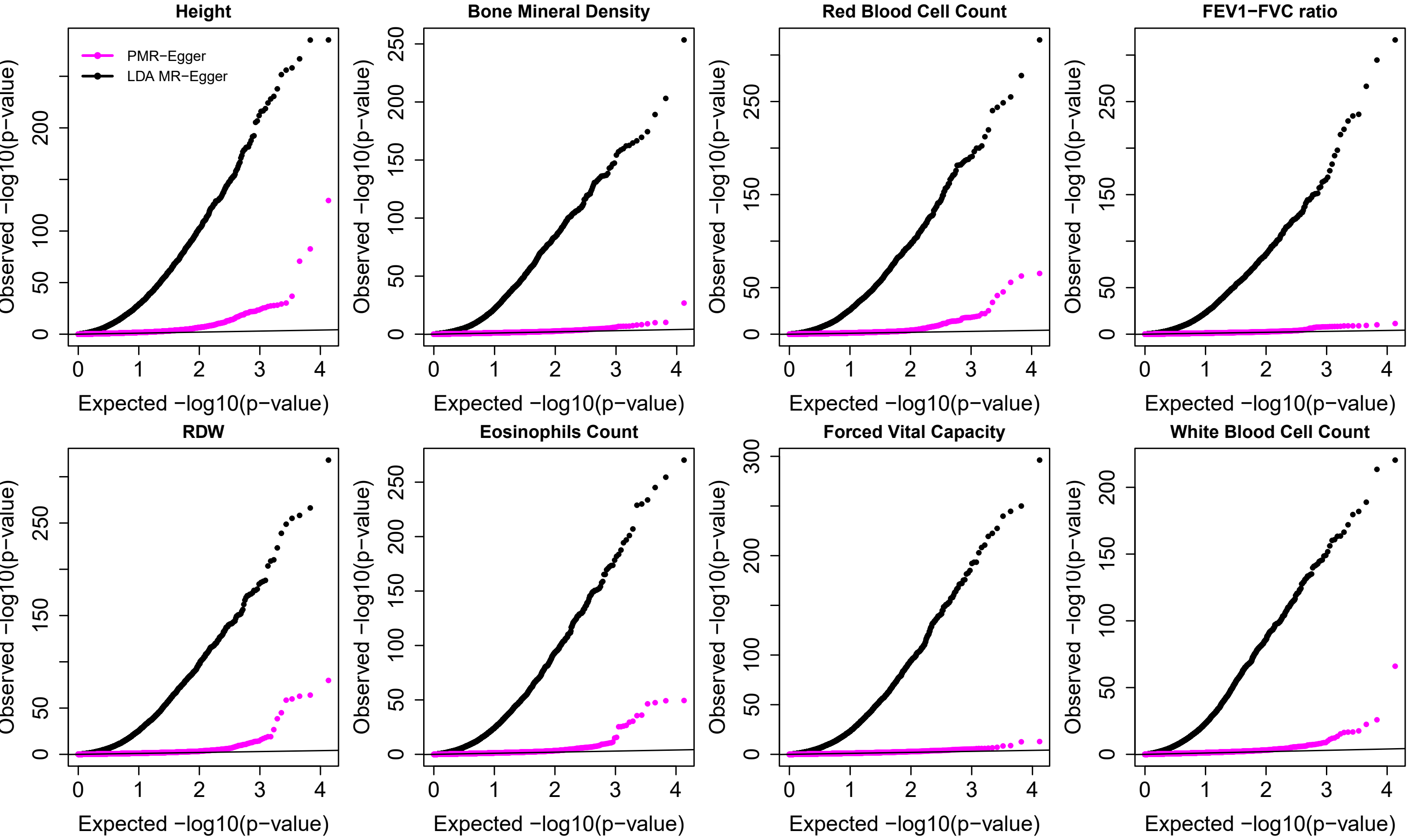

Supplementary Figure 40. Quantile-quantile plot of -log10 *p*-values from different methods for testing horizontal pleiotropic effect in the TWAS application to 8 traits in UK Biobank. Compared methods include CoMM (turquoise), PMR-Egger (magenta), TWAS (blue), LDA MR-Egger (black), SMR (orange), and PrediXcan (purple).

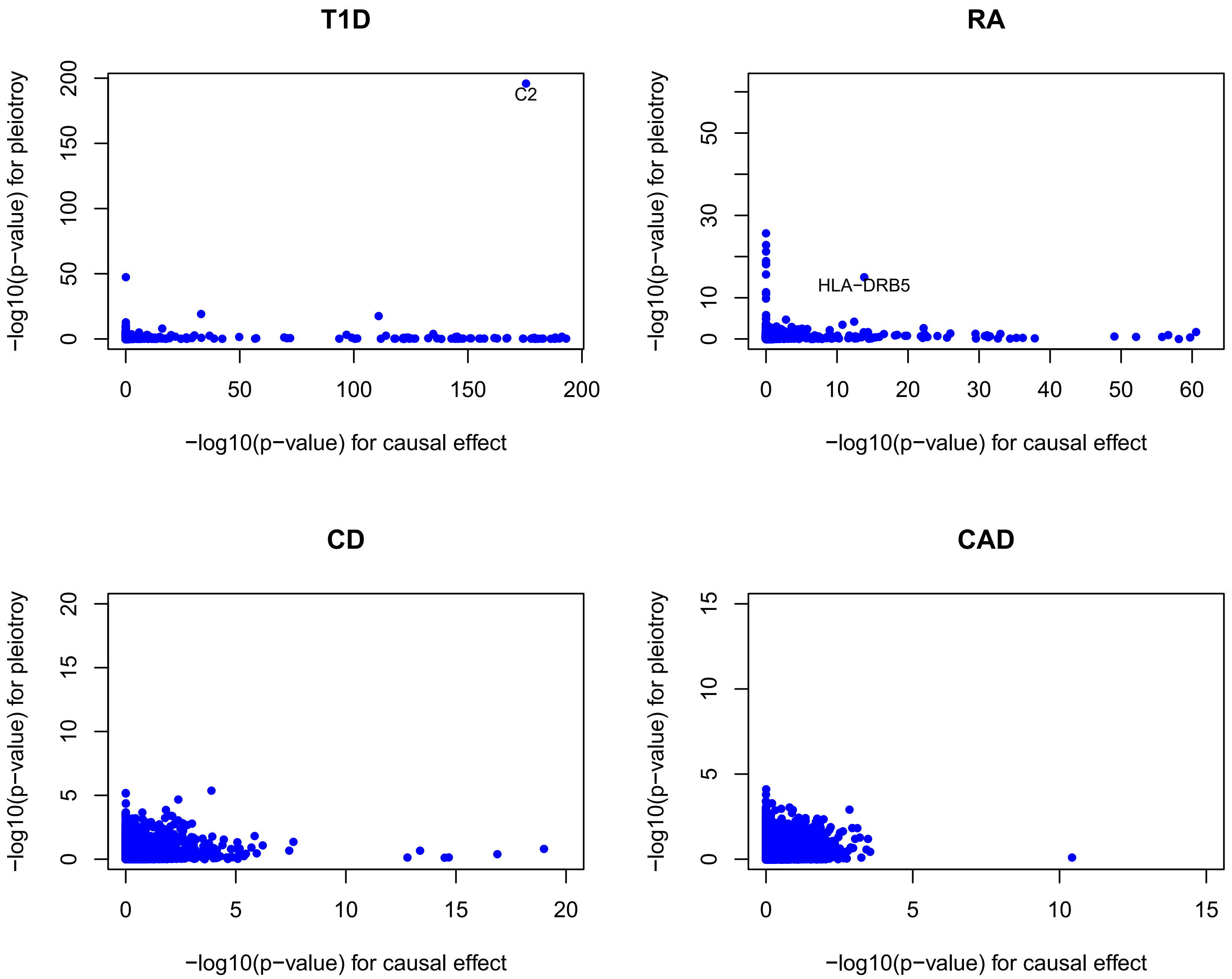

Supplementary Figure 41. Scatter plot of -log10 *p*-values by PMR-Egger for testing causal effect versus -log10 *p*-values by PMR-Egger for testing horizontal pleiotropic effect across genes for each of four traits in WTCCC. Each dot represents one gene. Only traits with at least one gene that has either a significant causal effect or a significant horizontal pleiotropic effect are displayed.

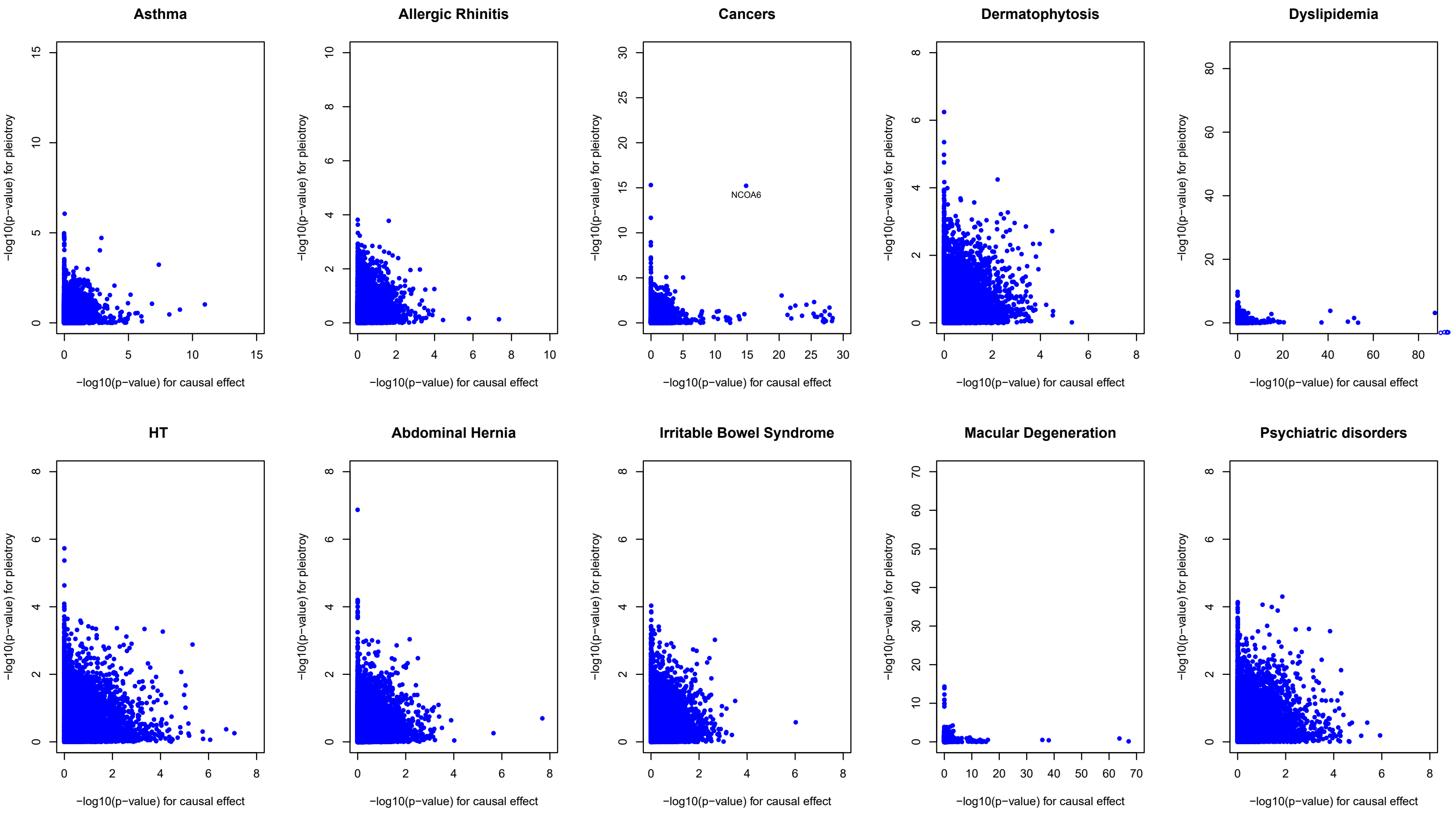

Supplementary Figure 42. Scatter plot of -log10 *p*-values by PMR-Egger for testing causal effect versus -log10 *p*-values by PMR-Egger for testing horizontal pleiotropic effect across genes for each of 10 traits in GERA. Each dot represents one gene. Only traits with at least one gene that has either a significant causal effect or a significant horizontal pleiotropic effect are displayed.

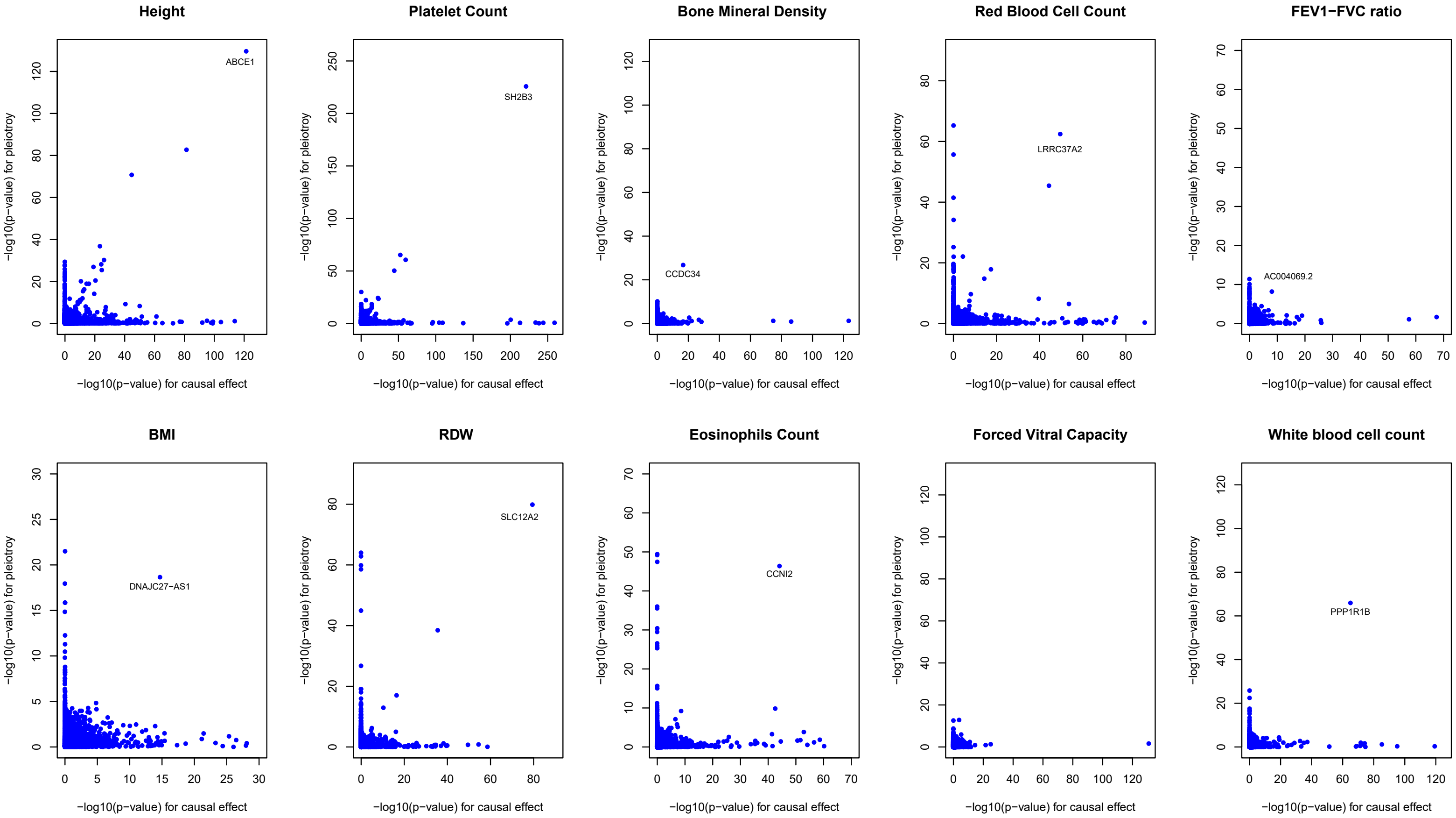

Supplementary Figure 43. Scatter plot of -log10 *p*-values by PMR-Egger for testing causal effect versus -log10 *p*-values by PMR-Egger for testing horizontal pleiotropic effect across genes for each of 10 traits in UK Biobank. Each dot represents one gene. Only traits with at least one gene that has either a significant causal effect or a significant horizontal pleiotropic effect are displayed.

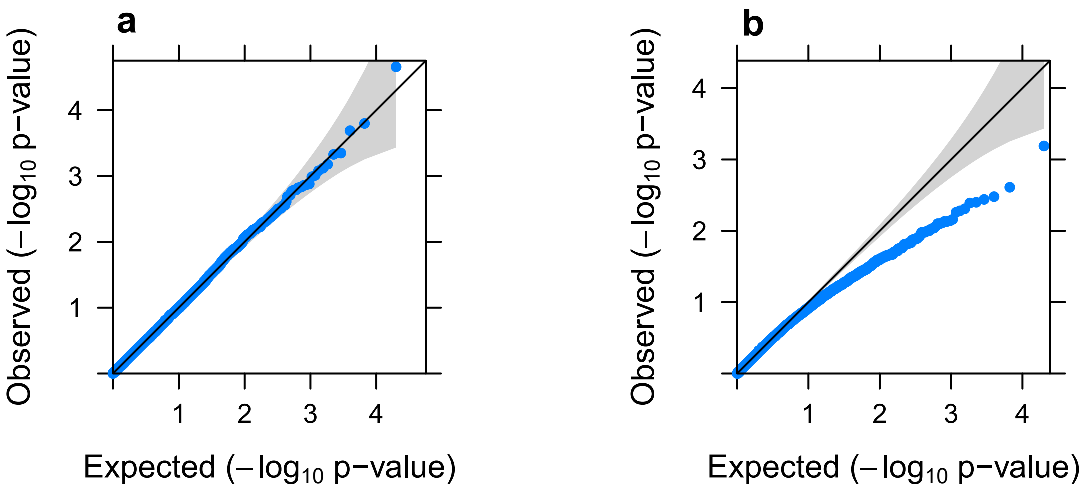

Supplementary Figure 44. Quantile-quantile plot of -log10 *p*-values for testing the causal effect in null simulations, under a variance component modeling assumption for the horizontal pleiotropic effects. In simulations, each γ follows the normal distribution with variance equals 0.001/556. The *p*-values are calibrated if we knew the true hyper-parameters (**a**). However, due to the uncertainty in hyper-parameter estimates, *p*-values become are overly conservative when we estimate hyper-parameters (**b**).

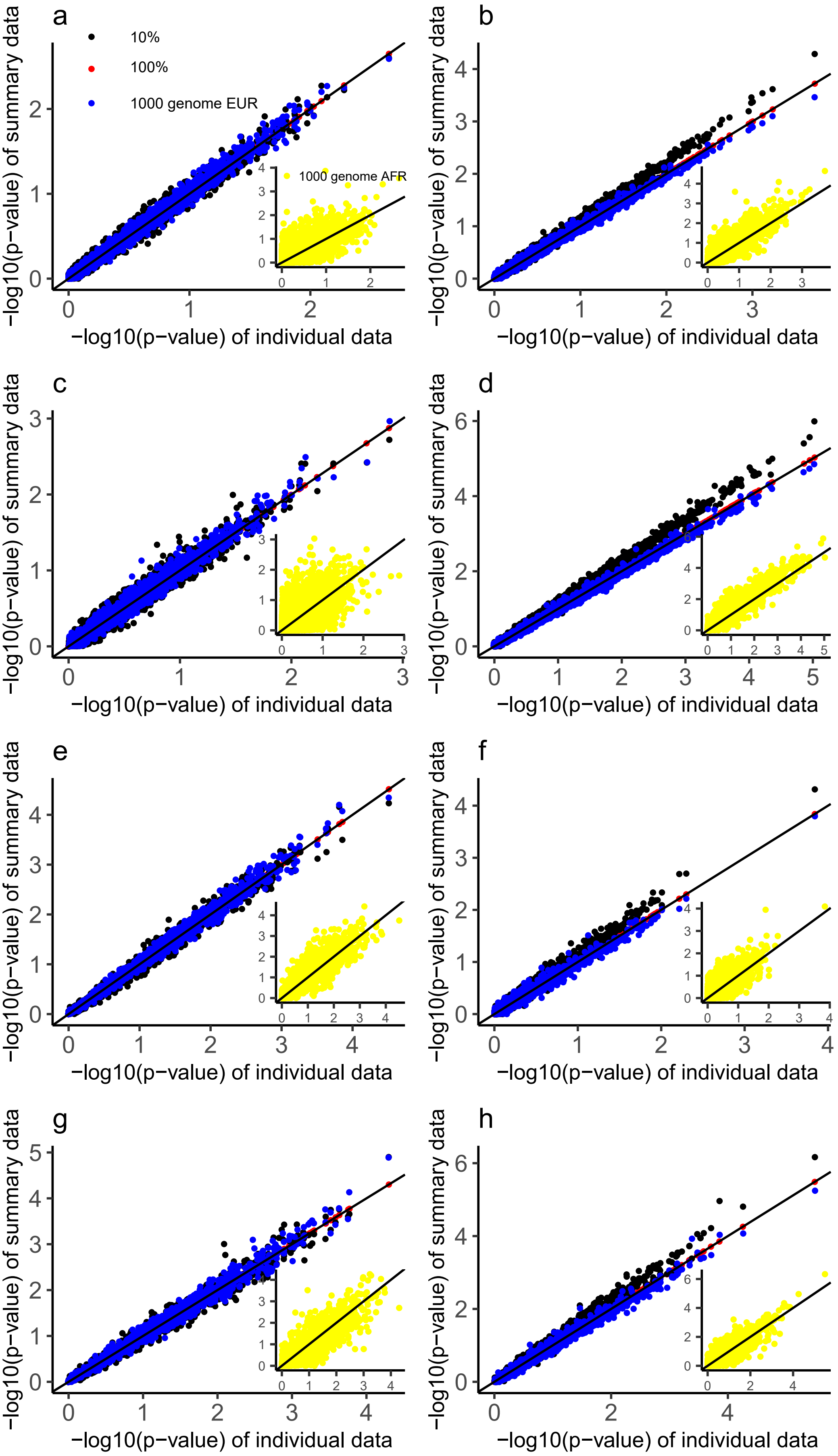

Supplementary Figure 45. Comparison of the summary-data version of PMR-Egger with the individual-data version of PMR-Egger. The LD matrix in the eQTL data is directly computed using the individual level from the eQTL study. The LD matrix in the GWAS data is computed through four different ways: by using individual-level genotypes from either all individuals in the GWAS (n=2,000; red), 10% of randomly selected individuals from the GWAS (n=200; black), the individuals with European ancestry from the 1,000 Genomes project (n=503; blue), or the individuals with African ancestry from the 1,000 Genomes project (n=611; inset; yellow). We examined both the causal gene association test (**a**, **c**, **e**, **g**) and the pleiotropy test (**b**, **d**, **f**, **h**). In each case, we plotted -log10 *p*-values from the summary-data version of PMR-Egger (y-axis) versus-log10 *p*-values from the summary-data version of PMR-Egger (x-axis). Simulations are performed under different causal effect and horizontal pleiotropic effect sizes: PVE_{zy}=0,γ=0 (**a**, **b**); PVE_{zy}=0,γ=0.0005 (**c**, **d**); PVE_{zy}=0.6%,γ=0 (**e**, **f**); PVE_{zy}=0.6%, γ=0.0005 (**g**, **h**). As expected, except for the African reference panel, the results from the summary-data version of PMR-Egger are consistent with that from the individual-data version of PMR-Egger.

Supplementary Tables

Supplementary Table 1. Genomic inflation factor for three GWAS data.

Trait		#Gene	Causal gene effect						Pleiotropy_effect	
			CoMM	PMR-Egger	TWAS	PrediXcan	LDA MR-Egger	SMR	PMR-Egger	LDA MR-Egger
WTCCC	T1D	15584	1.14	0.95	1.20	1.21	18.26	1.06	1.01	35.53
	CD	15584	1.14	1.04	1.23	1.24	17.73	1.09	0.97	34.59
	RA	15580	1.13	0.94	1.22	1.23	17.60	1.09	0.96	34.80
	BD	15582	1.22	0.94	1.25	1.26	17.76	1.02	0.96	34.22
	T2D	15583	1.14	0.95	1.22	1.21	18.09	0.98	1.01	36.00
	CAD	15584	1.20	0.93	1.28	1.26	18.56	0.94	0.93	34.00
	HT	15581	1.23	0.96	1.31	1.31	18.21	0.97	0.96	34.11
GERA	Asthma	15162	1.25	1.05	1.10	1.06	33.60	0.98	1.09	72.19
	Allergic Rhinitis	15154	1.11	0.95	1.06	1.05	32.13	0.99	0.96	72.18
	CARD	15146	1.12	0.96	1.09	1.11	33.74	1.02	0.99	72.19
	Cancers	15135	1.26	1.13	1.09	1.07	34.36	1.02	1.00	72.19
	Depressive Disorder	15131	0.95	1.02	1.05	1.02	33.95	0.96	0.93	70.01
	Dermatophytosis	15128	0.95	0.93	1.01	1.02	33.64	0.95	1.05	72.18
	T2D	15123	1.26	0.98	1.15	1.10	34.23	1.05	1.02	72.19
	Dyslipidemia	15119	1.13	1.07	1.14	1.16	32.89	1.05	1.06	72.19
	HT	15117	1.62	0.99	1.16	1.15	34.74	1.05	1.04	72.19
	Hemorrhoids	15110	1.02	0.95	1.05	1.03	33.40	0.97	0.95	72.19
	Abdominal Hernia	15104	0.99	1.01	1.03	1.03	33.65	0.97	1.03	72.19
	Insomnia	15094	0.94	0.96	1.06	1.07	33.71	0.96	1.04	72.19
	Iron Deficiency	15088	1.09	0.93	1.05	1.05	33.54	0.98	0.97	72.19
	Irritable Bowel Syndrome	15080	0.97	0.95	1.02	1.01	33.02	0.96	1.04	72.18
	Macular Degeneration	15075	1.18	1.08	1.03	1.03	33.40	0.99	1.04	72.19
	Osteoarthritis	15072	0.94	0.93	1.08	1.06	33.51	1.01	0.96	72.19
	Osteoporosis	15069	1.08	0.92	1.04	1.03	32.33	0.97	1.05	72.19
	PVD	15065	1.11	0.93	1.01	1.02	33.30	0.97	1.05	72.19
	Peptic Ulcer	15061	0.96	0.94	1.03	1.03	33.64	0.95	0.93	72.18
	Psychiatric disorders	15057	0.94	0.95	1.07	1.07	33.89	1.01	1.01	69.82
	Stress Disorders	15055	1.07	0.92	0.98	1.00	32.47	0.96	0.92	72.19
	Varicose Veins	15051	1.01	0.96	1.06	1.02	33.34	0.94	0.93	72.19
UKBiobank	Height	15761	1.61	1.18	2.17	1.46	16.65	1.71	1.71	29.85
	Platelet Count	15765	1.87	1.31	1.46	1.26	15.25	1.41	1.45	26.07
	Bone Mineral Density	15761	1.45	1.13	1.27	1.1	10.48	1.13	1.13	17.75
	Red Blood Cell Count	15751	1.71	1.18	1.64	1.22	14.12	1.36	1.38	23.45
	FEV1-FVC ratio	15747	1.59	1.17	1.3	1.11	12.91	1.19	1.19	19.48
	BMI	14633	1.68	1.12	1.38	1.16	14.59	1.29	1.35	25.95
	RDW	15744	1.65	1.16	1.42	1.09	13.93	1.18	1.22	22.59
	Eosinophils Count	15740	1.9	1.34	1.46	1.14	14.33	1.23	1.31	22.42
	Forced Vital Capacity	15735	1.78	1.3	1.38	1.13	13.01	1.25	1.14	22.17
	White blood cell count	15729	1.76	1.25	1.38	1.12	13.93	1.17	1.32	22.89

Supplementary Table 2. Number of significant genes for three GWAS data under Bonferroni correction.

Trait		#Gene	Causal gene effect						Pleiotropy_effect	
			CoMM	PMR-Egger	TWAS	PrediXcan	LDA MR-Egger	SMR	PMR-Egger	LDA MR-Egger
WTCCC	T1D	15584	151	137	62	42	4539	4	11	6860
	CD	15584	14	10	7	7	4586	0	0	6797
	RA	15580	81	81	16	10	4601	1	11	6773
	BD	15582	0	0	0	0	4712	0	0	6890
	T2D	15583	1	0	1	0	4565	0	0	6851
	CAD	15584	1	1	1	1	4953	0	0	7206
	HT	15581	0	0	0	0	5512	0	0	8020
GERA	Asthma	15162	6	5	3	4	6468	4	0	8928
	Allergic Rhinitis	15154	3	2	0	1	6356	0	0	9010
	CARD	15146	0	0	0	0	6358	0	0	8943
	Cancers	15135	34	28	8	4	6500	1	1	9017
	Depressive Disorder	15131	0	0	0	0	6480	0	0	8752
	Dermatophytosis	15128	0	0	0	0	6465	0	1	8876
	T2D	15123	3	1	2	1	6529	0	0	8979
	Dyslipidemia	15119	63	57	11	8	6392	0	10	9139
	HT	15117	4	3	1	0	6564	0	0	9042
	Hemorrhoids	15110	0	0	0	0	6422	0	0	8801
	Abdominal Hernia	15104	2	2	0	0	6508	0	1	8894
	Insomnia	15094	0	0	1	0	6483	0	0	8884
	Iron Deficiency	15088	0	0	0	0	6451	0	0	8855
	Irritable Bowel Syndrome	15080	0	0	0	0	6384	0	0	8918
	Macular Degeneration	15075	32	28	1	2	6458	0	8	8900
	Osteoarthritis	15072	0	0	0	0	6460	0	0	8895
	Osteoporosis	15069	1	0	0	0	6404	0	0	8797
	PVD	15065	1	1	0	0	6386	0	0	8909
	Peptic Ulcer	15061	0	0	0	0	6449	0	0	8888
	Psychiatric disorders	15057	0	0	0	0	6443	0	0	8693
	Stress Disorders	15055	0	0	0	0	6318	0	0	8834
	Varicose Veins	15051	0	0	1	0	6464	0	0	8945
UKBiobank	Height	15761	1295	1008	285	172	3802	26	170	5509
	Platelet Count	15765	645	499	155	101	3606	13	123	5171
	Bone Mineral Density	15761	146	108	29	14	2933	3	15	4036
	Red Blood Cell Count	15751	497	353	145	81	3417	8	92	4928
	FEV1-FVC ratio	15747	97	51	28	12	3258	1	27	4520
	BMI	14633	240	173	47	31	3533	2	28	4971
	RDW	15744	247	183	78	51	3413	7	57	4835
	Eosinophils Count	15740	295	223	111	48	3548	8	57	4800
	Forced Vital Capacity	15735	118	82	26	16	3243	3	11	4574
	White blood cell count	15729	182	116	54	34	3363	4	46	4793

Supplementary Table 3. Summary of gene-based fine mapping results in three GWAS data by FOCUS

Trait		#Analyzed regions	#Analyzed regions with multivariate TWAS- significant gene	#TWAS-significant gene in analyzed regions	#genes in analyzed regions	#genes in 90% credible gene sets
WTCCC	T1D	7	6	28	210	4
	CD	3	2	7	24	1
	RA	2	2	11	42	1
	CAD	1	1	1	12	0
GERA	Allergic Rhinitis	1	1	2	5	0
	Cancers	4	4	27	81	4
	Dyslipidemia	9	9	42	167	8
	Abdominal Hernia	1	1	2	9	1
	Macular Degeneration	2	2	4	15	0
UKBiobank	Height	169	150	946	3559	247
	Platelet Count	86	75	462	1991	140
	Bone Mineral Density	28	24	97	461	32
	Red Blood Cell Count	56	50	343	1426	89
	FEV1-FVC ratio	15	13	47	309	20
	BMI	33	30	171	923	62
	RDW	45	33	199	1196	76
	Eosinophils Count	48	38	210	1223	54
	Forced Vital Capacity	15	14	83	422	30
	White blood cell count	20	19	127	495	29

Summary of fine mapping results from FOCUS in three GWAS data sets. For each trait in turn (listed in rows), we performed FOCUS analysis on independent non-overlapping genomic regions that harbor on least one genome-wide-significant SNP ($p < 5 \times 10^{-8}$) and at least a significant TWAS gene under Bonferroni correction. In this table, the significant TWAS genes are declared based on PMR-Egger results; the other parallel FOCUS analyses based on the other corresponding MR methods are not shown in this table. The table lists the number of analyzed regions (3rd column), number of analyzed regions that contain multiple significant genes detected by PMR-Egger (4th column), number of significant TWAS genes in the analyzed regions (5th column), the number of total genes analyzed in these regions (6th column) and the number of genes in the 90% credible set by FOCUS (7th column).

Supplementary References

1 Liu, C., Rubin, D. B. & Wu, Y. N. Parameter expansion to accelerate EM: the PX-EM algorithm. *Biometrika* **85**, 755-770 (1998).

2 Berzuini, C., Guo, H., Burgess, S. & Bernardinelli, L. A Bayesian approach to Mendelian randomization with multiple pleiotropic variants. *Biostatistics*, 1-16 (2018).

3 Berzuini, C., Dawid, P. & Bernardinell, L. *Causality: Statistical perspectives and applications*. (John Wiley & Sons, 2012).

4 Dawid, A. P. Causal inference without counterfactuals. *Journal of the American statistical Association* **95**, 407-424 (2000).

5 Dawid, A. P. Statistical causality from a decision-theoretic perspective. *Annual Review of Statistics and Its Application* **2**, 273-303 (2015).

6 Gusev, A. *et al.* Integrative approaches for large-scale transcriptome-wide association studies. *Nature genetics* **48**, 245-252 (2016).

7 Gamazon, E. R. *et al.* A gene-based association method for mapping traits using reference transcriptome data. *Nature genetics* **47**, 1091-1098 (2015).

8 Yang, C. *et al.* CoMM: a collaborative mixed model to dissecting genetic contributions to complex traits by leveraging regulatory information. *Bioinformatics*, doi:10.1093/bioinformatics/bty865 (2018).

9 Zeng, P. & Zhou, X. Non-parametric genetic prediction of complex traits with latent Dirichlet process regression models. *Nature communications* **8**, 456 (2017).

10 Nagpal, S. *et al.* TIGAR: An Improved Bayesian Tool for Transcriptomic Data Imputation Enhances Gene Mapping of Complex Traits. *The American Journal of Human Genetics* (2019).

11 Zhou, X., Carbonetto, P. & Stephens, M. Polygenic Modeling with Bayesian Sparse Linear Mixed Models. *Plos Genetics* **9**, : e1003264. (2013).

12 Zhu, Z. *et al.* Integration of summary data from GWAS and eQTL studies predicts complex trait gene targets. *Nature genetics* **48**, 481-487 (2016).

13 Kanai, M. et al. Genetic analysis of quantitative traits in the Japanese population links cell types to complex human diseases. Nat Genet 50, 390-400, doi:10.1038/s41588-018-0047-6 (2018).

14 Astle, W. J. et al. The Allelic Landscape of Human Blood Cell Trait Variation and Links to Common Complex Disease. Cell 167, 1415-1429 e1419, doi:10.1016/j.cell.2016.10.042 (2016).

15 Wood, A. R. et al. Defining the role of common variation in the genomic and biological architecture of adult human height. Nat Genet 46, 1173-1186, doi:10.1038/ng.3097 (2014).

16 Wainberg, M. et al. Opportunities and challenges for transcriptome-wide association studies. Nat Genet 51, 592-599, doi:10.1038/s41588-019-0385-z (2019).

17 Mancuso, N. et al. Probabilistic fine-mapping of transcriptome-wide association studies. Nat Genet 51, 675-682, doi:10.1038/s41588-019-0367-1 (2019).
