# Supplementary material for: Testing and controlling for horizontal pleiotropy with probabilistic Mendelian randomization in transcriptome-wide association studies
Source: Nat Commun. 2020 Jul 31;11:3861. doi: 10.1038/s41467-020-17668-6 (PMC7395774; doi:10.1038/s41467-020-17668-6)
Supplement: Supplementary file 3 — Reporting Summary [file 41467_2020_17668_MOESM3_ESM.pdf]

nature research

Corresponding author(s): Xiang Zhou

Last updated by author(s): Jun 25, 2020

Reporting Summary

Nature Research wishes to improve the reproducibility of the work that we publish. This form provides structure for consistency and transparency in reporting. For further information on Nature Research policies, seeAuthors & Referees and theEditorial Policy Checklist .

Statistics

For all statistical analyses, confirm that the following items are present in the figure legend, table legend, main text, or Methods section.

n/a	Confirmed	
☐	☒	The exact sample size (n) for each experimental group/condition, given as a discrete number and unit of measurement
☐	☒	A statement on whether measurements were taken from distinct samples or whether the same sample was measured repeatedly
☐	☒	The statistical test(s) used AND whether they are one- or two-sided<br>*Only common tests should be described solely by name; describe more complex techniques in the Methods section.*
☐	☒	A description of all covariates tested
☐	☒	A description of any assumptions or corrections, such as tests of normality and adjustment for multiple comparisons
☐	☒	A full description of the statistical parameters including central tendency (e.g. means) or other basic estimates (e.g. regression coefficient) AND variation (e.g. standard deviation) or associated estimates of uncertainty (e.g. confidence intervals)
☐	☒	For null hypothesis testing, the test statistic (e.g. F, t, r) with confidence intervals, effect sizes, degrees of freedom and P value noted<br>*Give P values as exact values whenever suitable.*
☐	☒	For Bayesian analysis, information on the choice of priors and Markov chain Monte Carlo settings
☐	☒	For hierarchical and complex designs, identification of the appropriate level for tests and full reporting of outcomes
☐	☒	Estimates of effect sizes (e.g. Cohen's d, Pearson's r), indicating how they were calculated

Our web collection on statistics for biologists contains articles on many of the points above.

Software and code

Policy information about availability of computer code

Data collection	The present study does not involve data collection. We used publicly available data sets to validate the effectiveness of our proposed method. No software was used for data collection.
Data analysis	We used the newly developed R package PMR for data analysis. PMR is described in the Methods section and deposited at [http://www.xzlab.org/software.html] and at Github [https://github.com/yuanzhongshang/PMR]. In addition, we also used the following software for comparative analysis in simulations.<br>glmnet[https://cran.r-project.org/web/packages/glmnet/index.html](R version 3.6.3): an extremely efficient procedures for fitting the elastic-net regularization path for linear regression.<br>GEMMA[http://xzlab.org/software.html](version 0.96): A genome-wide efficient mixed model association algorithm for a linear mixed model and some of its close relatives for GWAS.<br>CoMM[https://github.com/gordonliu810822/CoMM](version 1.0): a collaborative mixed model to dissecting genetic contributions to complex traits by leveraging regulatory information.<br>MR-PRESSO[https://github.com/rondolab/MR-PRESSO](version 1.0): A method that allows for the evaluation of horizontal pleiotropy in multi-instrument mendelian randomization utilizing genome-wide summary association statistics.<br>Minimac3[https://genome.sph.umich.edu/wiki/Minimac3](version 2.0.1), is a lower memory and more computationally efficient implementation of the genotype imputation algorithms which is designed to handle very large reference panels in a more computationally efficient way with no loss of accuracy.<br>IMPUTE2[https://mathgen.stats.ox.ac.uk/impute/impute_v2.html](version 2), a flexible and accurate genotype imputation method for the next generation of genome-wide association studies.<br>SHAPEIT[https://mathgen.stats.ox.ac.uk/genetics_software/shapeit/shapeit.html](version v2.r900),a fast and accurate method for estimation of haplotypes from genotype or sequencing data.

For manuscripts utilizing custom algorithms or software that are central to the research but not yet described in published literature, software must be made available to editors/reviewers. We strongly encourage code deposition in a community repository (e.g. GitHub). See the Nature Research guidelines for submitting code & software for further information.

Data

Policy information about availability of data

All manuscripts must include a data availability statement. This statement should provide the following information, where applicable:

- Accession codes, unique identifiers, or web links for publicly available datasets
- A list of figures that have associated raw data
- A description of any restrictions on data availability

The study made use of the publicly available data. The GEUVADIS gene expression data is publicly available at [http://www.geuvadis.org]. The WTCCC genotype and phenotype data is publicly available at [https://www.wtccc.org.uk]. The GERA genotype and phenotype data is available at [https://www.ncbi.nlm.nih.gov/gap] with dbGaP accession number phs000788. The UK Biobank data is from UK Biobank resource at [https://mrc.ukri.org/research/facilities-and-resources-for-researchers/biobank/] under Application Number 30686.
All data are available on the aforementioned public repository and are accessible with permission from the corresponding data committee. No restrictions on data availability other than those imposed by the corresponding data committee.

Field-specific reporting

Please select the one below that is the best fit for your research. If you are not sure, read the appropriate sections before making your selection.

☒ Life sciences ☐ Behavioural & social sciences ☐ Ecological, evolutionary & environmental sciences

For a reference copy of the document with all sections, see nature.com/documents/nr-reporting-summary-flat.pdf

Life sciences study design

All studies must disclose on these points even when the disclosure is negative.

Sample size	No data collection was involved in the present study. We used data downloaded from public websites and used all samples in these data. The sample size determination information, if available, were described in the original papers. These original papers are cited in the present study and include the gene expression study (ref. 52) and the three GWAS studies (refs. 53,54,57,58).
Data exclusions	No data were excluded from the study.
Replication	This is a methodology paper but not an analysis paper. Therefore, we did not perform replication using experiments. Instead, we followed existing literature to compare the type I error control and power of different methods. In addition, we cross-validated the findings of the present study by comparing to other published large-scale GWAS results with details described in the results section.
Randomization	Randomization is not relevant to the present study. No data collection was involved in the present study. We used data downloaded from public websites. All randomization information, if available, were described in the original papers. These original papers are cited in the present study and include the gene expression study (ref. 52) and the three GWAS studies (refs. 53,54,57,58).
Blinding	Blinding is not relevant to the present study. No data collection was involved in the present study. We used data downloaded from public websites. All blinding information, if available, were described in the original papers. These original papers are cited in the present study and include the gene expression study (ref. 52) and the three GWAS studies (refs. 53,54,57,58).

Reporting for specific materials, systems and methods

We require information from authors about some types of materials, experimental systems and methods used in many studies. Here, indicate whether each material, system or method listed is relevant to your study. If you are not sure if a list item applies to your research, read the appropriate section before selecting a response.

Materials & experimental systems

n/a	Involved in the study	
☒	☐	Antibodies
☒	☐	Eukaryotic cell lines
☒	☐	Palaeontology
☒	☐	Animals and other organisms
☒	☐	Human research participants
☒	☐	Clinical data

Methods

n/a	Involved in the study	
☒	☐	ChIP-seq
☒	☐	Flow cytometry
☒	☐	MRI-based neuroimaging
